# Age‐dependent expression of *DNMT1* and *DNMT3B* in PBMCs from a large European population enrolled in the MARK‐AGE study

**DOI:** 10.1111/acel.12485

**Published:** 2016-05-11

**Authors:** Fabio Ciccarone, Marco Malavolta, Roberta Calabrese, Tiziana Guastafierro, Maria Giulia Bacalini, Anna Reale, Claudio Franceschi, Miriam Capri, Antti Hervonen, Mikko Hurme, Beatrix Grubeck‐Loebenstein, Bernhard Koller, Jürgen Bernhardt, Christiane Schӧn, P. Eline Slagboom, Olivier Toussaint, Ewa Sikora, Efstathios S. Gonos, Nicolle Breusing, Tilman Grune, Eugène Jansen, Martijn Dollé, María Moreno‐Villanueva, Thilo Sindlinger, Alexander Bürkle, Michele Zampieri, Paola Caiafa

**Affiliations:** ^1^ Faculty of Pharmacy and MedicineDepartment of Cellular Biotechnologies and HematologySapienza University of RomeRome00161Italy; ^2^ Pasteur Institute‐Fondazione Cenci BolognettiRome00161Italy; ^3^ National Institute of Health and Science on Aging (INRCA)Nutrition and Ageing CentreScientific and Technological Research Area60100AnconaItaly; ^4^ Department of Experimental, Diagnostic and Specialty MedicineAlma Mater Studiorum‐University of BolognaBologna40126Italy; ^5^ CIG‐Interdepartmental Center ‘L. Galvani’Alma Mater StudiorumUniversity of Bologna40126BolognaItaly; ^6^ The School of MedicineThe University of Tampere33014TampereFinland; ^7^ Research Institute for Biomedical Aging ResearchUniversität InnsbruckRennweg, 106020InnsbruckAustria; ^8^ Department for Internal MedicineUniversity Teaching Hospital Hall in TirolMilserstr. 106060Hall in TirolAustria; ^9^ BioTeSys GmbH73728EsslingenGermany; ^10^ Department of Molecular EpidemiologyLeiden University Medical CentreLeidenThe Netherlands; ^11^ Research Unit on Cellular BiologyUniversity of NamurRue de Bruxelles, 61NamurB‐5000Belgium; ^12^ Laboratory of the Molecular Bases of AgeingNencki Institute of Experimental BiologyPolish Academy of Sciences3 Pasteur Street02‐093WarsawPoland; ^13^ National Hellenic Research FoundationInstitute of BiologyMedicinal Chemistry and BiotechnologyAthensGreece; ^14^ Institute of Nutritional Medicine (180c)University of HohenheimFruwirthstraße 1270599StuttgartGermany; ^15^ German Institute of Human Nutrition Potsdam‐Rehbruecke (DIfE)Arthur‐Scheunert‐Allee 114‐11614558NuthetalGermany; ^16^ Centre for Health ProtectionNational Institute for Public Health and the EnvironmentPO Box 13720BA BilthovenThe Netherlands; ^17^ Molecular Toxicology GroupDepartment of BiologyUniversity of Konstanz78457KonstanzGermany; ^18^ Present address: Department of BiologyUniversity of Rome ‘Tor Vergata’Via della Ricerca Scientifica 100133RomeItaly

**Keywords:** aging, DNA methylation, *DNMT1*, *DNMT3B*

## Abstract

Aging is associated with alterations in the content and patterns of DNA methylation virtually throughout the entire human lifespan. Reasons for these variations are not well understood. However, several lines of evidence suggest that the epigenetic instability in aging may be traced back to the alteration of the expression of DNA methyltransferases. Here, the association of the expression of DNA methyltransferases *DNMT1* and *DNMT3B* with age has been analysed in the context of the MARK‐AGE study, a large‐scale cross‐sectional study of the European general population. Using peripheral blood mononuclear cells, we assessed the variation of *DNMT1* and *DNMT3B* gene expression in more than two thousand age‐stratified women and men (35–75 years) recruited across eight European countries. Significant age‐related changes were detected for both transcripts. The level of *DNMT1* gradually dropped with aging but this was only observed up to the age of 64 years. By contrast, the expression of *DNMT3B* decreased linearly with increasing age and this association was particularly evident in females. We next attempted to trace the age‐related changes of both transcripts to the influence of different variables that have an impact on changes of their expression in the population, including demographics, dietary and health habits, and clinical parameters. Our results indicate that age affects the expression of *DNMT1* and *DNMT3B* as an almost independent variable in respect of all other variables evaluated.

## Introduction

Epigenetic processes are a molecular interface that mediates the interaction between genome and environment during the entire lifespan of organisms. Aberrant epigenetic signalling, including DNA methylation defects, plays a crucial role in aging (Zampieri *et al*., 2015). The understanding of the mechanisms behind these events is an important research topic as it can reveal the molecular mechanisms contributing to age‐associated physiological decline and disease.

DNA methylation is a modification of the genome that occurs after DNA replication by the attachment of a methyl group to the cytosine of a CpG dinucleotide. In mammals, 5‐methylcytosine (5mC) represents an epigenetic modification of the genome that marks transcriptionally repressed domains and serves as a heritable signal sufficient to restore silent chromatin following DNA replication (Bird, 2002). In the human genome, almost 80% of the CpGs are methylated in nonrandom fashion with the remaining unmethylated residues preferentially restricted to CpG islands (CGIs) within gene promoters (Deaton & Bird, 2011). This bimodal pattern is established during development and differentiation when tissue‐specific changes in DNA methylation shape the epigenetic patterns of each individual cell type. This accomplishes transcriptional repression of repetitive DNA sequences and allows housekeeping genes to be expressed, but it also provides long‐term stability to the tissue‐specific gene expression profile of somatic cells.

DNA methylation patterns are dynamic states balanced by methylation and demethylation processes. The ‘maintenance methyltransferase’ DNMT1 mainly maintains the methylation patterns across replication cycles while the *de novo* DNMT3A and DNMT3B enzymes mainly introduce methyl groups onto DNA at sites that had been unmethylated (Jurkowska *et al*., 2011).

Regarding DNA demethylation, both passive and active processes have been proposed. Passive demethylation includes everything that negatively affects DNA methyltransferase action, thus leading to the loss of 5mC marks on the genome during DNA replication. However, evidence has been accumulated indicating the existence of replication‐independent active DNA demethylation involving 5‐hydroxymethylcytosine formation and DNA repair mechanisms (Schübeler, 2015).

Several studies have clearly demonstrated that distinct DNA methylation changes highly correlate with aging throughout the entire lifespan of humans and mice. Collectively, these studies have shown that aging, similar to cancer, is associated with gradual but profound changes in DNA methylation where the epigenome is marked by global hypomethylation together with an opposite process of focal hypermethylation preferentially at CGI promoters (Issa, 2014).

These phenomena erode the normal genomic methylation patterns leading to divergent methylomes in the normal population as a function of chronological age. However, directional changes in specific regions in aged individuals occur in the context of age‐associated increase of DNA methylation entropy and this process is commonly defined as ‘epigenetic drift’ (Fraga *et al*., 2005). Although the presence of an aberrant DNA methylation signalling and its outcome in aging progression are well described, the underlying molecular mechanisms are far from being understood.

Transcriptional control of DNA methyltransferases (DNMTs) can be responsible for changes in protein level or enzymatic activity both in physiological and in pathological conditions (Tsai *et al*., 2012; Calabrese *et al*., 2014). Previous studies indicate that aging affects the expression of DNMTs, suggesting this as being one of the mechanisms involved in the deregulation of the methylation patterns observed in aging. However, these observations derive from very few studies analysing relatively small sample sizes (Zhang *et al*., 2002; Casillas *et al*., 2003; Balada *et al*., 2008; Xiao *et al*., 2008; Li *et al*., 2010; Qian & Xu, 2014).

Here, we report results from an European large‐scale cross‐sectional study aimed at investigating the association of DNMTs expression with age as part of the MARK‐AGE project.

MARK‐AGE is a European‐wide population study, supported by the European Commission (FP7), aiming to discover biomarkers of aging which would serve as a reliable measure of biological age (Bürkle *et al*., 2015; Capri *et al*., 2015).

We measured the transcript levels of *DNMT1* and *DNMT3B* genes and analysed their variation with age in peripheral blood mononuclear cells (PBMCs) from more than two thousand age‐stratified donors (35–75 years) from the general population from eight European countries (Capri *et al*., 2015).

Our data show that age has an influence on the expression of *DNMT1* and *DNMT3B* genes in PBMCs. This interaction does not significantly depend on nutritional, lifestyle and clinical variables influencing the expression of DNMTs in the population.

## Results

### Characteristics of the study population

The analysis of the transcript levels of *DNMT1* and *DNMT3B* was carried out on PBMCs from blood samples obtained from donors (2453 individuals) from eight European countries (Table 1).

**Table 1:** Characteristics of the study population by age groups^a^

Age groups	All	1	2	3	4	*P*
Age range (years)	35–75	35–44	45–54	55–64	65–75	
*N*	2453	383	484	807	779	
Age (years)	57.6 ± 10.7	39.6 ± 2.8	49.6 ± 2.8	59.9 ± 2.8	69.0 ± 3.0	< 0.001
Male % (*n*)	47 (1145)	46 (177)	47 (226)	45 (366)	48 (376)	0.707
Smoker, never	52 (1264)	59 (227)	48 (233)	49 (393)	53 (401)	< 0.001
Former	34 (826)	17 (66)	33 (162)	37 (297)	39 (301)	
Current	15 (363)	23 (90)	18 (89)	14 (117)	9 (67)	
Group, GO	18 (437)	0 (1)	3 (17)	25 (205)	28 (214)	< 0.001
RASIG	72 (1774)	99 (381)	93 (449)	60 (483)	59 (461)	
SGO	10 (242)	0 (1)	4 (18)	15 (119)	13 (104)	
BMI (kg/m^2^)	26.3 ± 4.5	24.9 ± 4.6	25.6 ± 4.4	26.7 ± 4.5	27.0 ± 4.3	< 0.001
< 25	43 (1059)	59 (226)	50 (244)	39 (317)	35 (272)	
25 to < 30	39 (957)	28 (109)	35 (171)	41 (331)	44 (346)	< 0.001
≥ 30	18 (436)	12 (48)	14 (69)	20 (159)	21 (160)	
Finland	10 (249)	2 (9)	4 (18)	13 (109)	14 (113)	
Italy	20 (485)	26 (98)	22 (105)	18 (144)	18 (138)	
Austria	11 (267)	18 (69)	14 (69)	9 (69)	8 (60)	
Greece	13 (317)	18 (71)	16 (78)	11 (86)	10 (82)	< 0.001
Poland	11 (270)	12 (46)	11 (52)	12 (99)	9 (73)	
The Netherlands	8 (189)	0 (0)	1 (3)	10 (79)	14 (107)	
Belgium	14 (350)	8 (30)	14 (69)	16 (131)	15 (120)	
Germany	13 (326)	16 (60)	18 (90)	11 (90)	11 (86)	

a Values are mean ± SD and percentage (number), all such variables; one missing case for BMI; *P*‐value: one‐way ANOVA (continuous variables) and chi‐square test (prevalence). Definition of abbreviations is provided in the Data S2.

The largest group of samples consisted of the RASIG (Randomly‐Recruited Age‐Stratified Individuals from the General population), representing individuals undergoing a normal aging process.

Offspring of nonagenarians previously studied in the GEHA study (Genetics of Healthy Ageing; Franceschi *et al*., 2007) and termed GO (‘GEHA offspring’) were also recruited as a potential model of ‘retarded aging’, based on the assumption that their genetic background may predispose them to longevity. As a control for environmental factors and lifestyle, recruitment of GO was accompanied by the recruitment of their spouses, termed SGO (‘spouses of the GO’) (Bürkle *et al*., 2015; Capri *et al*., 2015).

MARK‐AGE subjects covered the age range between 35 and 75 years and were stratified into four 10‐year age groups. The age distribution of RASIG individuals was almost homogeneous, while GO and SGO individuals fell into age ranges > 54 years. The composition of male and female individuals was mainly comparable in all age groups, albeit female representation was a few percentage points larger than males. In agreement with literature data studying subjects in the age range 35–75 years, the body mass index (BMI) appeared to increase with age indicating that the analysed population was effectively representative of a physiological aging process. The characteristics of the study population referred to each recruiting centre are reported in Table S1 (Supporting information).

### Identification of outliers and distribution tests

Due to positively skewed values, *DNMT1* and *DNMT3B* expression data failed to pass the Kolmogorov–Smirnov test for normal distribution. We also identified data of *DNMT1* mRNA (three samples) with values above 8× the interquartile range, which were considered outliers and excluded in all parametric analyses (but included in nonparametric tests, *for example* Spearman correlation and Kruskal–Wallis, which are not sensitive to few outliers). After removal of outliers, the *DNMT1* expression data still failed to pass normality tests. Hence, we analysed Q‐Q plots of the log‐transformed and not‐transformed variables of *DNMT1* and *DNMT3B* to establish the distribution that best represented our data. On the basis of our analysis, the distributions that better represented data were gamma distribution for *DNMT1* and normal distribution for log‐transformed *DNMT3B* data (Fig. S1, Supporting information). However, since the optimal distribution was not identified, we performed both parametric and nonparametric tests to verify the robustness of results.

### Expression levels of *DNMT1* and *DNMT3B* transcripts with respect to age and demographics

In the RASIG samples, we identified nonlinear but significant changes of *DNMT1* in age groups by parametric and nonparametric tests. *DNMT1* transcript decreased gradually below 64 years of age, transiently achieving a significant reduction in the 55–64 vs. the 35–44 age group before returning to levels comparable to the under‐54 in individuals over the age of 65 years. Regarding *DNMT3B*, its expression appeared to decrease linearly with age, with a significant reduction in the two oldest age classes compared to the first one (Table 2). Graphical representation of *DNMT1* and *DNMT3B* data describes better the minimal, but significant age‐related changes observed in the samples. In particular, *DNMT3B* displayed a significant linear decrease with age, while *DNMT1* displayed a U‐shaped bimodal pattern. This U‐shaped bimodal pattern for *DNMT1* mRNA variation with age was less evident when the samples were analysed as a whole (RASIG together with GO and SGO samples) due to a lack of significance for the recovery of transcript levels in the oldest age group compared to younger age groups. Regarding *DNMT3B*, its reduction with increasing age was independent of the inclusion of GO and SGO in the analysis (Fig. S2).

**Table 2:** Effect of age, gender, BMI on *DNMT1* and *DNMT3B* expression in the RASIG population

	Stat	*N*	*DNMT1*			*DNMT3B*		
			Median (IQ)	*P* (KW)^a^	*P* (GLM)^b^	Median (IQ)	*P* (KW)^a^	*P* (GLM)^b^
Age group (years)								
35–44	a	381	0.134 (0.102–0.171)^c^	0.003	0.049	0.022 (0.017–0.029)^c,d^	0.004	0.030
45–54	b	449	0.129 (0.095–0.159)			0.021 (0.016–0.029)		
55–64	c	483	0.117 (0.088–0.165)^a,d^			0.020 (0.015–0.027)^a^		
65–75	d	461	0.129 (0.095–0.171)^c^			0.020 (0.015–0.026)^a^		
Centre								
Finland	a	80	0.125 (0.092–0.154)^b^	< 0.001	< 0.001	0.024 (0.018–0.031)^g^	< 0.001	< 0.001
Italy	b	362	0.144 (0.113–0.183)^a,c,d,g,h^			0.020 (0.015–0.025)^c^		
Austria	c	267	0.113 (0.080–0.165)^b,e^			0.023 (0.015–0.035)^b.g^		
Greece	d	296	0.129 (0.095–0.171)^b,g^			0.021 (0.016–0.031)^g^		
Poland	e	202	0.139 (0.105–0.177)^c,g^			0.022 (0.016–0.028)^g^		
The Netherlands	f	0	–			–		
Belgium	g	241	0.109 (0.077–0.139)^b,d,e,h^			0.017 (0.014–0.024)^a,c,d,e^		
Germany	h	326	0.129 (0.102–0.159)^b,g^			0.020 (0.016–0.027)		
Gender								
F	a	940	0.129 (0.098–0.165)	0.056	0.031	0.021 (0.016–0.029)^b^	0.001	0.002
M	b	834	0.125 (0.092–0.165)			0.020 (0.015–0.027)^a^		
BMI classes								
< 25	a	810	0.125 (0.095–0.165)	0.047	0.377	0.020 (0.015–0.028)	0.078	0.019
25 to < 30	b	662	0.125 (0.092–0.165)			0.021 (0.016–0.027)		
≥ 30	c	301	0.139 (0.102–0.171)			0.022 (0.016–0.028)		

a KW test: nonparametric comparison by the Kruskal–Wallis test of *DNMT1* and *DNMT3B* mRNAs levels (data for two group comparison are analysed with the Mann–Whitney *U*‐test); data are reported as median and interquartile range (IQ). Pairwise comparisons are referred to the KW test and adjusted for multiple comparisons (comparisons with *P* < 0.05 are marked by the associated superscripts).

b GLM: comparison by generalized linear models of *DNMT1* (gamma distribution with log‐link function model) and *DNMT3B* (linear model with log‐transformed values and identity link function) mRNAs levels. For the investigation of age‐group effects, the model included the effects of gender and recruitment centre as factors. All other GLM models included the effects of gender, recruitment centre and age (continuous variable) as covariate (only *P* values for the selected variables are shown). Definition of abbreviations is provided in the Data S2.

**Figure 1:**
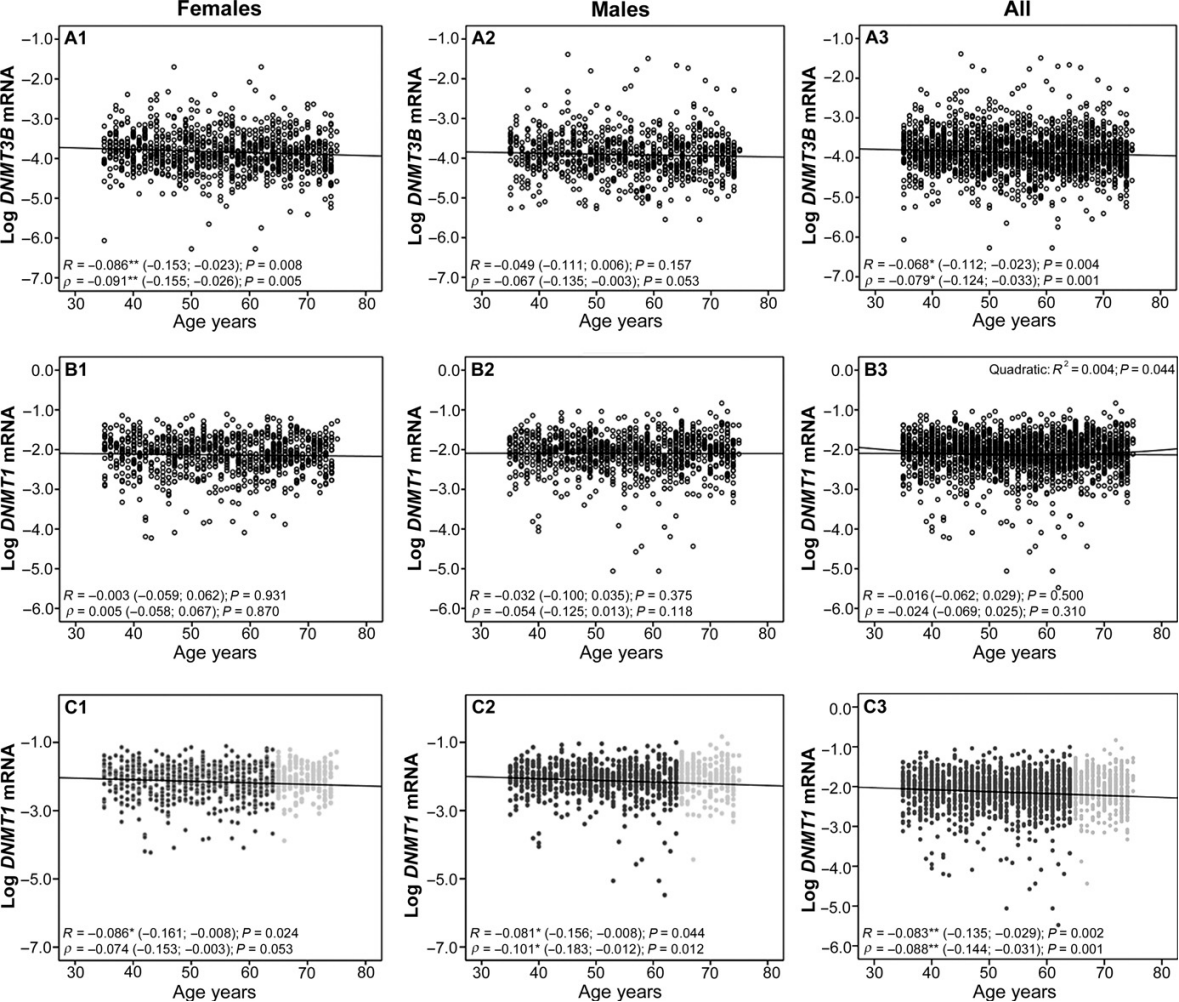
Age‐related changes of *DNMT1* and *DNMT3B* mRNA levels with age in females and males from RASIG population. The picture shows a graphical representation of *DNMT1* and *DNMT3B* mRNA as log‐transformed data vs. age in the RASIG sample. (A1) Dot plot of log‐transformed *DNMT3B* data vs. age in RASIG females; (A2) dot plot of log‐transformed *DNMT3B* data vs. age in RASIG males; (A3) dot plot of log‐transformed *DNMT3B* data vs. age in all RASIG samples; (B1) dot plot of log‐transformed *DNMT1* data vs. age in RASIG females; (B2) dot plot of log‐transformed *DNMT1* data vs. age in RASIG males; (B3) dot plot of log‐transformed *DNMT1* data vs. age in all RASIG samples. Correlation coefficients (Pearson R for log‐transformed data and Spearman's *rho* for untransformed data) are noted below each graph. Significance and 95% confidence interval of correlation coefficients (within brackets) are estimated by bias‐corrected and accelerated (BCa) bootstrap with stratified sampling (1000 samples stratified for country, and also for gender when all data are used). R‐square and significance of the unique relevant quadratic regression is noted above panel B3. Graph and regression coefficients are also reported considering data of *DNMT1* in the age range from 35 to 64 years in RASIG female (panel C1), in RASIG males (panel C2) as well as in all RASIG population (panel C3).

Association between age and DNMTs transcript levels in the RASIG population was further tested by correlation analyses. *DNMT3B* showed a weak negative linear correlation with age. However, this association remained significant only in females when the samples were split based on gender. The same analysis confirmed that the association of *DNMT1* with age was not linear when tested over the entire age range, which is in line with the U‐shaped trend of *DNMT1* variation with age. Consistently, a quadratic relationship between *DNMT1* and age showed a better fit to the data than the linear one, which was, instead, significant and negative for both genders when individuals over the age of 65 years were excluded from the analysis (Fig. 1). The level of expression of both *DNMT1* and *DNMT3B* was dissimilar across the different European countries (Table 2). Moreover, the expression levels across age groups within each recruitment centre were significantly different for part of them probably as a consequence of the high variability, gender‐specific effects and the relative lower sample size of each centre compared to the overall sample (Table S2).

Table 2 also shows that both the *DNMT1* and *DNMT3B* mRNA expression were significantly higher in females compared to males. This association with gender was evident for *DNMT1* only by the generalized linear model (GLM) test that includes age and country as covariates, suggesting these variables as confounding factors in the nonparametric test. The same conclusions can be drawn for the positive association observed between *DNMT3B* expression and BMI.

### Investigation of variables (lifestyle, dietary habits, haematological parameters and cardiovascular risk factors) that potentially affect *DNMT1* and *DNMT3B* mRNA expression

From all subjects enrolled in the MARK‐AGE project, a large number of anthropometric and medical data were collected. In a preliminary analysis, we evaluated variables that might have a potential impact on *DNMT1* and *DNMT3B* expression, including dietary and lifestyle habits, haematological parameters, and cardiovascular or diabetes risk factors. Several variables were found to be associated with DNMTs expression by nonparametric Kruskal–Wallis (KW) test only. In fact, many of these associations were disproved or blunted by GLM which included the effects of gender, recruitment centre and age as covariates (See Tables S3–S5). Such evidence indicates that those associations with DNMTs expression, reported by KW analysis, were indirect. Examples of such discordance include the associations of *DNMT1* expression with French fries and brown bread consumption or with glucose and homocysteine blood levels.

Based on the congruence between KW and GLM tests, a limited set of variables appeared to be consistently associated with the expression of DNMTs. Apart from a link between weekly consumption of white bread and *DNMT1* mRNA levels, no association with other self‐reported dietary habits was detected (Table S3). This also concerns consumption of alcoholic beverages and smoking habits as well as cardiovascular and diabetes risk biomarkers (Table S4). As far as haematological parameters are concerned (Table S5), *DNMT1* and *DNMT3B* mRNAs were less expressed with increasing monocyte counts. Instead, only *DNMT1* was expressed more in samples characterized by an increased number of lymphocytes and of higher lymphocytes/monocytes ratio. This was even more evident when looking at the T lymphocyte (CD3+CD45+) content, which also showed a positive association with *DNMT3B*. *DNMT1* was also less expressed in samples characterized by an increased amount of neutrophils, while *DNMT3B* showed an increasing trend with increasing number of platelets. Of note, the relationship of DNMTs expression levels in PBMCs with lymphocytes and monocytes blood counts mostly resembles the higher expression of these genes in lymphocytes subtypes with respect to monocytes, as revealed by the analysis of publicly available microarray expression data (Su *et al*., 2004) (Fig. S3).

Similar results were obtained by analysing the whole population of samples (RASIG together with GO and SGO samples) (Table S6).

### Contribution of selected variables on age‐related changes of *DNMT1* and *DNMT3B* mRNA expression in the RASIG population

GLM and regression analyses were used to determine whether the age‐related changes observed for *DNMT1* and *DNMT3B* genes are an intrinsic feature of the aging process or whether one or more of the variables shown to influence their expression can justify these changes.

As shown in Table 3, the changes of *DNMT1* expression in age groups seem to depend on a combined impact of multiple parameters rather than on a specific one. However, the age‐related variations of *DNMT1* mRNA levels remained significant even when considering all the variables that significantly affected *DNMT1* expression (i.e. showing a significant interaction with *DNMT1* expression as detected by both KW and GLM tests, see Table 2 and Tables S3–S5), indicating that the analysed parameters cannot fully explain the *DNMT1* transcript differences observed between age groups. To confirm the result, several additional models were run with progressive inclusion of variables categorized on the basis of any significant association with *DNMT1* expression, also including only those identified by KW test (Tables S7A and B). Further analysis using the log‐transformed values of *DNMT1* yielded comparable data (Table S8A and B).

**Table 3:** Influence of selected factors and covariates on age‐related changes of *DNMT1* expression^a^

Variables	Type III		
	Wald chi‐square	df	Sig.
Age groups	8.760	3	0.033
Gender	0.826	1	0.363
Centre	53.167	6	< 0.001
White bread consumption	6.877	2	0.032
Monocytes	0.123	1	0.726
Ratio lymphocyte to monocyte	19.989	1	< 0.001
Neutrophils	0.240	1	0.624

Model: age groups (*o*), gender (*n*), centre (*n*), white bread consumption (*s*), monocytes (*s*), lymphocytes/monocytes (*s*), neutrophils (*s*), (*o* = ordinal variable: *n* = nominal variable; *s* = scale variable).

a Analysis was performed by GLM using gamma distribution with log‐link function and nontransformed data of the dependent variable: *DNMT1* mRNA (*s*).

Similar results concerned the age‐related variation of *DNMT3B* transcript. Its linear decrease with age (Table 2 and Fig. 1) allowed the investigation by regression analysis. Taking into account the gender difference identified in Fig. 1, we performed a separate analysis for gender. The analysis confirmed the linear decrease of *DNMT3B* mRNA levels in females (but not males). The linear decrease of *DNMT3B* in the females was not affected by the inclusion of other variables associated with differences in *DNMT3B* mRNA levels between individuals (Table 4).

**Table 4:** Regression analysis of *DNMT3B* expression in females and males from RASIG^a^

	Gender	Variables	Coefficients		Bootstrap for coefficients		
			B ± SE	Beta	Bias	Sig	95% CI
Model 1	F	Age (years)	−0.004 ± 0.001	−0.086	0.000	0.008	−0.007; −0.001
	M	Age (years)	−0.003 ± 0.002	−0.059	0.000	0.077	−0.006; 0.000
Model 2	F	Age (years)	−0.004 ± 0.001	−0.084	0.000	0.009	−0.007; −0.001
		Lymphocytes/monocytes	0.293 ± 0.100	0.087	−0.004	0.005	0.083; 0.466
	M	Age (years)	−0.002 ± 0.002	−0.042	0.000	0.310	−0.005; 0.001
		Lymphocytes/monocytes	0.190 ± 0.121	0.056	0.001	0.087	−0.035; 0.408
Model 3	F	Age (years)	−0.006 ± 0.002	−0.128	0.000	0.001	−0.009; −0.002
		Lymphocytes/monocytes	0.271 ± 0.131	0.079	0.011	0.021	0.041; 0.525
		BMI	0.481 ± 0.111	0.167	−0.007	0.001	0.280; 0.671
		HDL	0.148 ± 0.073	0.075	−0.003	0.047	0.019; 0.278
		Monocytes	−0.045 ± 0.036	−0.048	−0.001	0.068	−0.102; 0.006
		MHC	−0.140 ± 0.361	−0.013	−0.176	0.688	−1.417; 0.238
		Platelets	−0.065 ± 0.052	−0.044	0.013	0.385	−0.173; 0.133
		HGB	−0.062 ± 0.234	−0.009	0.018	0.801	−0.527; 0.445
	M	Age (years)	−0.002 ± 0.002	−0.038	0.000	0.302	−0.005; 0.002
		Lymphocytes/monocytes	0.223 ± 0.152	0.062	0.007	0.145	−0.092; 0.524
		BMI	0.206 ± 0.163	0.052	0.005	0.161	−0.077; 0.507
		HDL	0.038 ± 0.088	0.018	0.001	0.690	−0.127; 0.222
		Monocytes	−0.015 ± 0.048	−0.013	−0.001	0.685	−0.083; 0.065
		MHC	−0.128 ± 0.190	−0.025	−0.250	0.359	−1.594; −0.026
		Platelets	0.087 ± 0.059	0.055	0.000	0.109	−0.037; 0.215
		HGB	−0.169 ± 0.262	−0.024	0.021	0.482	−0.745; 0.407

a Regression analysis was performed by using log‐transformed data of dependent and independent variables (with the exclusion of age). All data were included as continuous variables. Bootstrap results are based on 1000 stratified (by recruitment centre) bootstrap samples. Definition of abbreviations is provided in the Data S2.

### Identification of major variables affecting the measurement of *DNMT1* and *DNMT3B* expression

Decision tree analysis including all variables with at least one significant effect on *DNMT1* and *DNMT3B* (see Table 2 and Tables S3–S5) was performed to identify the major factors affecting DNMTs expression. We identified the recruitment centre and the lymphocyte‐to‐monocyte ratio as being the most important factors that affect both *DNMT1* (Fig. S4) and *DNMT3B* (Fig. S5). Minor subgroups were identified on the basis of age, WBC and CD3+CD45+ cell data.

### Differences in *DNMT1* mRNA expression between GO, SGO and RASIG

**Figure 2:**
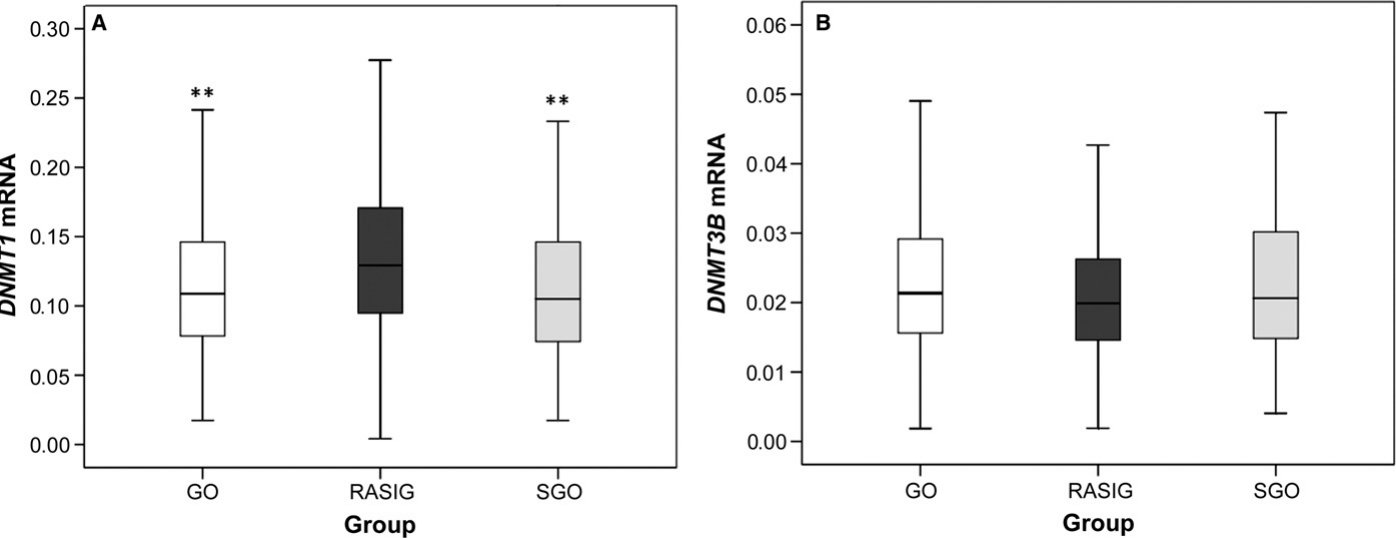
Levels of *DNMT1* and *DNMT3B* mRNA in GO, SGO and RASIG. The picture shows a graphical representation of *DNMT1* (A) and *DNMT3B* (B) mRNA level in PBMCs from GO, SGO and RASIG from the whole MARK‐AGE sample above 54 years. Analysis was performed in subjects above 54 years due to nonrepresentative numbers of GO and SGO below this age. **P < 0.01 from RASIG by *post hoc* (LSD) of GLM analysis and by KW test performed within each country.

To investigate the possible influence of genetic advantage expected for GO samples, the analysis of *DNMT1* and *DNMT3B* expression was performed comparing samples with an age between 54 and 75 years as GO and SGO were well represented in this age interval. The stratification by 10‐year age groups of subjects aged > 54 years led to a similar division of GO, SGO and RASIG across the age groups, thus reducing potential bias due to unequal divisions. As shown in Fig. 2A, the expression of *DNMT1* was significantly higher in RASIG compared to GO and SGO, while no difference was observed between GO and SGO. The comparison of *DNMT1* expression between age‐stratified GO, SGO and RASIG samples was performed according to gender and recruitment centre. Data show that the higher level of *DNMT1* mRNA in RASIG vs. GO and SGO seemed to be a common feature, although the statistical significance was reached in some age/centre subgroups, likely due to reduced sample size (Table S9). In contrast, the expression levels of *DNMT3B* were very similar in the three classes of samples (Fig. 2B and Table S10).

With the aim to trace potential causes for the differences in *DNMT1* mRNA expression between GO, SGO and RASIG, the contribution of selected variables was assessed by GLM analysis (Table 5). Results show that the differences between the three classes of samples remained highly significant even when several variables that associated with interindividual variations in *DNMT1* mRNA expression were taken into account. Additional models that confirmed these results were also run including all categorized variables associated with *DNMT1* in Tables S3–S5 (Table S11A and 11B).

**Table 5:** Contribution of selected variables and covariates on group (GO, RASIG and SGO) related changes of *DNMT1* expression in PBMCs from population aged > 54 years^a^

Tests of model effects			
Source	Type III		
	Wald chi‐square	df	Sig.
Group (GO, SGO, RASIG)	15.707	2	< 0.001
Recruitment centre	78.056	7	< 0.001
Gender	0.041	1	0.839
Age (years)	4.107	1	0.043
BMI	2.565	1	0.109
Serum glucose	0.079	1	0.778
Glycosylated haemoglobin A1C	0.015	1	0.903
Homocysteine	4.236	1	0.040
Neutrophils	1.372	1	0.241
Lymphocytes/monocytes	19.814	1	< 0.001
Monocytes	0.538	1	0.463
WBC	1.179	1	0.278
HCT	0.925	1	0.336
MCV	0.635	1	0.426
MCH	0.064	1	0.801

a Analysis was performed by GLM using gamma distribution with log‐link function. Dependent variable: *DNMT1* mRNA. Model: group (*n*), recruitment centre (*n*), gender (*n*), age (s), BMI (*s*), serum glucose (*s*), glycosylated haemoglobin A1C (*s*), homocysteine (*s*), neutrophils (*s*), lymphocytes/monocytes (*s*), monocytes (*s*), WBC (*s*), HCT (*s*), MCV (*s*), MCH (*s*); (*n* = nominal variable; *s* = scale variable). Definition of abbreviations is provided in the Data S2.

### The effect of batch correction strategies on the association of *DNMT1* and *DNMT3B* mRNA expression with age and on differences in *DNMT1* mRNA expression between GO, SGO and RASIG

Our experimental design has been conceived to minimize the impact of batch effects by the adoption of a validated housekeeping gene for normalization of expression data in aging (Zampieri *et al*., 2010) as well as the use of a calibrator sample to reduce inter‐run variation. Clearly, the presence of additional batch effects could not be completely ruled out though. In an attempt to address this problem, the age‐related changes in RASIG and the differences between GO, SGO and RASIG of DNMTs expression were tested against diverse procedures of batch effects adjustment by the application of a dedicated software. The following strategies were adopted: (i) simple correction of batch effects; (ii) correction attempting to retain differences between groups (GO, SGO and RASIG), age and gender; (iii) group‐, age‐ and gender‐sensitive correction combined with filtering for eventual outliers due to overcorrection. As shown in the Fig. S6, the application of the correction procedures gradually normalized the distribution of data of both DNMTs. Concerning *DNMT1*, correction procedures uncovered a weak albeit significant linear decline with age (Fig. S7). The stratification of data for age and gender revealed that adjustment for batches mainly affected the upregulation of *DNMT1* in the last age class obtained with unadjusted data, while a decreasing trend characterized both uncorrected and corrected data in age groups up to 64 years (Fig. S8). Consistently, a significant negative linear correlation of *DNMT1* expression with age was found in the samples up to 64 years independently of correction for batches (Fig. S9). The batch correction procedure had no relevant impact on the differences between GO, SGO and RASIG groups even when considering the major confounding factors in the analysis (Fig. S10). As *DNMT3B* concerns, batch correction confirmed and strengthened the decrement of expression with age (Figs S11 and S12).

## Discussion

The mechanisms responsible for the coexistence of global hypomethylation and hypermethylation of specific sequences in the aging genome are still an open research field. In this context, the altered expression of DNMTs has long been postulated to contribute to epigenetic instability in aging. This possibility was mainly prompted by the fact that changes of DNA methylation patterns and of DNMTs expression are well documented in aging‐associated diseases such as cancer, autoimmune diseases and Alzheimer's disease.

The results of pioneering studies, carried out on cellular models of aging, have shown a substantial change of DNMTs activity with increasing age due to a combination of maintenance methylation deficit and increased *de novo* methylation (Lopatina *et al*., 2002), events which were then associated with reduced DNMT1 levels together with increased DNMT3B, respectively (Casillas *et al*., 2003). In part, these initial observations have been further supported by the results of *in vivo* studies in humans and mice. In fact, the levels of *DNMT1* expression decrease with age in human T lymphocytes and this is associated with hypomethylation of specific gene promoters (Zhang *et al*., 2002; Balada *et al*., 2008; Li *et al*., 2010). By contrast, data on changes of *DNMT3B* expression during aging are less concordant since, while its increase was confirmed in the liver of aged humans (Xiao *et al*., 2008), a decrease with age was observed in human T lymphocytes (Balada *et al*., 2008) as well as in mouse skin (Qian & Xu, 2014). Collectively, these data indicate that a transcriptional deregulation of DNMTs would probably accompany the aging process. However, the available data on humans are derived from a limited number of correlational studies generally carried out on relatively small sample sizes.

In the present work, the possible relationship between aging and expression of *DNMT1* and *DNMT3B* has been tested in the context of a large‐scale population‐based study thereby providing, for the first time, a reference framework for factors that are associated with DNMTs expression variation such as demographics, clinical laboratory parameters as well as dietary and health habits. Using PBMCs, one of the few accessible tissues, we broadly assessed variation of *DNMT1* and *DNMT3B* transcript levels in more than two thousand individuals recruited across the European population covering the age range of 35–75 years.

Although no substantial correlation was observed between *DNMT1* expression and age by linear regression analysis, a significant, but very small variation of the expression of both transcripts, was found when samples were stratified into 10‐year age groups.

In agreement with previous reports (Zhang *et al*., 2002; Balada *et al*., 2008; Li *et al*., 2010), the level of *DNMT1* gradually dropped with aging. However, this was observed up to 64 years, where it either appeared to stabilize (batch corrected data) or to eventually raise again (uncorrected data) afterwards. Anyway, the U‐shaped pattern of the uncorrected *DNMT1* expression data with age in humans is also supported by observations in a recent publication, where the same trend was observed for the expression of *DNMT1* as well as for the global methylation level of the genome in mouse liver during aging (Armstrong *et al*., 2013). Interestingly, the decrease with age of the methylation levels of repetitive DNA sequences is not linear in humans either, but it mainly occurs during ages 40–60 years (Jintaridth & Mutirangura, 2010).

Concerning *DNMT3B*, its transcript levels decreased with age in agreement with previous observations in human T lymphocytes (Balada *et al*., 2008). Looking at gender, the inverse correlation of *DNMT3B* expression with age was significant in the female population.

Notably, the changes with age of both DNMTs within each recruitment centre were not uniform. While this could be in part attributed to the reduced sample size, a differential impact of environmental variables on the relationship between expression of DNMTs and age across different countries cannot be excluded. Significantly, environmental variables have been described to have an impact on genomic DNA methylation patterns as well as on DNMTs expression level (see Zampieri *et al*., 2015 for review).

We next performed an analysis to examine the association of DNMTs expression with a large set of variables including demographics, clinical laboratory parameters, dietary and lifestyle habits.

Concerning demographic variables, an interaction with the expression of DNMTs was found for gender and BMI in addition to age and country.

Gender differences in the expression of both DNMTs in PBMCs are in line with data previously obtained in the liver where higher levels of both *DNMT1* and *DNMT3B* were observed in females (Xiao *et al*., 2008). Furthermore, gender‐related differences of DNA methylation patterns in PBMCs have recently been described (Lam *et al*., 2012). However, our data suggest that gender differences for *DNMT1* are the consequences of a different proportion in leucocytes subsets.

The positive association of *DNMT3B* expression with BMI is of great interest. In fact, an association between BMI and epigenetic age acceleration was recently observed, especially in liver (Horvath *et al*., 2014). In this context, our results would suggest an interesting link between obesity, altered *DNMT3B* expression and methylation defects that predispose to disease. Consistently, enhanced *DNMT3B* expression was proposed to contribute to deregulated adipose tissue macrophage polarization, inflammation and insulin resistance in obesity (Yang *et al*., 2014).

Surprisingly, no significant association between dietary habits and level of DNMTs transcripts was found although increasing evidence indicates DNA methylation is vulnerable to nutritional influences (Bacalini *et al*., 2014). This was also the case of clinical chemistry parameters associated with cardiovascular and diabetes risk. For some of them, association with DNMTs expression was found to be indirect and partially explained by other demographic factors including, but not exclusively, age.

The lack of association between smoking and level of DNMTs was unexpected considering that cigarette smoking is one of the most powerful environmental modifiers of the DNA methylation pattern (Breitling *et al*., 2011; Lee & Pausova, 2013) and has been shown to deregulate the expression of DNMTs in brain and lung (Satta *et al*., 2008; Lin *et al*., 2010). This discrepancy could be explained by the fact that this effect could be tissue‐specific and does not concern the PBMCs. In fact, a lack of statistical association was reported between smoking and genomewide DNA methylation variation in PBMCs (Lam *et al*., 2012) as well as between smoking and epigenetic age acceleration in multiple tissues (Horvath *et al*., 2014).

Finally, the expression of DNMTs was influenced by the amount of lymphocytes, monocytes and by a specific subset of T lymphocytes. This points to the composition of PBMCs as a confounding factor that may lead to differences in expression of DNMTs between individuals. Hence, the study of the relationship between abnormal methylation machinery and DNA methylation changes in blood cells should take into account differences in leucocyte composition between individuals. In particular, the lymphocyte‐to‐monocyte ratio emerged as the main variable affecting *DNMT1* and *DNMT3B*. These data also suggest that DNMTs might be more expressed in lymphocytes than monocytes. Interestingly, it seems to be the case according to the comparison between blood cell types of *DNMT1* and *DNMT3B* transcript levels obtained from published microarray data.

After the identification of variables potentially involved in determining variations in the expression of DNMTs in our population, we then sought to determine the impact of these variables on the differences in the expression of DNMTs between age groups. The results indicate that, although all the critical variables were taken into account in the analysis, the differences in the expression of both *DNMT1* and *DNMT3B* between the age groups are still significant (excluding *DNMT3B* in the male RASIG population). This indicates that age affects the expression of DNMTs in PBMCs as an almost independent variable with respect to all other variables evaluated here. Nevertheless, factors such as geographical origin of the samples and lymphocytes‐to‐monocytes ratio seemed to have a greater influence on interindividual differences in DNMTs expression with respect to age in PBMCs. On the other hand, the weak association of DNMTs expression with age severely limits the possibility of predicting age by measuring DNMTs transcripts in PBMCs in contrast to that shown for the methylation *status* of specific genomic *loci* (Horvath, 2013).

The relationship between DNMTs expression and aging was also evaluated in the GO and SGO populations. GO represents a group of individuals that is assumed to have genetic benefits for healthy aging compared to the normal aging population (RASIG) and to their control of environment and lifestyle (SGO). In this experimental setting, inherent advantages of GO with respect to aging seem not to be related to DNMTs expression. In fact, *DNMT3B* levels were equal between the three groups while the expression of *DNMT1* is even lower in GO with respect to RASIG. Moreover, GO and SGO showed comparable expression of *DNMT1*. This rules out the possibility that the differences in *DNMT1* expression between GO and RASIG are due to genetics while it points to the existence of environmental or lifestyle factors that distinguish GO and SGO from RASIG. However, differences in expression of *DNMT1* between GO, RASIG and SGO do not seem to depend on any of the variables which we found to be associated with the variation of its expression in the analysed population. This suggests that GO and SGO share a similar environmental factor whose influence on the expression of *DNMT1* cannot be traced, at least in any of the parameters analysed here. A speculative hypothesis could be that the point of contact between GO and SGO is their microbiome (including bacteria, fungi and viruses that colonize our organism). In fact, there is evidence that cohabitation leads to microbiota similarities between individuals (Song *et al*., 2013) and that the centenarians have a very different microbiota composition from the rest of the population (Biagi *et al*., 2010; Rampelli *et al*., 2013). Notably, differences in intestinal microbiota between individuals have recently been associated with differences in the pattern of DNA methylation in the blood (Kumar *et al*., 2014).

Finally, given that the most significant age‐related results of this paper originated from very small effect sizes, we next sought to determine whether the aging effect on DNMTs expression was robust to batch effects. It is expected that batch effects would lead to increased variability and decreased power to detect a real biological signal. In fact, in many cases, batch effects increase data variability and can be confused with an outcome of interest thus leading to misleading biological conclusions. This claims the necessity to perform dedicated adjustments especially in population studies that require large sample sizes and have to be carried over a long time period, as in the case of this study. However, the application of batch removal tools to our data, which consisted of heterogeneous (for gender, group and country) small batches, was likely to introduce additional problems. In fact, in the case of study groups being nonevenly distributed across batches, algorithms to remove batch effects may bias group differences, but also carry the potential concern of removing intragroup biological heterogeneity. In order to avoid the pitfall deriving from inappropriate batch correction, diverse approaches have been applied. All adopted procedures confirmed the relationship between expression of DNMTs and age. However, batch correction impacted both the pattern and the strength of this association. The linear decline of *DNMT3B* with age was confirmed and reinforced, as well as the differences in *DNMT1* expression between GO, SGO and RASIG, although with a lower statistical significance. Conversely, removal of batch effects confirmed the decline of *DNMT1* expression up to 65 years while blunted its upregulation in the last age class obtained with unadjusted data, thus disproving the U‐shaped pattern. However, batch correction appeared to introduce a possible bias as shown by the preferential impact on the expression levels of *DNMT1* in the 65–75 age class as well as by the inversion of the mean values between male and females in the same age class the for both DNMTs. This suggests that the results after correction should be interpreted with caution.

Collectively, results from this study confirm in large‐scale population setting that aging has an impact on the expression of DNMTs. Converging evidence is given for the linear decrease of *DNMT3B* expression with age as well as for the *DNMT1* up to 64 years. For higher age, data on the trend of *DNMT1* were conflicting and a possible reading could be that the decrease of *DNMT1* at these ages is attenuated. These data would form the basis for future investigations aimed at establishing if these changes are causally linked to variation in DNA methylation patters and participate to the mechanism of DNA methylation changes during aging.

## Experimental Procedures

### Study population, recruitment, data and blood collection

MARK‐AGE is a European‐wide cross‐sectional population study aimed at the identification of biomarkers of aging (Bürkle *et al*., 2015; Capri *et al*., 2015).

In the present work, the expression of *DNMT1* and *DNMT3B* transcripts was analysed in PBMCs samples from a total of 2453 donors in the age range of 35–75 years recruited in eight different European countries.

Details of the recruitment procedures and of the collection of anthropometric, clinical and demographic data have been published (Moreno‐Villanueva *et al*., 2015a,b).

PBMCs isolation procedure has been described (Moreno‐Villanueva *et al*., 2015a). Briefly, PBMCs were isolated from EDTA‐whole blood, obtained by phlebotomy after overnight fasting, by discontinuous density gradient centrifugation in Percoll and subsequently cryopreserved and stored in liquid nitrogen.

Samples were then shipped from the various recruitment centres to the MARK‐AGE Biobank located at the University of Hohenheim, Stuttgart, Germany. From the Biobank, coded samples were subsequently sent to the Sapienza University of Rome on dry‐ice where they were stored in liquid nitrogen until analysis of the *DNMT1* and *DNMT3B* mRNA levels.

### RNA extraction and cDNA synthesis

Samples were thawed by incubation at 37°C, followed by drop wise addition of RPMI containing 10% FCS to a final dilution of 1:20. Cells were collected by centrifugation and processed for RNA extraction. Isolation of total RNA was performed using RNeasy Mini Kit (Qiagen, Hilden, Germany) according to the manufacturer's instructions and subjected to DNase I digestion using RNase‐free DNase (Qiagen, Hilden, Germany). RNA concentration, purity and integrity were evaluated as previously described (Zampieri *et al*., 2010). Reverse transcription was carried out using the SuperScript VILO cDNA Synthesis Kit (Invitrogen, MA, USA) on equal amounts of total RNA (0.5 μg).

### Real‐time quantitative RT–PCR

The expression of *DNMT1* and *DNMT3B* was determined by quantitative PCR using the Taqman Gene Expression Assays (Applied Biosystems, CA, USA) following the manufacturer's protocol on the iCycler IQ detection system (Bio‐Rad, Hercules, CA, USA). The PCR efficiency for each gene assay was tested using twofold serial dilutions (from 50 to 3.125 ng) of cDNAs randomly chosen from among the samples. Each set of primers and probe showed an efficiency of 90–100%. All calibration curves exhibited correlation coefficients > 0.99. Assays were performed in triplicate with cDNA equivalent to 30 ng of reverse transcribed RNA. Gene expression analysis was performed by the relative calibrator normalized quantification method using the expression level of the β‐glucuronidase gene (*GUSB*) as reference (Zampieri *et al*., 2010). An inter‐run calibration sample was used in all plates to correct for the technical variance between the different runs and to compare results from different plates. The calibrator consisted of cDNA prepared from HCT116 cells. The Taqman Gene Expression Assays IDs for each set of primers and probe were as follows: Hs00154749_m1 (*DNMT1*), Hs00171876_m1 (*DNMT3B*), Hs99999908_m1 (*GUSB*). The data obtained were uploaded to the MARK‐AGE database (Moreno‐Villanueva *et al*., 2015b), established at the University of Konstanz (Konstanz, Germany), where they were recorded, curated and merged with the anthropometric, clinical and demographic data elements of donors.

### Statistical analysis

See supporting information.

## Conflict of interest

None declared.

## Funding info

Financial support by the European Union's Seventh Framework Program, grant no. HEALTH‐F4‐2008‐200880 MARK‐AGE, is gratefully acknowledged.

## Author contributions

PC and MZ designed the research; FC, RC, TG, MGB, AR, MZ, EJ, MD carried out laboratory analyses; BGL and BK recruited participants from Austria, OT Belgium, JB and CS Germany, PES the Netherlands, ESG Greece, CF and MC Italy, ES Poland, AH and MH Finland; NB and TG managed the Biobank; FC, MM, AB, MZ and PC analysed and discussed data; MM performed statistical analysis; MMV and TS were involved in database management; FC, MM, MZ and PC wrote the manuscript; AB coordinated the MARK‐AGE project and edited the manuscript.

## Supporting information


**Fig. S1.** Q‐Q plots tested for different distribution with transformed and not transformed values of *DNMT1* and *DNMT3B* expression.
**Fig. S2.** Age‐related changes of *DNMT1* and *DNMT3B* mRNA levels with age in RASIG and in the whole population.
**Fig. S3.** Transcrip levels of *DNMT1* and *DNMT3B* in the different cell population present in PBMCs.
**Fig. S4.** Identification of major variables affecting *DNMT1* mRNA levels by decision tree analysis.
**Fig. S5.** Identification of major variables affecting *DNMT3B* mRNA levels by decision tree analysis.
**Fig. S6.** Impact of batch effect correction on normal Q‐Q plots of *DNMT1* (upper panels) and *DNMT3B* (lower panels) expression.
**Fig. S7.** Impact of batch effect correction on the age‐related changes of *DNMT1* expression in the RASIG population.
**Fig. S8.** Impact of batch effect correction on the age‐related changes of *DNMT1* expression in the RASIG population displayed as error bar and stratified for gender (green circles = males; blue circles = females).
**Fig. S9.** Impact of batch effect correction on the slope of *DNMT1* expression in the 35–64 and 55–75 age ranges.
**Fig. S10**. Impact of batch effect correction on group‐related changes of *DNMT1* expression.
**Fig. S11**. Impact of batch effect correction on the age‐related changes of *DNMT3B* expression in the RASIG population.
**Fig. S12.** Impact of batch effect correction on the age‐related changes of *DNMT3B* expression in the RASIG population displayed as error bar and stratified for gender.
**Table S1.** Characteristics of the study population by age groups in each recruitment centre.
**Table S2**. Effect of age, gender, BMI on *DNMT1* and *DNMT3B* expression in the RASIG population of each recruitment centre.
**Table S3.** Influence of dietary habits on *DNMT1* and *DNMT3B* expression in the RASIG population.
**Table S4.** Influence of cardiovascular and diabetes risk biomarkers on *DNMT1* and *DNMT3B* expression in the RASIG population.
**Table S5.** Influence of haematological parameters on *DNMT1* and *DNMT3B* expression in the RASIG population.
**Table S6.** Effect of Age, group, demographic characteristics, dietary habits, cardiovascular risk factors and haematological parameters on *DNMT1* and *DNMT3B* expression in the whole population.
**Table S7.** Contribution of selected variables on age‐related changes of *DNMT1* expression in the RASIG population.
**Table S8.** Contribution of selected variables on age‐related changes of *DNMT1* expression in the RASIG population.
**Table S9.** Stratified (gender and country) comparison of *DNMT1* expression among GO, SGO and RASIG.
**Table S10.** Stratified (gender and country) comparison of *DNMT3B* expression among GO, SGO and RASIG.
**Table S11**. Contribution of selected variables on group (GO, RASIG and SGO) related changes of *DNMT1* expression in population aged > 54 years.
**Data S1.** Experimental Procedures.
**Data S2.** List of abbreviations and their definition.
