# Supplementary material for: Age‐dependent expression of DNMT1 and DNMT3B in PBMCs from a large European population enrolled in the MARK‐AGE study
Source: Aging Cell. 2016 May 11;15(4):755–65. doi: 10.1111/acel.12485 (PMC4933658; doi:10.1111/acel.12485)
Supplement: Supplementary file 1 — Fig. S1. Q‐Q plots tested for different distribution with transformed and not transformed values of DNMT1 and DNMT3B expression. Fig. S2. Age‐related changes of DNMT1 and DNMT3B mRNA levels with age in RASIG and in the whole population. Fig. S3. Transcrip levels of DNMT1 and DNMT3B in the different cell population present in PBMCs. Fig. S4. Identification of major variables affecting DNMT1 mRNA levels by decision tree analysis. Fig. S5. Identification of major variables affecting DNMT3B mRNA levels by decision tree analysis. Fig. S6. Impact of batch effect correction on normal Q‐Q plots of DNMT1 (upper panels) and DNMT3B (lower panels) expression. Fig. S7. Impact of batch effect correction on the age‐related changes of DNMT1 expression in the RASIG population. Fig. S8. Impact of batch effect correction on the age‐related changes of DNMT1 expression in the RASIG population displayed as error bar and stratified for gender (green circles = males; blue circles = females). Fig. S9. Impact of batch effect correction on the slope of DNMT1 expression in the 35–64 and 55–75 age ranges. Fig. S10. Impact of batch effect correction on group‐related changes of DNMT1 expression. Fig. S11. Impact of batch effect correction on the age‐related changes of DNMT3B expression in the RASIG population. Fig. S12. Impact of batch effect correction on the age‐related changes of DNMT3B expression in the RASIG population displayed as error bar and stratified for gender. Table S1. Characteristics of the study population by age groups in each recruitment centre. Table S2. Effect of age, gender, BMI on DNMT1 and DNMT3B expression in the RASIG population of each recruitment centre. Table S3. Influence of dietary habits on DNMT1 and DNMT3B expression in the RASIG population. Table S4. Influence of cardiovascular and diabetes risk biomarkers on DNMT1 and DNMT3B expression in the RASIG population. Table S5. Influence of haematological parameters on DNMT1 and DNMT3B expression in the RASIG population. [file ACEL-15-755-s001.docx]

**Supplementary Experimental Procedures**

**Statistical Analysis**

Characteristics of the population studied were described using means and SD for continuous variables (age, BMI) and frequencies (%) for categorical variables (gender, BMI classes, smoking *status*, groups, country). Differences in characteristics between age groups and food habits were compared by one-way-ANOVA (continuous variables) or chi-square test (prevalence).

Distribution of *DNMT1* and *DNMT3B* variables was investigated by running Q-Q plots and by the Kolmogorov Smirnov test. Identification of potential critical variables that can affect *DNMT1* and *DNMT3B* expression in PBMCs was performed by non-parametric tests (Kruskal-Wallis test or Mann-Whitney U test for two group comparisons) as well as by Generalized Linear Models (GLM) adapted to the best distribution that represented the target variables. For this analysis, continuous variables (glucose, glycosylated haemoglobin A1C, triglycerides, LDL, HDL, free fatty acids, homocysteine, white blood cells count, monocyte count, lymphocyte count, lymphocyte to monocyte ratio, CD3+CD45+ cells) were categorized in tertiles or quartiles or considered as continuous variables when indicated. Data of *DNMT1* and *DNMT3B* mRNA expression were reported in all tables as median and interquartile range (IQ). Pairwise comparisons (adjusted for multiple comparisons by Dunn's and Bonferroni’s methods for the Kruskal-Wallis and the GLM tests, respectively) were used to identify significant differences between percentile groups of each categorized variables.

Influence of age, and the relative impact of confounding variables on *DNMT3B,* was investigated by linear regression using log-transformed variables and non-parametric (Spearman) correlations. Correlations were additionally investigated by stratified bootstrap sampling (1000 bootstrap samples). GLM were used to investigate the influence of confounding variables (tested as categorized and continuous variables) on age-related changes of *DNMT1*. The Bonferroni adjustment for multiple comparisons was used to identify differences between subgroups.

The identification of the major variables affecting *DNMT1* and *DNMT3B* mRNAs in PBMCs was performed using decision tree analysis with exhaustive "Chi-squared Automatic Interaction Detector" (CHAID) algorithms. All variables that significantly affected *DNMT1* and *DNMT3B* by parametric or non-parametric tests were included in the decision tree analysis. To determine the best split at any node, the CHAID algorithm chooses the predictor variable with the smallest adjusted p-value, i.e., the predictor variable that will yield the most significant split; if the smallest (Bonferroni) adjusted p-value for any predictor is greater than some alpha-to-split value, then no further splits are performed, and the respective node is a terminal node. The process repeats recursively until one of the stopping rules is triggered. In growing the tree the following stopping rules were used: minimum terminal parental node size of 100 cases, minimum terminal child node size of 50 cases and alpha = 0.05 for splitting nodes. The convergence criteria for the Exhaustive CHAID were: epsilon = 0.001 and 100 as the maximum number of iterations before stopping the process.

Influence of groups (GO, SGO and RASIG), and the relative impact of each factor on *DNMT1* mRNA expression data and their interactions were investigated also running several generalized linear models using non transformed (testing gamma with log-link or normal with log-link distributions) and log-transformed data (testing normal distribution) of *DNMT1* mRNA expression.

The potential influence of batch effects on the solidity of data was investigated by correcting batch effects with the Partek Genomic Suite 6.6 implemented with the ANOVA tool (Partek Incorporated) using log-transformed variables of *DNMT1* and *DNMT3B*. We considered each real-time PCR plate (containing 8 samples with study groups not evenly distributed across plates) as a batch. We performed adjusted for batches by applying the following strategies: i) simple removal of batch effect (including PCR plates as batches in the ANOVA tool; ii) removal of batch effects attempting to retain differences in group, age and gender (identified as variable of interest in the ANOVA tool); iii) application of the strategy specified above combined with a filter for outliers eventually generated by overcorrection based on Z scores (samples whit a Z score +/- 3 were considered outliers).

All statistical analysis (excluding analysis of batch effects) was carried out using SPSS software (SPSS Inc., Chicago, IL; Version 22.0).

**
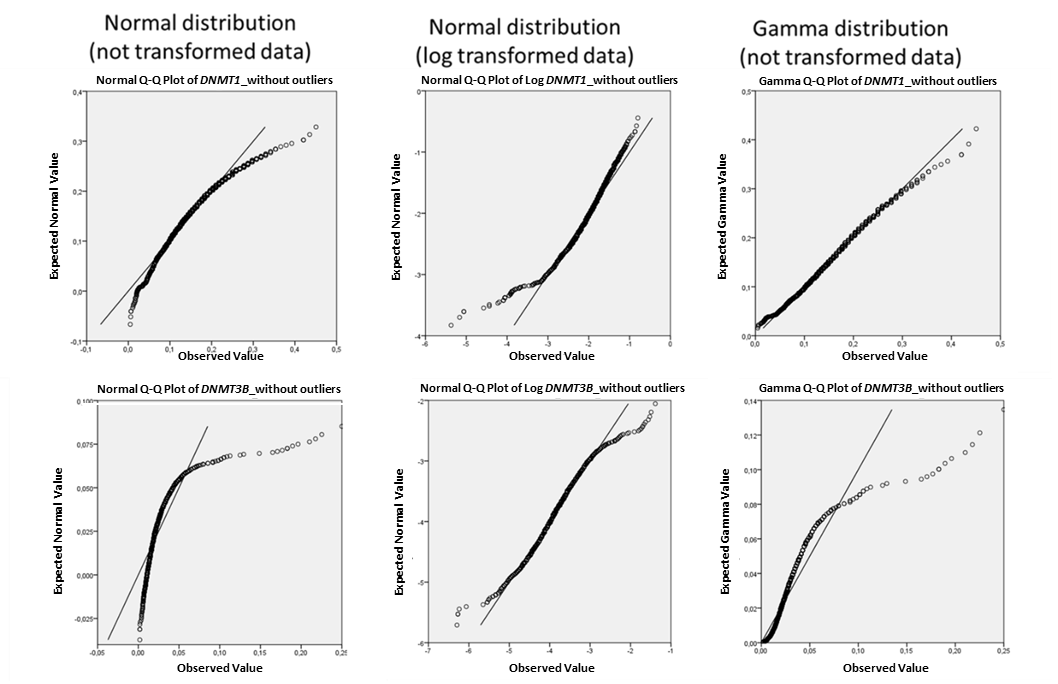
**

**Supplementary Figure 1. Q-Q plots tested for different distribution with transformed and not transformed values of *DNMT1* and *DNMT3B* expression**. Upper 3 panels from left to right: 1) Q-Q plots of *DNMT1* data tested for normal distribution; 2) log-transformed data of *DNMT1* tested for normal distribution; 3) *DNMT1* data tested for Gamma distribution; Lower 3 panels from left to right: 1) Q-Q plots of *DNMT3B* data tested for normal distribution; 2) log-transformed data of *DNMT3B* tested for normal distribution; 3) *DNMT3B* data tested for Gamma distribution.

**
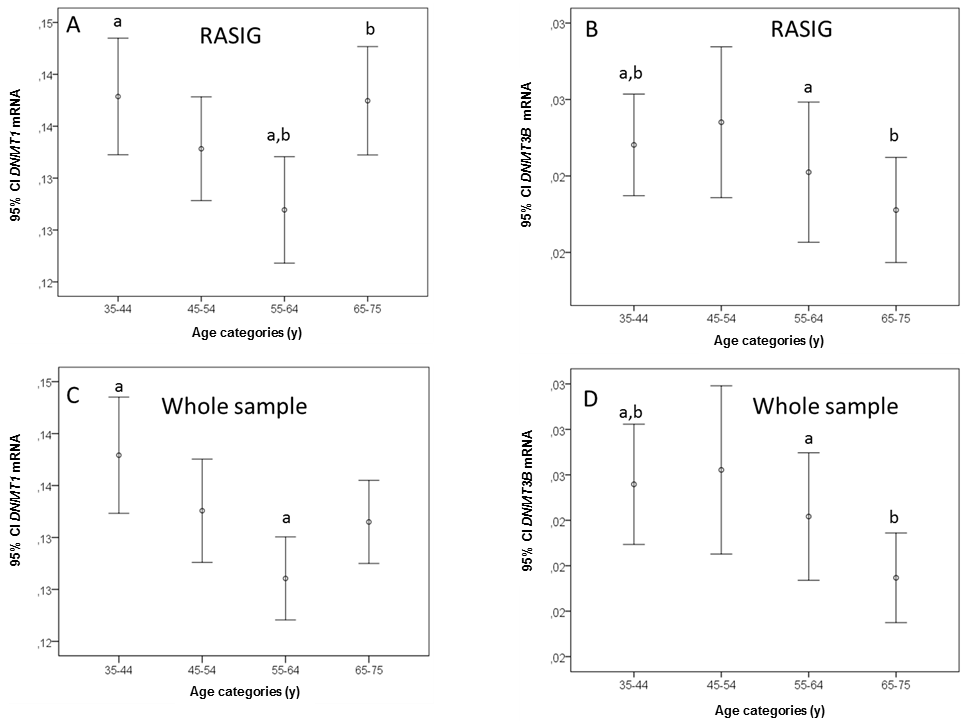
**

**Supplementary Figure 2. Age-related changes of *DNMT1* and *DNMT3B* mRNA levels with age in RASIG and in the whole population.** A graphical representation of the 95% confidence interval for the mean of *DNMT1* and *DNMT3B* mRNAs data in the RASIG sample (A and B) and in the whole population (B and D) is shown. The graph displays a U-shaped pattern for *DNMT1* mRNA that is more evident in RASIG than in the whole population and a linear decline for *DNMT3B* mRNA in subjects older than 45. Data sharing the same superscript are significantly different (p < 0.05 at least) by the Kruskal-Wallis test with pairwise comparison of subgroups (adjusted for multiple comparisons).


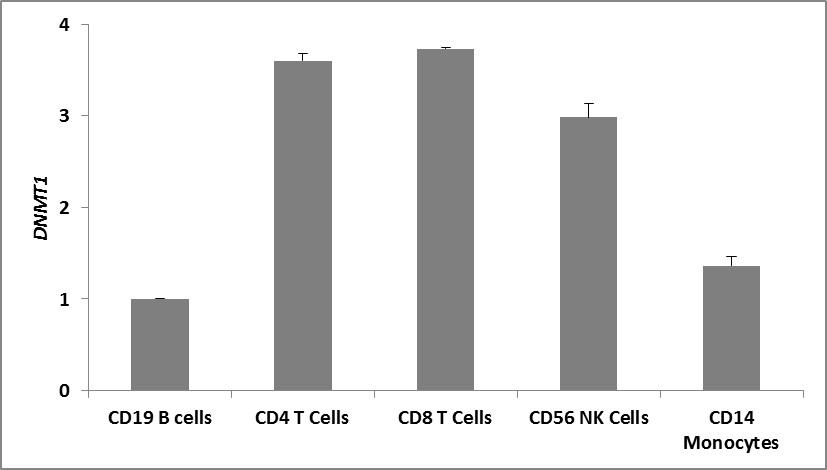


**
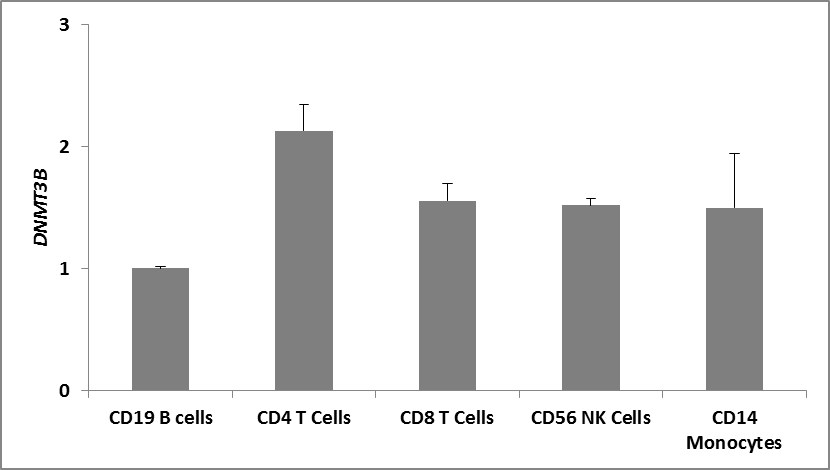
**

**Supplementary Figure 3. Transcript levels of *DNMT1* and *DNMT3B* in the different cell population present in PBMCs.** Analysis of the publicly available microarray dataset GSE1133 showing high expression levels of *DNMT1* in T-lymphocytes and NK cells (upper panel) and almost comparable expression levels of *DNMT3B* in the different cell populations (lower panel). Results are shown as mean +/- SEM.

**
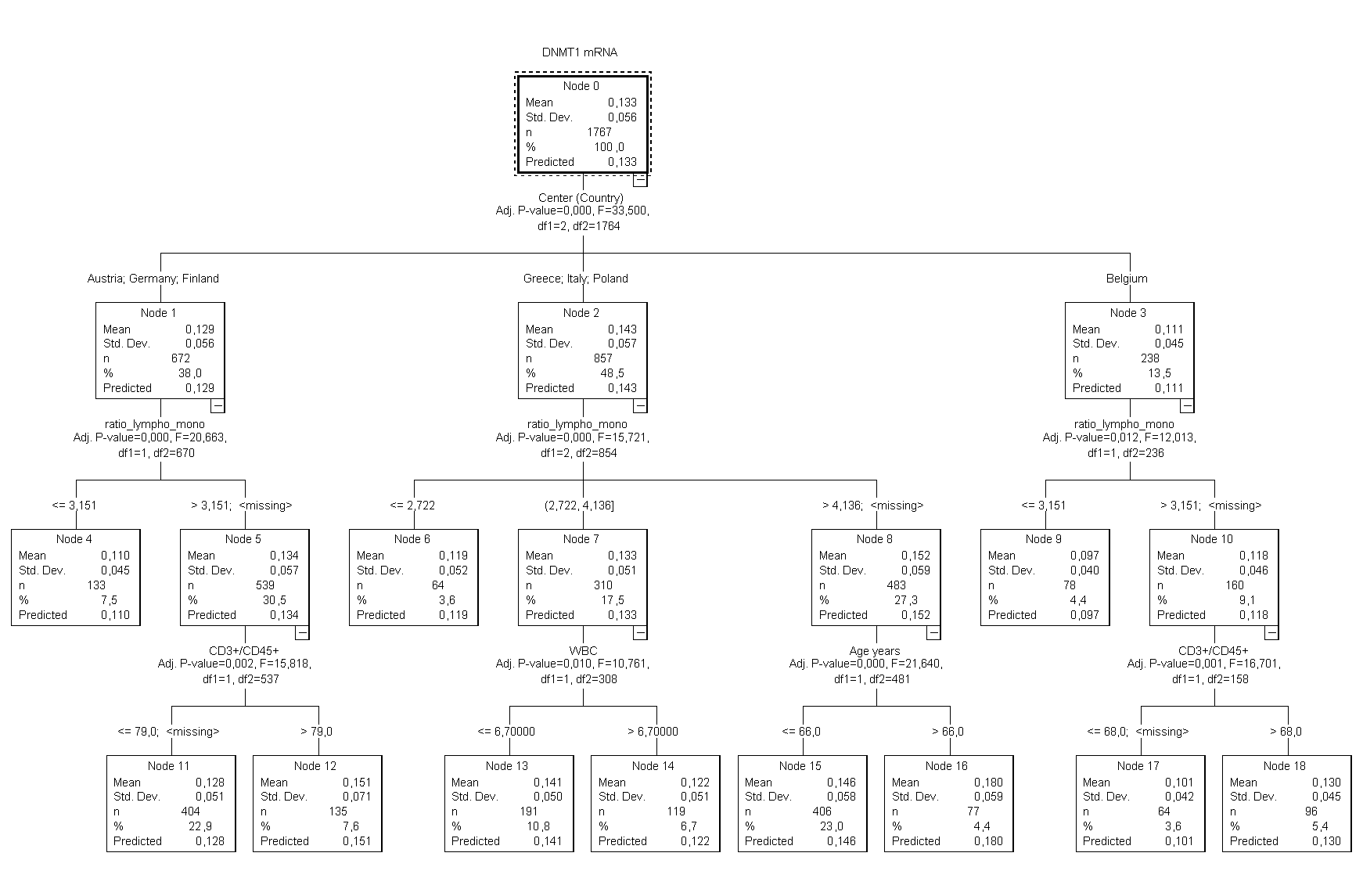
**

**Supplementary Figure 4. Identification of major variables affecting *DNMT1* mRNA levels by decision tree analysis.** Decision tree analysis of factors affecting *DNMT1* expression. The following variables were included in the model: recruitment centre [n,] gender [n], BMI [s], alcohol consumption [o], dairy products consumption [n], French fries consumption [n], brown bread consumption [o], white bread consumption [o], serum glucose [s], homocysteine [s], glycosylated haemoglobin A1C [s], MCH [s], MCV [s], HCT [s], RDW [s], WBC [s], neutrophils [s], monocytes [s], lymphocytes [s], lymphocyte to monocyte ratio [s], age [s], CD3+CD45+ cells [s]; ( n = nominal variable; o = ordinal variable; s = scale variable); Intervals for scale variables were fixed at 10. Missing data (“missing”), which might occur in some variables, are included in the analysis and are progressively clustered by the algorithm within the groups defined by the developing nodes. Definition of abbreviations is provided in the supplementary list.

**
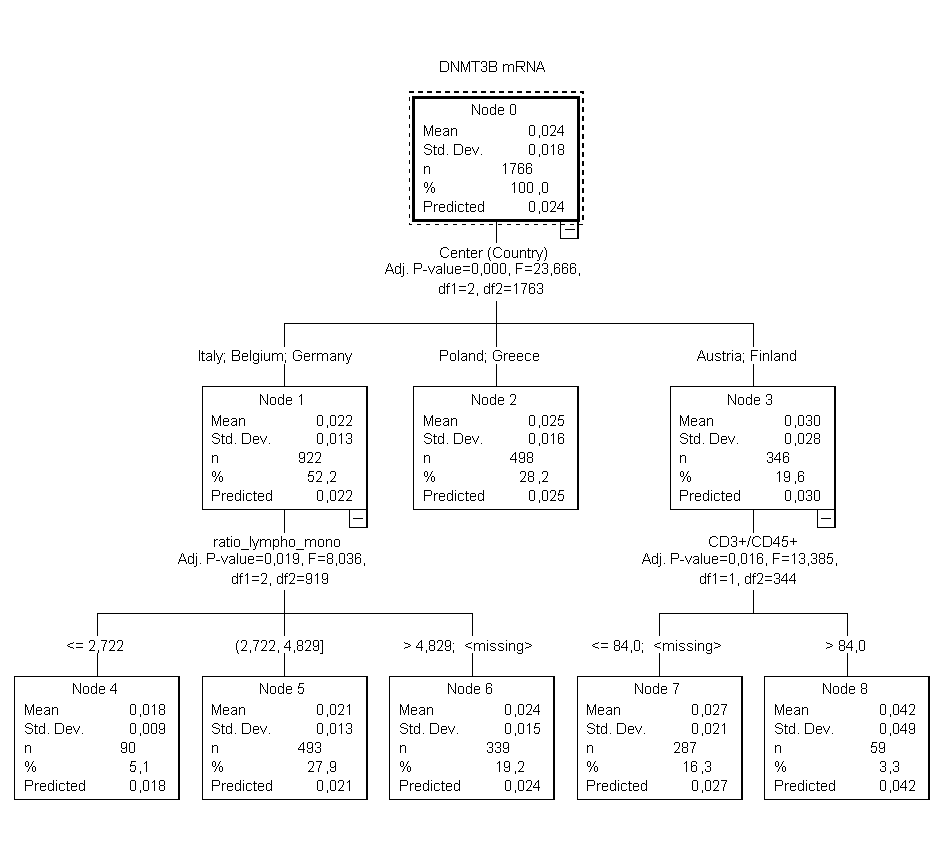
**

**Supplementary Figure 5. Identification of major variables affecting *DNMT3B* mRNA levels by decision tree analysis.** Decision tree analysis of factors affecting *DNMT3B* expression. The following variables were included in the model: recruitment centre [n], gender [n], platelets [s], monocytes [s], age years [s], HCT [s], MCHC [s], HGB [s], lymphocyte to monocyte ratio [s], CD3+CD45+ cells [s]; ( n = nominal variable; s = scale variable); Intervals for scale variables were fixed at 10. Missing data (“missing”), which might occur in some variables, are included in the analysis and are progressively clustered by the algorithm within the groups defined by the developing nodes. Definition of abbreviations is provided in the supplementary list.


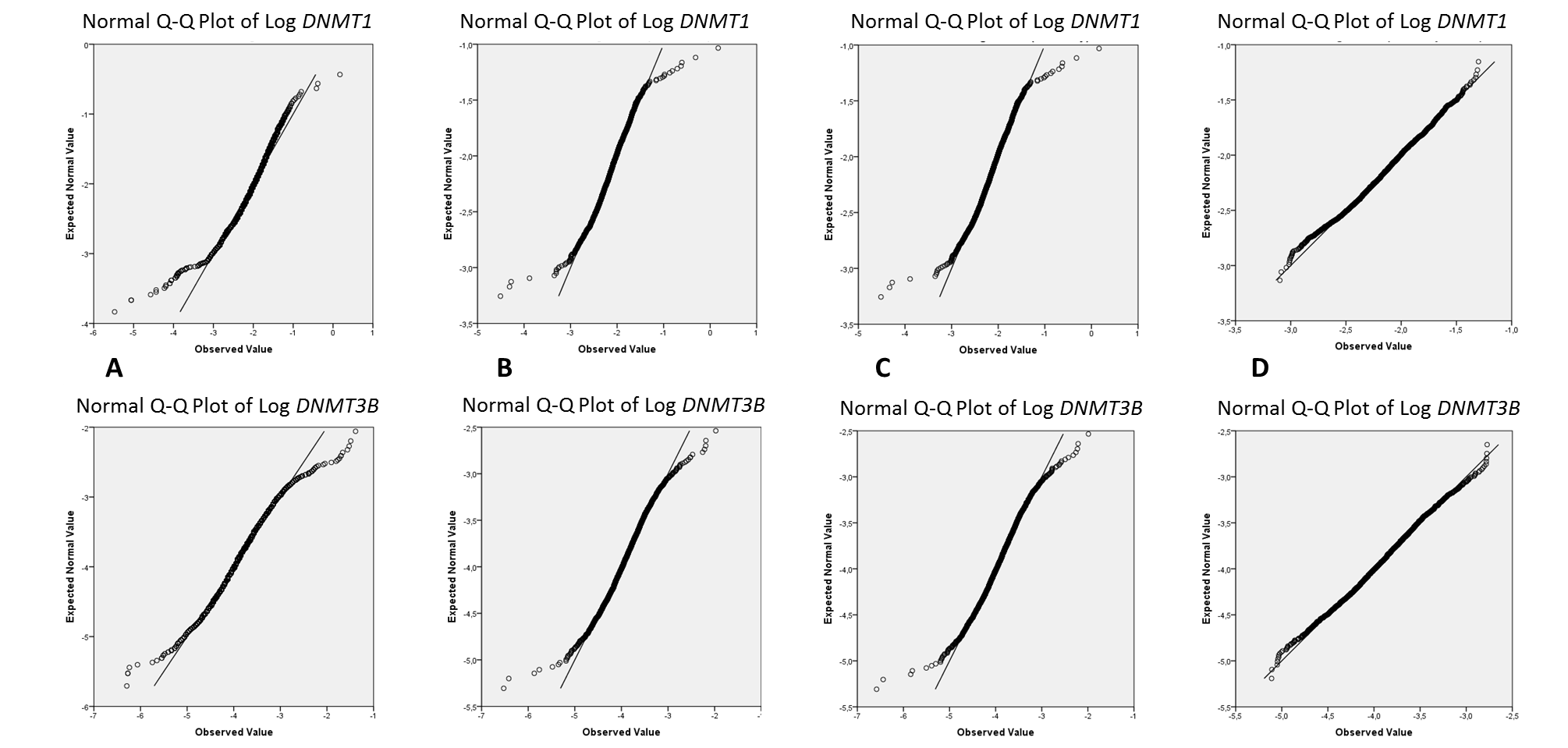


**Supplementary Figure 6**. **Impact of batch effect correction on normal Q-Q plots of *DNMT1* (upper panels) and *DNMT3B* (lower panels) expression.** A: normal Q-Q plots of uncorrected log-transformed variables; B: normal Q-Q plots of log-transformed variables after removal of batch effects by ANOVA; C: normal Q-Q plots of log-transformed variables after removal of batch effects by ANOVA and retaining eventual differences in group, age and gender; D: the same described in C after filtering outliers with a Z score higher than 3 or lower than -3.


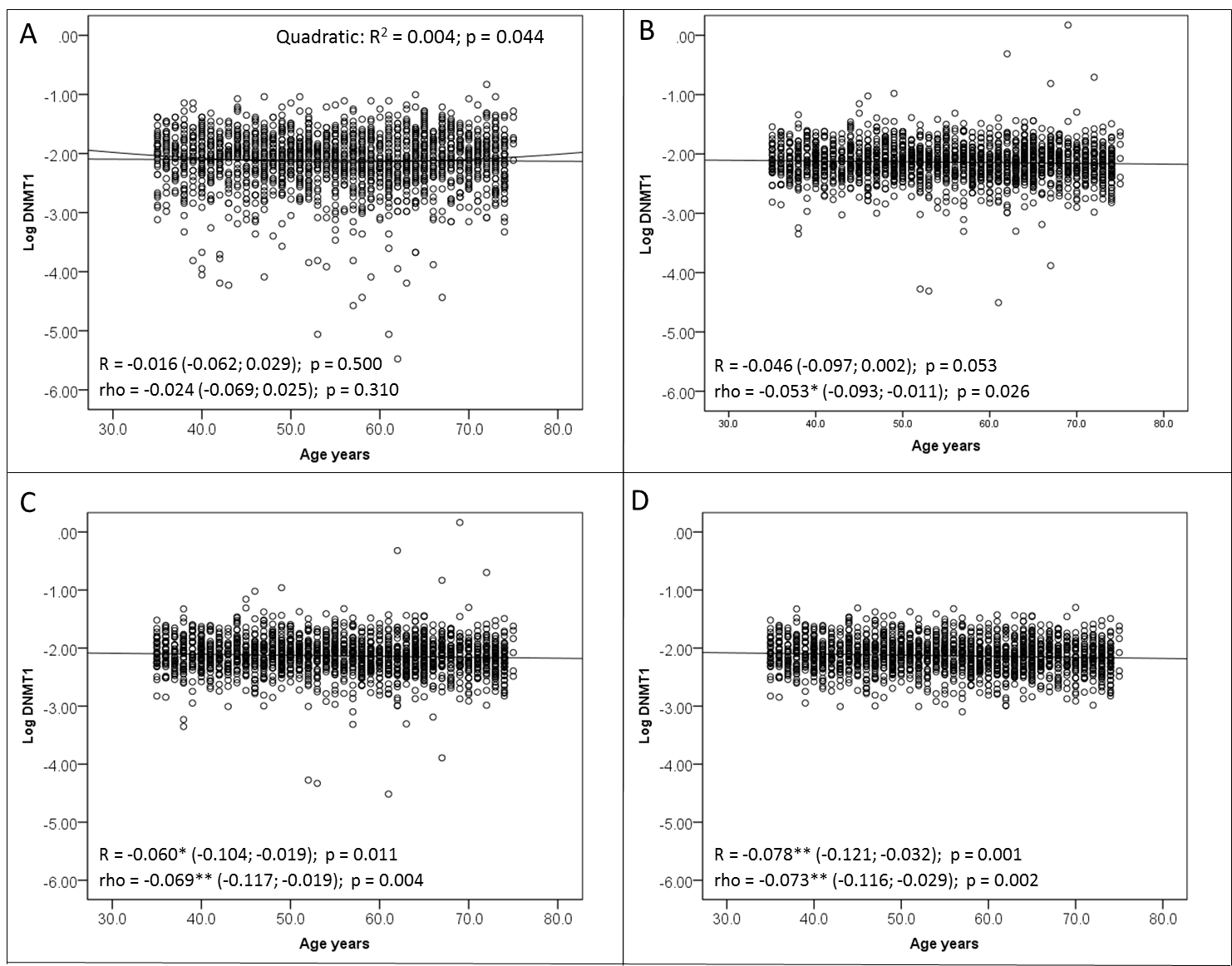


**Supplementary Figure 7**. **Impact of batch effect correction on the age-related changes of *DNMT1* expression in the RASIG population.** A: log-transformed *DNMT1* *vs.* age; B: log-transformed *DNMT1* vs. age after removal of batch effects by ANOVA; C: log-transformed *DNMT1* *vs*. age after removal of batch effects by ANOVA and retaining eventual differences in group, age and gender; D: the same described in C after filtering outliers with a Z score higher than 3 or lower than -3. All correlations (Pearson’s R and Spearman’s *rho*) reported below each graph are computed by bootstrapping with samples (1000) stratified for gender and country. A significant quadratic correlation was observed only for the data reported in panel A (as noted above the graph).


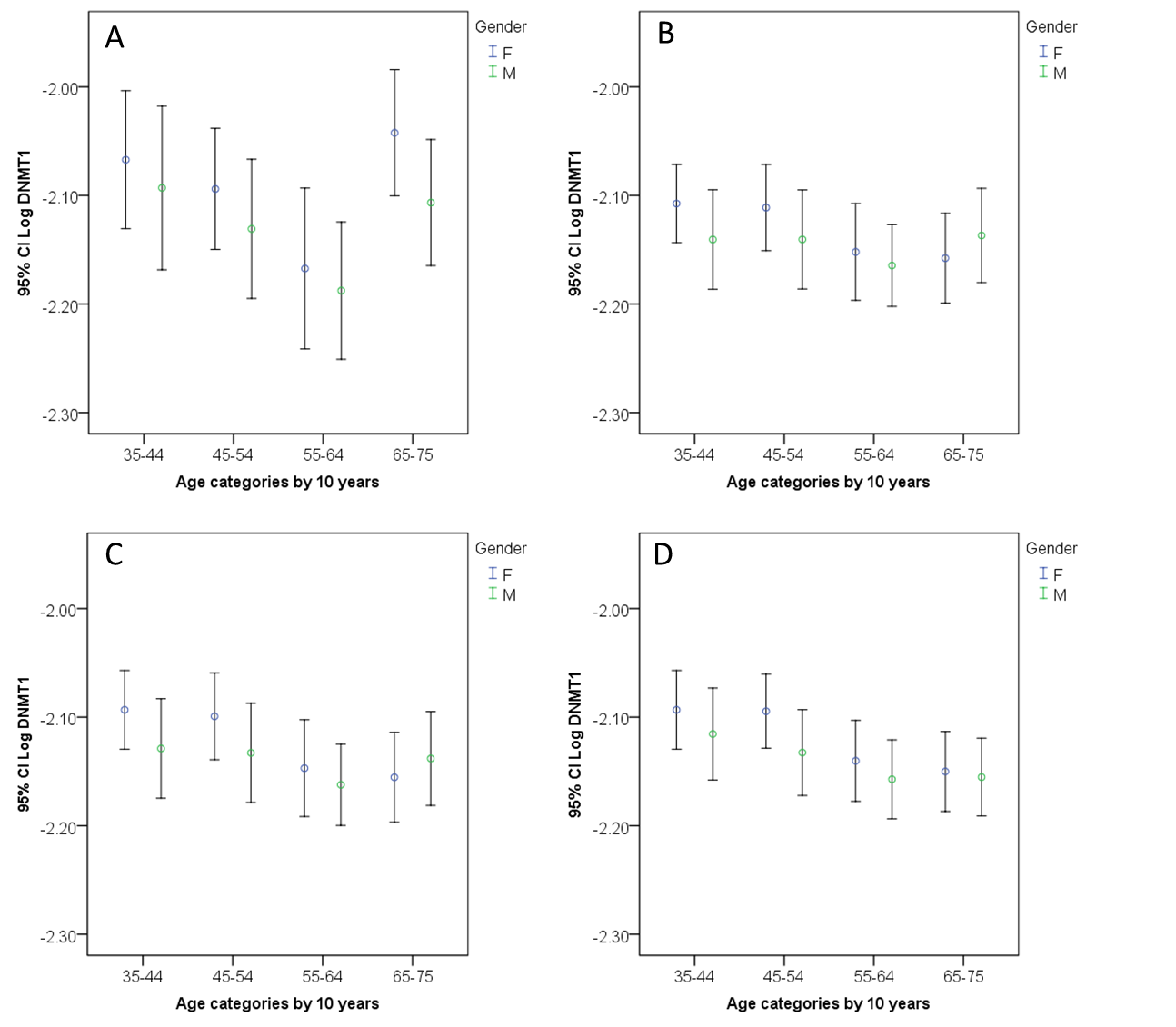


**Supplementary Figure 8. Impact of batch effect correction on the age-related changes of *DNMT1* expression in the RASIG population displayed as error bar and stratified for gender (green circles = males ; blue circles = females).** A: log-transformed *DNMT1* *vs.* age; B: log-transformed *DNMT1* *vs.* age after removal of batch effects by ANOVA; C: log-transformed *DNMT1* *vs.* age after removal of batch effects by ANOVA and retaining eventual differences in group, age and gender; D: the same described in C after filtering outliers with a Z score higher than 3 or lower than -3.

**
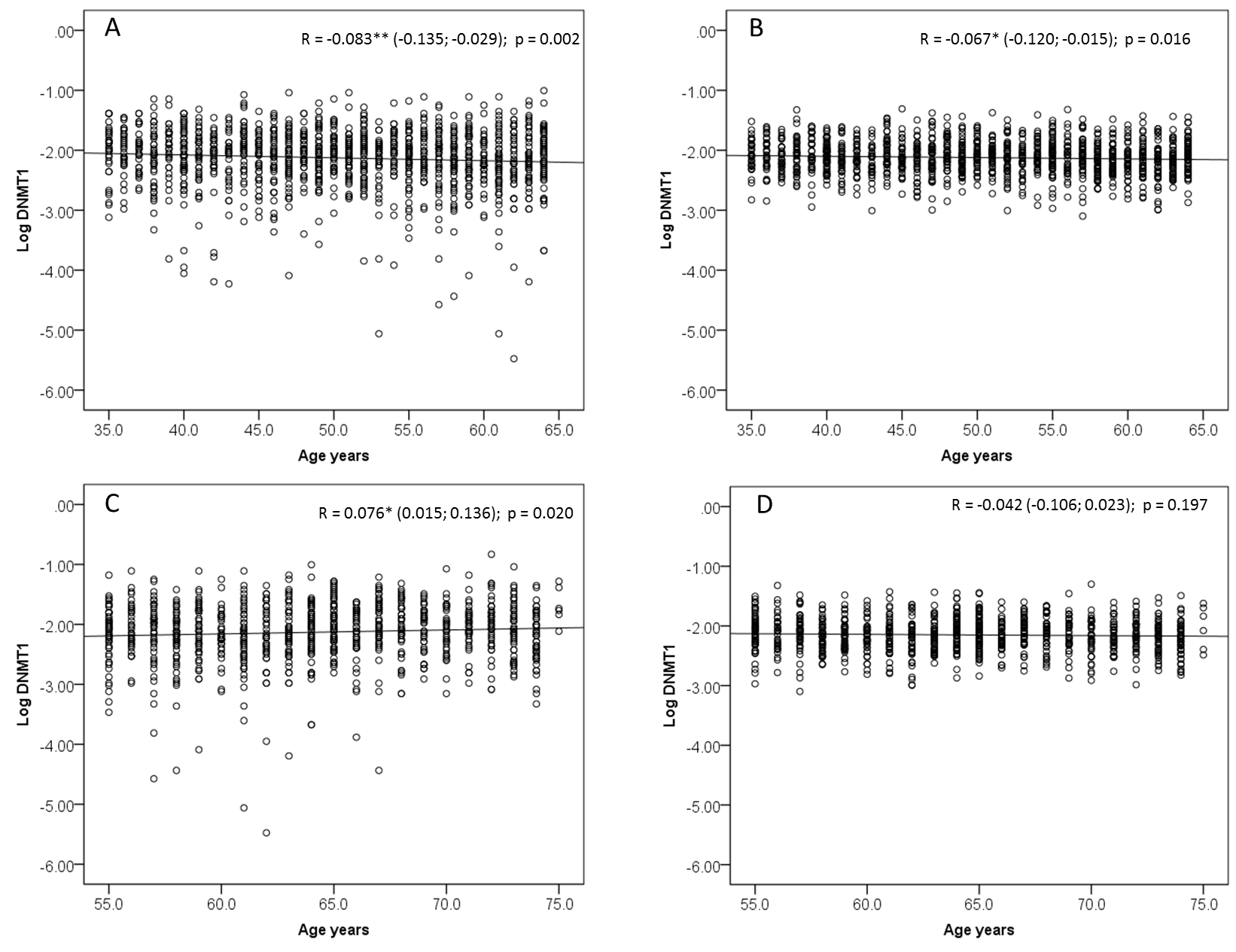
**

**Supplementary Figure 9. Impact of batch effect correction on the slope of *DNMT1* expression in the 35-64 and 55-75 age ranges.** A: log-transformed *DNMT1* *vs.* age in the range 35-64 years; B: the same as in A, but using log-transformed *DNMT1* data after removal of batch effects by ANOVA and filtering outliers with a Z score higher than 3 or lower than -3. C: log-transformed *DNMT1* *vs.* age in the range 55-75 years; D: the same as in C, but using log-transformed *DNMT1* data after removal of batch effects by ANOVA and filtering outliers with a Z score higher than 3 or lower than -3. All Pearson’s coefficients (R), significance and interval of confidence (reported within brackets) are computed by bootstrapping with samples (n = 1000) stratified for gender and country.


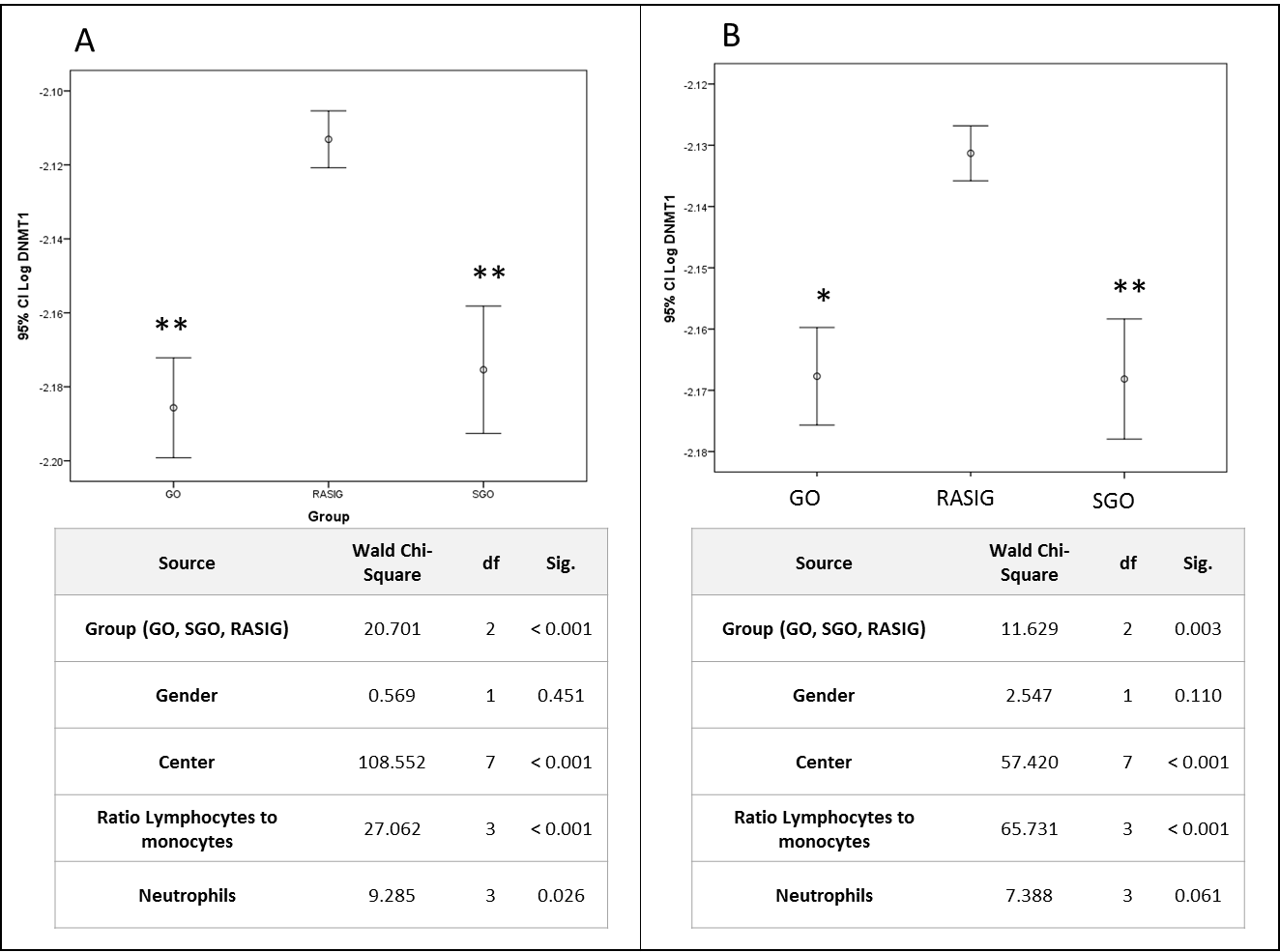


**Supplementary Figure 10**. **Impact of batch effect correction on group-related changes of *DNMT1* expression.** A: log-transformed *DNMT1* mean values (95% CI) in GO, SGO and RASIG after adjustment for the major confounding variables (group, gender, centre, ratio lymphocytes to monocyte, neutrophils); B: The same described in A using *DNMT1* values obtained after removal of batch effects (batch effects were removed by ANOVA, while retaining eventual differences in group, and applying a Z score +/-3 filter to remove overcorrected data). * p < 0.05 by the Bonferroni post-hoc test; ** p < 0.01 by the Bonferroni post-hoc test.


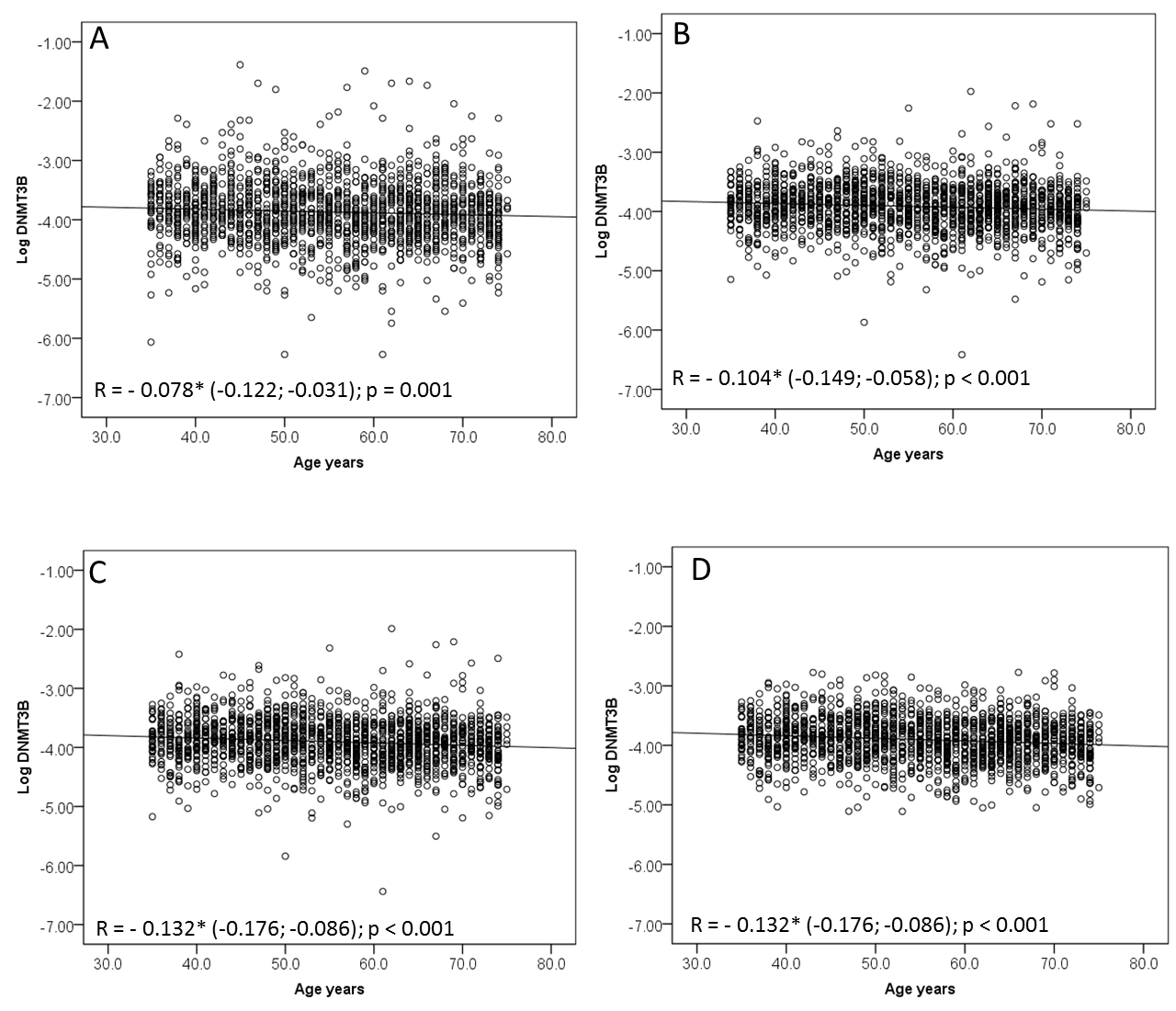


**Supplementary Figure 11**. **Impact of batch effect correction on the age-related changes of *DNMT3B* expression in the RASIG population.** A: log-transformed *DNMT3B* *vs.* age; B: log-transformed *DNMT3B* *vs.* age after removal of batch effects by ANOVA; C: log-transformed *DNMT3B* *vs.* age after removal of batch effects by ANOVA and retaining eventual differences in group, age and gender; D: the same described in C after filtering outliers with a Z score higher than 3 or lower than -3. All correlations reported below each graph and the respective confidence intervals of the coefficient (reported within brackets) are computed by bootstrapping with samples (1000) stratified for gender and country.


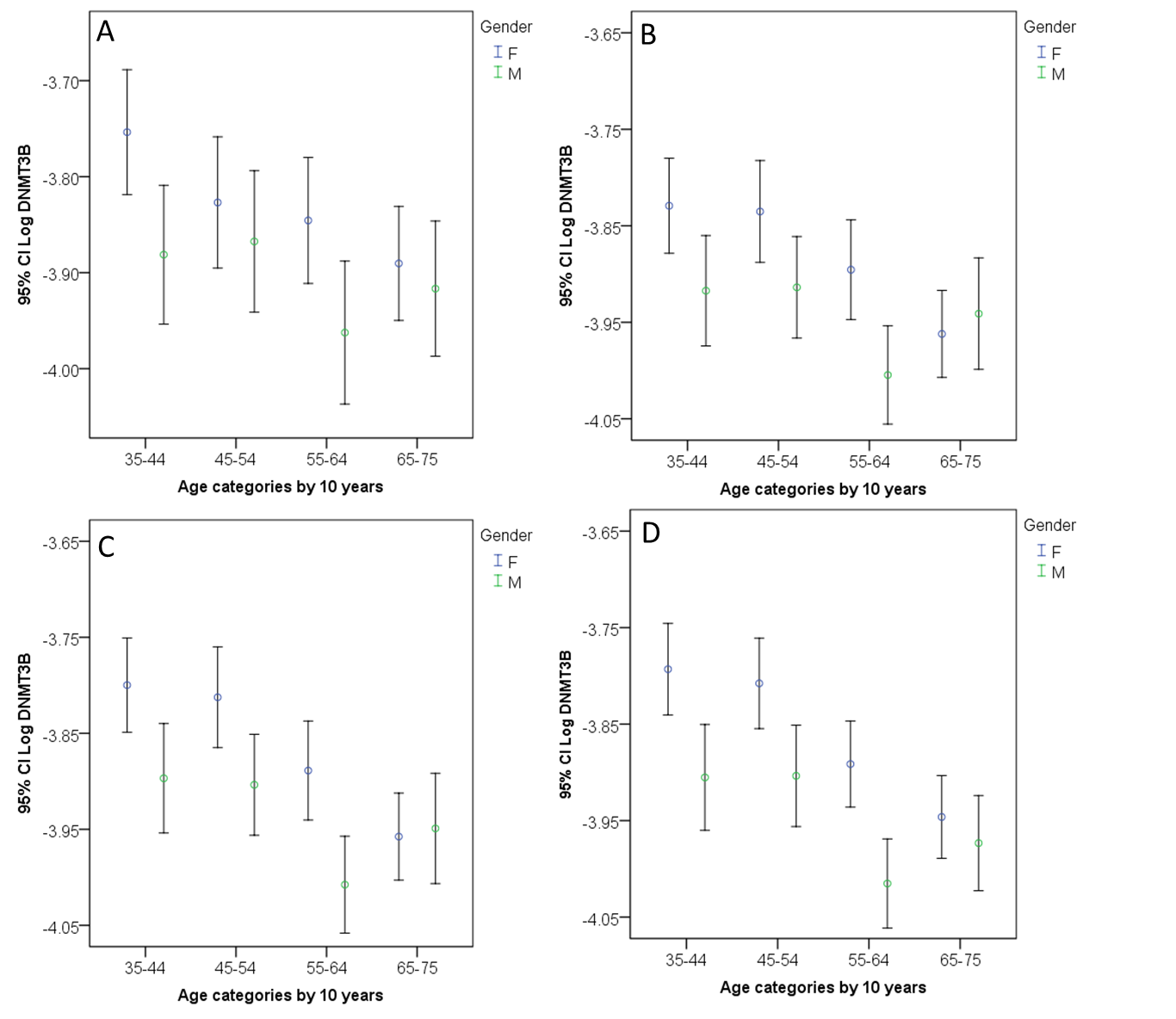


**Supplementary Figure 12.** **Impact of batch effect correction on the age-related changes of *DNMT3B* expression in the RASIG population displayed as error bar and stratified for gender.** A: log-transformed *DNMT3B* *vs.* age; B: log-transformed *DNMT3B* *vs.* age after removal of batch effects by ANOVA; C: log-transformed *DNMT3B* *vs.* age after removal of batch effects by ANOVA and retaining eventual differences in group, age and gender; D: The same described in C after filtering outliers with a Z score higher than 3 or lower than -3.

**Supplementary Table 1. Characteristics of the study population by age groups in each recruitment centre^1^**

| **Age range (y)** | | | | **35-75** | **35-44** | **44-54** | **55-64** | **65-75** | **p** |
| --- | --- | --- | --- | --- | --- | --- | --- | --- | --- |
|  | **Finland** | **N** | | 249 | 9 | 18 | 109 | 113 |  |
|  |  | **Age (y)** | | 62.3 ± 6.9 | 39.1 ± 3.0 | 51.3 ± 2.3 | 60.6 ± 2.7 | 67.4 ± 2.1 | < 0.01 |
|  |  | **Gender** | **F** | 63.9% (159) | 77.8% (7) | 66.7% (12) | 69.7% (76) | 56.6% (64) |  |
|  |  |  | **M** | 36.1% (90) | 22.2% (2) | 33.3% (6) | 30.3% (33) | 43.4% (49) |  |
|  |  | **Smoking habits** | **never** | 61.0% (152) | 88.9% (8) | 44.4% (8) | 66.1% (72) | 56.6% (64) |  |
|  |  |  | **former** | 31.7% (79) | 0.0% (0) | 50.0% (9) | 28.4% (31) | 34.5% (39) |  |
|  |  |  | **current** | 7.2% (18) | 11.1% (1) | 5.6% (1) | 5.5% (6) | 8.8% (10) |  |
|  |  | **Group** | **GO** | 48.6% (121) | 0.0% (0) | 33.3% (6) | 51.4% (56) | 52.2% (59) |  |
|  |  |  | **RASIG** | 32.1% (80) | 100.0% (9) | 38.9% (7) | 28.4% (31) | 29.2% (33) |  |
|  |  |  | **SGO** | 19.3% (48) | 0.0% (0) | 27.8% (5) | 20.2% (22) | 18.6% (21) |  |
|  |  | **BMI (Kg/m^2^)** | | 27.0 ± 5.0 | 23.2 ± 3.5 | 26.5 ± 4.9 | 27.4 ± 5.3 | 26.9 ± 4.8 | < 0.01 |
|  |  | **BMI classes** | **< 25** | 40.6% (101) | 77.8% (7) | 44.4% (8) | 34.9% (38) | 42.5% (48) |  |
|  |  |  | **25 to < 30** | 34.1% (85) | 11.1% (1) | 33.3% (6) | 35.8% (39) | 34.5% (39) |  |
|  |  |  | **>= 30** | 25.3% (63) | 11.1% (1) | 22.2% (4) | 29.4% (32) | 23.0% (26) |  |
|  | **Italy** | **N** | | 485 | 98 | 105 | 144 | 138 |  |
|  |  | **Age (y)** | | 56.1 ± 11.1 | 39.6 ± 2.9 | 49.9 ± 2.8 | 59.7 ± 2.9 | 68.9 ± 2.9 | < 0.01 |
|  |  | **Gender** | **F** | 50.1% (243) | 49.0% (48) | 52.4% (55) | 50.0% (72) | 49.3% (68) |  |
|  |  |  | **M** | 49.9% (242) | 51.0% (50) | 47.6% (50) | 50.0% (72) | 50.7% (70) |  |
|  |  | **Smoking habits** | **never** | 50.7% (246) | 53.1% (52) | 54.3% (57) | 44.4% (64) | 52.9% (73) | < 0.01 |
|  |  |  | **former** | 33.4% (162) | 20.4% (20) | 31.4% (33) | 44.4% (64) | 32.6% (45) |  |
|  |  |  | **current** | 15.9% (77) | 26.5% (26) | 14.3% (15) | 11.1% (16) | 14.5% (20) |  |
|  |  | **Group** | **GO** | 16.1% (78) | 1.0% (1) | 5.7% (6) | 21.5% (31) | 29.0% (40) | < 0.01 |
|  |  |  | **RASIG** | 74.6% (362) | 98.0% (96) | 90.5% (95) | 59.7% (86) | 61.6% (85) |  |
|  |  |  | **SGO** | 9.3% (45) | 1.0% (1) | 3.8% (4) | 18.8% (27) | 9.4% (13) |  |
|  |  | **BMI (Kg/m^2^)** | | 26.5 ± 4.8 | 24.5 ± 4.4 | 25.3 ± 4.3 | 27.2 ± 5.0 | 28.3 ± 4.4 | < 0.01 |
|  |  | **BMI classes** | **< 25** | 44.1% (214) | 63.3% (62) | 55.2% (58) | 41.0% (59) | 25.4% (35) | < 0.01 |
|  |  |  | **25 to < 30** | 34.4% (167) | 26.5% (26) | 33.3% (35) | 31.9% (46) | 43.5% (60) |  |
|  |  |  | **>= 30** | 21.4% (104) | 10.2% (10) | 11.4% (12) | 27.1% (39) | 31.2% (43) |  |
|  | **Austria** | **N** | | 267 | 69 | 69 | 69 | 60 |  |
|  |  | **Age (y)** | | 54.1 ± 11.4 | 39.6 ± 2.8 | 49.6 ± 2.9 | 59.8 ± 2.9 | 69.5 ± 2.7 | < 0.01 |
|  |  | **Gender** | **F** | 49.4% (132) | 47.8% (33) | 46.4% (32) | 50.7% (35) | 53.3% (32) |  |
|  |  |  | **M** | 50.6% (135) | 52.2% (36) | 53.6% (37) | 49.3% (34) | 46.7% (28) |  |
|  |  | **Smoking habits** | **never** | 55.8% (149) | 68.1% (47) | 47.8% (33) | 40.6% (28) | 68.3% (41) | < 0.01 |
|  |  |  | **former** | 32.2% (86) | 18.8% (13) | 34.8% (24) | 49.3% (34) | 25.0% (15) |  |
|  |  |  | **current** | 12.0% (32) | 13.0% (9) | 17.4% (12) | 10.1% (7) | 6.7% (4) |  |
|  |  | **Group** | **GO** | 0.0% (0) | 0.0% (0) | 0.0% (0) | 0.0% (0) | 0.0% (0) |  |
|  |  |  | **RASIG** | 100.0% (267) | 100.0% (69) | 100.0% (69) | 100.0% (69) | 100.0% (60) |  |
|  |  |  | **SGO** | 0.0% (0) | 0.0% (0) | 0.0% (0) | 0.0% (0) | 0.0% (0) |  |
|  |  | **BMI (Kg/m^2^)** | | 25.4 ± 4.3 | 25.2 ± 5.7 | 24.8 ± 3.2 | 25.5 ± 4.1 | 26.1 ± 3.6 | < 0.01 |
|  |  | **BMI classes** | **< 25** | 51.3% (137) | 56.5% (39) | 50.7% (35) | 52.2% (36) | 45.0% (27) |  |
|  |  |  | **25 to < 30** | 37.8% (101) | 33.3% (23) | 44.9% (31) | 34.8% (24) | 38.3% (23) |  |
|  |  |  | **>= 30** | 10.9% (29) | 10.1% (7) | 4.3% (3) | 13.0% (9) | 16.7% (10) |  |
|  | **Greece** | **N** | | 317 | 71 | 78 | 86 | 82 |  |
|  |  | **Age (y)** | | 55.1 ± 11.5 | 39.4 ± 2.6 | 49.3 ± 2.9 | 59.2 ± 3.0 | 69.7 ± 3.4 | < 0.01 |
|  |  | **Gender** | **F** | 50.8% (161) | 47.9% (34) | 52.6% (41) | 48.8% (42) | 53.7% (44) |  |
|  |  |  | **M** | 49.2% (156) | 52.1% (37) | 47.4% (37) | 51.2% (44) | 46.3% (38) |  |
|  |  | **Smoking habits** | **never** | 45.1% (143) | 46.5% (33) | 34.6% (27) | 37.2% (32) | 62.2% (51) | < 0.01 |
|  |  |  | **former** | 19.6% (62) | 9.9% (7) | 25.6% (20) | 20.9% (18) | 20.7% (17) |  |
|  |  |  | **current** | 35.3% (112) | 43.7% (31) | 39.7% (31) | 41.9% (36) | 17.1% (14) |  |
|  |  | **Group** | **GO** | 5.4% (17) | 0.0% (0) | 1.3% (1) | 12.8% (11) | 6.1% (5) | < 0.01 |
|  |  |  | **RASIG** | 93.4% (296) | 100.0% (71) | 97.4% (76) | 86.0% (74) | 91.5% (75) |  |
|  |  |  | **SGO** | 1.3% (4) | 0.0% (0) | 1.3% (1) | 1.2% (1) | 2.4% (2) |  |
|  |  | **BMI (Kg/m^2^)** | | 27.5 ± 4.9 | 25.4 ± 4.7 | 27.0 ± 4.8 | 28.2 ± 4.3 | 29.0 ± 5.1 | < 0.01 |
|  |  | **BMI classes** | **< 25** | 33.2% (105) | 57.7% (41) | 38.5% (30) | 19.8% (17) | 21.0% (17) | < 0.01 |
|  |  |  | **25 to < 30** | 40.8% (129) | 25.4% (18) | 37.2% (29) | 50.0% (43) | 48.1% (39) |  |
|  |  |  | **>= 30** | 25.9% (82) | 16.9% (12) | 24.4% (19) | 30.2% (26) | 30.9% (25) |  |
|  | **Poland** | **N** | | 270 | 46 | 52 | 99 | 73 |  |
|  |  | **Age (y)** | | 57.0 ± 10.8 | 39.9 ± 3.0 | 49.7 ± 2.7 | 59.4 ± 3.0 | 69.8 ± 3.2 | < 0.01 |
|  |  | **Gender** | **F** | 54.8% (148) | 60.9% (28) | 50.0% (26) | 50.5% (50) | 60.3% (44) |  |
|  |  |  | **M** | 45.2% (122) | 39.1% (18) | 50.0% (26) | 49.5% (49) | 39.7% (29) |  |
|  |  | **Smoking habits** | **never** | 46.3% (125) | 63.0% (29) | 32.7% (17) | 41.4% (41) | 52.1% (38) | < 0.01 |
|  |  |  | **former** | 30.4% (82) | 21.7% (10) | 38.5% (20) | 25.3% (25) | 37.0% (27) |  |
|  |  |  | **current** | 23.3% (63) | 15.2% (7) | 28.8% (15) | 33.3% (33) | 11.0% (8) |  |
|  |  | **Group** | **GO** | 15.9% (43) | 0.0% (0) | 5.8% (3) | 22.2% (22) | 24.7% (18) | < 0.01 |
|  |  |  | **RASIG** | 74.8% (202) | 100.0% (46) | 90.4% (47) | 61.6% (61) | 65.8% (48) |  |
|  |  |  | **SGO** | 9.3% (25) | 0.0% (0) | 3.8% (2) | 16.2% (16) | 9.6% (7) |  |
|  |  | **BMI (Kg/m^2^)** | | 27.3 ± 4.7 | 26.3 ± 4.0 | 28.1 ± 5.9 | 27.0 ± 4.6 | 27.7 ± 4.3 | < 0.01 |
|  |  | **BMI classes** | **< 25** | 33.0% (89) | 45.7% (21) | 36.5% (19) | 32.3% (32) | 23.3% (17) |  |
|  |  |  | **25 to < 30** | 43.7% (118) | 34.8% (16) | 30.8% (16) | 48.5% (48) | 52.1% (38) |  |
|  |  |  | **>= 30** | 23.3% (63) | 19.6% (9) | 32.7% (17) | 19.2% (19) | 24.7% (18) |  |
|  | **The Netherlands** | **N** | | 189 | 0 | 3 | 79 | 107 |  |
|  |  | **Age (y)** | | 64.9 ± 4.7 | 0.0 ± 0.0 | 53.7 ± 0.6 | 60.8 ± 2.4 | 68.2 ± 2.9 | < 0.01 |
|  |  | **Gender** | **F** | 48.7% (92) | 0.0% (0) | 100.0% (3) | 57.0% (45) | 41.1% (44) | < 0.01 |
|  |  |  | **M** | 51.3% (97) | 0.0% (0) | 0.0% (0) | 43.0% (34) | 58.9% (63) |  |
|  |  | **Smoking habits** | **never** | 38.6% (73) | 0.0% (0) | 66.7% (2) | 40.5% (32) | 36.4% (39) |  |
|  |  |  | **former** | 55.6% (105) | 0.0% (0) | 33.3% (1) | 54.4% (43) | 57.0% (61) |  |
|  |  |  | **current** | 5.8% (11) | 0.0% (0) | 0.0% (0) | 5.1% (4) | 6.5% (7) |  |
|  |  | **Group** | **GO** | 55.0% (104) | 0.0% (0) | 33.3% (1) | 54.4% (43) | 56.1% (60) |  |
|  |  |  | **RASIG** | 0.0% (0) | 0.0% (0) | 0.0% (0) | 0.0% (0) | 0.0% (0) |  |
|  |  |  | **SGO** | 45.0% (85) | 0.0% (0) | 66.7% (2) | 45.6% (36) | 43.9% (47) |  |
|  |  | **BMI (Kg/m^2^)** | | 26.3 ± 3.5 | 0.0 ± 0.0 | 24.1 ± 2.5 | 26.0 ± 3.7 | 26.7 ± 3.3 | < 0.01 |
|  |  | **BMI classes** | **< 25** | 33.3% (63) | 0.0% (0) | 66.7% (2) | 38.0% (30) | 29.0% (31) |  |
|  |  |  | **25 to < 30** | 52.9% (100) | 0.0% (0) | 33.3% (1) | 50.6% (40) | 55.1% (59) |  |
|  |  |  | **>= 30** | 13.8% (26) | 0.0% (0) | 0.0% (0) | 11.4% (9) | 15.9% (17) |  |
|  | **Belgium** | **N** | | 350 | 30 | 69 | 131 | 120 |  |
|  |  | **Age (y)** | | 59.5 ± 9.7 | 40.2 ± 2.8 | 49.7 ± 2.7 | 59.9 ± 2.7 | 69.7 ± 3.1 | < 0.01 |
|  |  | **Gender** | **F** | 54.6% (191) | 60.0% (18) | 58.0% (40) | 55.0% (72) | 50.8% (61) |  |
|  |  |  | **M** | 45.4% (159) | 40.0% (12) | 42.0% (29) | 45.0% (59) | 49.2% (59) |  |
|  |  | **Smoking habits** | **never** | 56.3% (197) | 63.3% (19) | 60.9% (42) | 58.0% (76) | 50.0% (60) |  |
|  |  |  | **former** | 38.0% (133) | 23.3% (7) | 29.0% (20) | 37.4% (49) | 47.5% (57) |  |
|  |  |  | **current** | 5.7% (20) | 13.3% (4) | 10.1% (7) | 4.6% (6) | 2.5% (3) |  |
|  |  | **Group** | **GO** | 21.1% (74) | 0.0% (0) | 0.0% (0) | 32.1% (42) | 26.7% (32) | < 0.01 |
|  |  |  | **RASIG** | 68.9% (241) | 100.0% (30) | 94.2% (65) | 55.0% (72) | 61.7% (74) |  |
|  |  |  | **SGO** | 10.0% (35) | 0.0% (0) | 5.8% (4) | 13.0% (17) | 11.7% (14) |  |
|  |  | **BMI (Kg/m^2^)** | | 25.6 ± 4.0 | 23.9 ± 3.0 | 24.8 ± 3.7 | 26.2 ± 4.4 | 25.7 ± 3.6 | < 0.05 |
|  |  | **BMI classes** | **< 25** | 48.6% (170) | 63.3% (19) | 58.0% (40) | 41.2% (54) | 47.5% (57) |  |
|  |  |  | **25 to < 30** | 39.1% (137) | 30.0% (9) | 30.4% (21) | 44.3% (58) | 40.8% (49) |  |
|  |  |  | **>= 30** | 12.3% (43) | 6.7% (2) | 11.6% (8) | 14.5% (19) | 11.7% (14) |  |
|  | **Germany** | **N** | | 326 | 60 | 90 | 90 | 86 |  |
|  |  | **Age (y)** | | 55.6 ± 11.0 | 39.7 ± 2.7 | 49.2 ± 2.7 | 59.6 ± 2.8 | 69.4 ± 2.9 | < 0.01 |
|  |  | **Gender** | **F** | 55.8% (182) | 63.3% (38) | 54.4% (49) | 54.4% (49) | 53.5% (46) |  |
|  |  |  | **M** | 44.2% (144) | 36.7% (22) | 45.6% (41) | 45.6% (41) | 46.5% (40) |  |
|  |  | **Smoking habits** | **never** | 54.9% (179) | 65.0% (39) | 52.2% (47) | 53.3% (48) | 52.3% (45) | < 0.01 |
|  |  |  | **former** | 35.9% (117) | 15.0% (9) | 38.9% (35) | 36.7% (33) | 46.5% (40) |  |
|  |  |  | **current** | 9.2% (30) | 20.0% (12) | 8.9% (8) | 10.0% (9) | 1.2% (1) |  |
|  |  | **Group** | **GO** | 0.0% (0) | 0.0% (0) | 0.0% (0) | 0.0% (0) | 0.0% (0) |  |
|  |  |  | **RASIG** | 100.0% (326) | 100.0% (60) | 100.0% (90) | 100.0% (90) | 100.0% (86) |  |
|  |  |  | **SGO** | 0.0% (0) | 0.0% (0) | 0.0% (0) | 0.0% (0) | 0.0% (0) |  |
|  |  | **BMI (Kg/m^2^)** | | 25.0 ± 3.5 | 24.5 ± 4.2 | 24.7 ± 3.2 | 25.2 ± 3.3 | 25.4 ± 3.4 | < 0.01 |
|  |  | **BMI classes** | **< 25** | 55.2% (180) | 61.7% (37) | 57.8% (52) | 56.7% (51) | 46.5% (40) |  |
|  |  |  | **25 to < 30** | 36.8% (120) | 26.7% (16) | 35.6% (32) | 36.7% (33) | 45.3% (39) |  |
|  |  |  | **>= 30** | 8.0% (26) | 11.7% (7) | 6.7% (6) | 6.7% (6) | 8.1% (7) |  |
|  |  |  |  |  |  |  |  |  |  |

^1^ Values are mean ± SD and percentage (number), all such variables; one missing case for BMI; p-value: one-way-ANOVA (continuous variables) and Chi-square test (prevalence). Definition of abbreviations is provided in the supplementary list.

**Supplementary Table 2. Effect of age, gender, BMI on *DNMT1* and *DNMT3B* expression in the RASIG population of each recruitment centre.**

|  | | | |  |  | *DNMT1* mRNA |  |  | *DNMT3B* mRNA |  |  |
| --- | --- | --- | --- | --- | --- | --- | --- | --- | --- | --- | --- |
|  |  |  |  | **Stat** | **N** | **Median (IQ)** | **p (KW)^1^** | **p (GLM)^2^** | **Median (IQ)** | **p (KW)^1^** | **p (GLM)^2^** |
|  | **Finland** | **Age Group (y)** | **35-44** | a | 9 | 0.129 (0.125 - 0.159) | 0.154 | 0.208 | 0.031 (0.023 - 0.033) | 0.470 | 0.660 |
|  |  |  | **45-54** | b | 7 | 0.134 (0.117 - 0.159) |  |  | 0.019 (0.016 - 0.027) |  |  |
|  |  |  | **55-64** | c | 31 | 0.109 (0.082 - 0.129) |  |  | 0.025 (0.019 - 0.031) |  |  |
|  |  |  | **65-75** | d | 33 | 0.125 (0.092 - 0.171) |  |  | 0.022 (0.016 - 0.028) |  |  |
|  |  | **Gender** | **F** | a | 57 | 0.113 (0.092 - 0.149) | 0.184 | 0.404 | 0.024 (0.018 - 0.033) | 0.877 | 0.726 |
|  |  |  | **M** | b | 23 | 0.139 (0.095 - 0.159) |  |  | 0.024 (0.019 - 0.026) |  |  |
|  |  | **BMI classes** | **< 25** | a | 32 | 0.125 (0.096 - 0.149) | 0.696 | 0.694 | 0.026 (0.020 - 0.034) | 0.280 | 0.593 |
|  |  |  | **25 to < 30** | b | 27 | 0.117 (0.092 - 0.149) |  |  | 0.023 (0.018 - 0.027) |  |  |
|  |  |  | **>= 30** | c | 21 | 0.109 (0.082 - 0.159) |  |  | 0.022 (0.016 - 0.027) |  |  |
|  | **Italy** | **Age Group (y)** | **35-44** | a | 96 | 0.144 (0.115 - 0.174)^d^ | *< 0.001* | *0.001* | 0.021 (0.015 - 0.027) | 0.533 | 0.358 |
|  |  |  | **45-54** | b | 95 | 0.129 (0.102 - 0.171)^d^ |  |  | 0.021 (0.016 - 0.027) |  |  |
|  |  |  | **55-64** | c | 86 | 0.139 (0.095 - 0.183)^d^ |  |  | 0.019 (0.015 - 0.025) |  |  |
|  |  |  | **65-75** | d | 85 | 0.171 (0.134 - 0.203)^a.b.c^ |  |  | 0.020 (0.015 - 0.025) |  |  |
|  |  | **Gender** | **F** | a | 182 | 0.141 (0.102 - 0.183) | 0.757 | 0.887 | 0.021 (0.016 - 0.025) | 0.444 | 0.507 |
|  |  |  | **M** | b | 180 | 0.144 (0.115 - 0.183) |  |  | 0.019 (0.015 - 0.025) |  |  |
|  |  | **BMI classes** | **< 25** | a | 174 | 0.139 (0.102 - 0.177) | 0.136 | 0.867 | 0.019 (0.015 - 0.025) | 0.466 | 0.240 |
|  |  |  | **25 to < 30** | b | 121 | 0.139 (0.117 - 0.183) |  |  | 0.021 (0.016 - 0.025) |  |  |
|  |  |  | **>= 30** | c | 67 | 0.149 (0.121 - 0.189) |  |  | 0.021 (0.016 - 0.027) |  |  |
|  | **Austria** | **Age Group (y)** | **35-44** | a | 69 | 0.125 (0.102 - 0.183)^c^ | *0.001* | 0.001 | 0.023 (0.019 - 0.035) | 0.442 | 0.548 |
|  |  |  | **45-54** | b | 69 | 0.125 (0.082 - 0.177)^c^ |  |  | 0.025 (0.013 - 0.033) |  |  |
|  |  |  | **55-64** | c | 69 | 0.098 (0.067 - 0.117)^a.b^ |  |  | 0.021 (0.012 - 0.036) |  |  |
|  |  |  | **65-75** | d | 60 | 0.113 (0.077 - 0.149) |  |  | 0.022 (0.017 - 0.032) |  |  |
|  |  | **Gender** | **F** | a | 132 | 0.113 (0.074 - 0.157) | 0.973 | 0.554 | 0.023 (0.016 - 0.036) | 0.377 | 0.649 |
|  |  |  | **M** | b | 135 | 0.113 (0.085 - 0.165) |  |  | 0.021 (0.015 - 0.033) |  |  |
|  |  | **BMI classes** | **< 25** | a | 137 | 0.109 (0.074 - 0.154) | 0.301 | 0.542 | 0.021 (0.014 - 0.033) | 0.368 | 0.421 |
|  |  |  | **25 to < 30** | b | 101 | 0.113 (0.088 - 0.165) |  |  | 0.024 (0.016 - 0.035) |  |  |
|  |  |  | **>= 30** | c | 29 | 0.134 (0.105 - 0.154) |  |  | 0.026 (0.019 - 0.036) |  |  |
|  | **Greece** | **Age Group (y)** | **35-44** | a | 71 | 0.134 (0.095 - 0.177) | *0.028* | *0.034* | 0.024 (0.017 - 0.032) | 0.159 | 0.302 |
|  |  |  | **45-54** | b | 76 | 0.127 (0.088 - 0.165) |  |  | 0.021 (0.017 - 0.035) |  |  |
|  |  |  | **55-64** | c | 74 | 0.117 (0.085 - 0.159)^d^ |  |  | 0.019 (0.015 - 0.027) |  |  |
|  |  |  | **65-75** | d | 75 | 0.149 (0.105 - 0.183)^c^ |  |  | 0.022 (0.016 - 0.031) |  |  |
|  |  | **Gender** | **F** | a | 147 | 0.154 (0.117 - 0.196)^b^ | *<0.001* | *<0.001* | 0.024 (0.017 - 0.035)^b^ | *0.003* | *0.008* |
|  |  |  | **M** | b | 149 | 0.109 (0.074 - 0.149)^a^ |  |  | 0.020 (0.015 - 0.027)^a^ |  |  |
|  |  | **BMI classes** | **< 25** | a | 100 | 0.134 (0.105 - 0.177) | 0.153 | 0.746 | 0.022 (0.016 - 0.033) | 0.328 | 0.325 |
|  |  |  | **25 to < 30** | b | 118 | 0.125 (0.085 - 0.165) |  |  | 0.020 (0.016 - 0.027) |  |  |
|  |  |  | **>= 30** | c | 77 | 0.129 (0.092 - 0.165) |  |  | 0.023 (0.016 - 0.032) |  |  |
|  | **Poland** | **Age Group (y)** | **35-44** | a | 46 | 0.149 (0.117 - 0.177) | 0.599 | 0.656 | 0.022 (0.019 - 0.028) | 0.042 | 0.289 |
|  |  |  | **45-54** | b | 47 | 0.139 (0.109 - 0.189) |  |  | 0.024 (0.018 - 0.028) |  |  |
|  |  |  | **55-64** | c | 61 | 0.139 (0.105 - 0.171) |  |  | 0.021 (0.015 - 0.026) |  |  |
|  |  |  | **65-75** | d | 48 | 0.127 (0.098 - 0.165) |  |  | 0.020 (0.014 - 0.025) |  |  |
|  |  | **Gender** | **F** | a | 110 | 0.139 (0.109 - 0.165) | 0.292 | 0.124 | 0.022 (0.017 - 0.028) | 0.347 | 0.741 |
|  |  |  | **M** | b | 92 | 0.141 (0.105 - 0.189) |  |  | 0.021 (0.014 - 0.028) |  |  |
|  |  | **BMI classes** | **< 25** | a | 62 | 0.139 (0.109 - 0.183) | 0.830 | 0.717 | 0.021 (0.016 - 0.027) | 0.931 | 0.964 |
|  |  |  | **25 to < 30** | b | 90 | 0.139 (0.105 - 0.171) |  |  | 0.022 (0.015 - 0.028) |  |  |
|  |  |  | **>= 30** | c | 50 | 0.149 (0.105 - 0.177) |  |  | 0.021 (0.017 - 0.028) |  |  |
|  | **Belgium** | **Age Group (y)** | **35-44** | a | 30 | 0.098 (0.074 - 0.129) | 0.103 | 0.198 | 0.019 (0.017 - 0.022) | 0.090 | 0.290 |
|  |  |  | **45-54** | b | 65 | 0.117 (0.082 - 0.139) |  |  | 0.017 (0.015 - 0.022) |  |  |
|  |  |  | **55-64** | c | 72 | 0.121 (0.083 - 0.151) |  |  | 0.019 (0.015 - 0.028) |  |  |
|  |  |  | **65-75** | d | 74 | 0.102 (0.072 - 0.129) |  |  | 0.016 (0.013 - 0.022) |  |  |
|  |  | **Gender** | **F** | a | 130 | 0.113 (0.077 - 0.144) | 0.825 | 0.995 | 0.018 (0.015 - 0.024) | 0.139 | 0.287 |
|  |  |  | **M** | b | 111 | 0.105 (0.074 - 0.134) |  |  | 0.017 (0.014 - 0.024) |  |  |
|  |  | **BMI classes** | **< 25** | a | 125 | 0.105 (0.074 - 0.139) | 0.595 | 0.445 | 0.017 (0.014 - 0.022) | 0.163 | 0.165 |
|  |  |  | **25 to < 30** | b | 85 | 0.113 (0.085 - 0.144) |  |  | 0.017 (0.014 - 0.022) |  |  |
|  |  |  | **>= 30** | c | 31 | 0.113 (0.069 - 0.139) |  |  | 0.022 (0.016 - 0.026) |  |  |
|  | **Germany** | **Age Group (y)** | **35-44** | a | 60 | 0.129 (0.095 - 0.159) | 0.894 | 0.947 | 0.021 (0.015 - 0.027) | 0.297 | 0.442 |
|  |  |  | **45-54** | b | 90 | 0.132 (0.105 - 0.154) |  |  | 0.021 (0.017 - 0.029) |  |  |
|  |  |  | **55-64** | c | 90 | 0.119 (0.098 - 0.171) |  |  | 0.020 (0.016 - 0.026) |  |  |
|  |  |  | **65-75** | d | 86 | 0.127 (0.109 - 0.154) |  |  | 0.019 (0.015 - 0.025) |  |  |
|  |  | **Gender** | **F** | a | 182 | 0.129 (0.105 - 0.154) | 0.521 | 0.776 | 0.021 (0.017 - 0.027) | 0.117 | 0.017 |
|  |  |  | **M** | b | 144 | 0.125 (0.095 - 0.165) |  |  | 0.020 (0.015 - 0.027) |  |  |
|  |  | **BMI classes** | **< 25** | a | 180 | 0.125 (0.102 - 0.154) | 0.065 | 0.148 | 0.020 (0.016 - 0.027) | 0.796 | 0.530 |
|  |  |  | **25 to < 30** | b | 120 | 0.121 (0.095 - 0.162) |  |  | 0.019 (0.016 - 0.027) |  |  |
|  |  |  | **>= 30** | c | 26 | 0.139 (0.129 - 0.183) |  |  | 0.020 (0.016 - 0.029) |  |  |

^1^ KW test: non parametric comparison by the Kruskal-Wallis test of *DNMT1* and *DNMT3B* mRNAs levels (data for two group comparison are analysed with the Mann-Whitney U test); data are reported as median and interquartile range (IQ). Pairwise comparisons are referred to the KW test and adjusted for multiple comparisons (comparisons with p < 0.05 are marked by the associated superscripts).

^2^ GLM: comparison by generalized linear models of *DNMT1* (gamma distribution with log-link function) and *DNMT3B* (linear model with log-transformed values and identity link-function) mRNAs levels. All GLM models included the effects of age-groups, gender and age. Definition of abbreviations is provided in the supplementary list.

**Supplementary Table 3. Influence of dietary habits on *DNMT1* and *DNMT3B* expression in the RASIG population.**

|  |  |  |  | *DNMT1* mRNA | | | *DNMT3B* mRNA | | |
| --- | --- | --- | --- | --- | --- | --- | --- | --- | --- |
| Variable | **Frequency** | **Stat** | **N** | **Median (IQ)** | **p (KW)^1^** | **p (GLM)^2^** | **Median (IQ)** | **p (KW)^1^** | **p (GLM)^2^** |
| Vegetables | **< 1 serv./day** | a | 678 | 0.125 (0.095 - 0.165) | 0.172 | 0.961 | 0.021 (0.016 - 0.029) | 0.114 | 0.231 |
|  | **= 1 serv./day** | b | 794 | 0.125 (0.095 - 0.165) |  |  | 0.020 (0.015 - 0.027) |  |  |
|  | **≥ 2 serv./day** | c | 302 | 0.134 (0.102 - 0.171) |  |  | 0.021 (0.016 - 0.027) |  |  |
| Fruit | **< 1 serv./day** | a | 610 | 0.125 (0.092 - 0.165) | 0.212 | 0.745 | 0.021 (0.015 - 0.028) | 0.955 | 0.572 |
|  | **= 1 serv./day** | b | 695 | 0.125 (0.095 - 0.171) |  |  | 0.021 (0.016 - 0.028) |  |  |
|  | **≥ 2 serv./day** | c | 469 | 0.129 (0.102 - 0.165) |  |  | 0.021 (0.015 - 0.027) |  |  |
| Meat | **≤ 1 serv./wk** | a | 180 | 0.129 (0.103 - 0.165) | 0.351 | 0.529 | 0.020 (0.015 - 0.028) | 0.620 | 0.176 |
|  | **2-6 serv./wk** | b | 1391 | 0.125 (0.095 - 0.165) |  |  | 0.021 (0.016 - 0.028) |  |  |
|  | **≥ 7 serv./wk** | c | 203 | 0.129 (0.098 - 0.165) |  |  | 0.021 (0.015 - 0.028) |  |  |
| Vitamin | **No serv./day** | a | 1124 | 0.129 (0.095 - 0.165) | 0.856 | 0.912 | 0.021 (0.015 - 0.028) | 0.763 | 0.687 |
|  | **< 1 serv./day** | b | 412 | 0.129 (0.095 - 0.165) |  |  | 0.021 (0.016 - 0.028) |  |  |
|  | **≥ 1 serv./day** | c | 238 | 0.129 (0.098 - 0.159) |  |  | 0.020 (0.015 - 0.027) |  |  |
| Dairy Products | **< 1 serv./wk or never** | a | 219 | 0.134 (0.105 - 0.177)^a^ | 0.040 | 0.080 | 0.021 (0.016 - 0.028) | 0.709 | 0.386 |
|  | **= 1-6 serv./wk** | b | 581 | 0.129 (0.095 - 0.165) |  |  | 0.021 (0.015 - 0.027) |  |  |
|  | **≥ 1 serv./day** | c | 974 | 0.125 (0.092 - 0.165)^c^ |  |  | 0.021 (0.015 - 0.028) |  |  |
| Eggs | **< 1 serv./wk or never** | a | 788 | 0.129 (0.098 - 0.171) | 0.133 | 0.302 | 0.021 (0.016 - 0.028) | 0.222 | 0.202 |
|  | **≥ 1 serv./wk** | b | 986 | 0.125 (0.092 - 0.165) |  |  | 0.021 (0.015 - 0.027) |  |  |
| Fish | **< 1 serv./wk or never** | a | 797 | 0.129 (0.098 - 0.165) | 0.409 | 0.584 | 0.021 (0.016 - 0.028) | 0.557 | 0.757 |
|  | **≥ 1 serv./wk** | b | 977 | 0.125 (0.095 - 0.171) |  |  | 0.021 (0.015 - 0.028) |  |  |
| French fries | **never** | a | 479 | 0.134 (0.105 - 0.171)^b.c^ | 0.002 | 0.320 | 0.021 (0.016 - 0.027) | 0.333 | 0.934 |
|  | **< 1 serv./wk** | b | 1009 | 0.125 (0.092 - 0.165)^a^ |  |  | 0.021 (0.015 - 0.028) |  |  |
|  | **≥1 serv./wk** | c | 286 | 0.125 (0.088 - 0.159)^a^ |  |  | 0.020 (0.015 - 0.027) |  |  |
| Brown bread | **< 1 serv./wk or never** | a | 925 | 0.134 (0.098 - 0.171)^b.c^ | < 0.001 | 0.544 | 0.021 (0.015 - 0.027) | 0.052 | 0.157 |
|  | **= 1-6 serv./wk** | b | 475 | 0.125 (0.095 - 0.159)^a^ |  |  | 0.020 (0.015 - 0.028) |  |  |
|  | **≥ 7 serv./wk** | c | 374 | 0.121 (0.085 - 0.154)^a^ |  |  | 0.021 (0.016 - 0.029) |  |  |
| White bread | **< 1 serv./wk or never** | a | 661 | 0.125 (0.095 - 0.165)^c^ | < 0.001 | 0.019 | 0.021 (0.016 - 0.029) | 0.489 | 0.069 |
|  | **= 1-6 serv./wk** | b | 613 | 0.125 (0.092 - 0.154)^c^ |  |  | 0.021 (0.015 - 0.028) |  |  |
|  | **≥ 7 serv./wk** | c | 500 | 0.134 (0.102 - 0.177)^a.b^ |  |  | 0.021 (0.016 - 0.027) |  |  |
| Whole bread | **< 1 serv./wk or never** | a | 729 | 0.129 (0.098 - 0.171) | 0.092 | 0.840 | 0.020 (0.015 - 0.027) | 0.178 | 0.845 |
|  | **= 1-6 serv./wk** | b | 613 | 0.125 (0.092 - 0.165) |  |  | 0.021 (0.016 - 0.028) |  |  |
|  | **≥ 7 serv./wk** | c | 432 | 0.125 (0.095 - 0.165) |  |  | 0.021 (0.015 - 0.028) |  |  |
| Smoking habits | **never** | a | 933 | 0.125 (0.095 - 0.165) | 0.883 | 0.528 | 0.021 (0.015 - 0.028) | 0.831 | 0.138 |
|  | **former** | b | 545 | 0.129 (0.098 - 0.165) |  |  | 0.021 (0.016 - 0.028) |  |  |
|  | **current** | c | 296 | 0.129 (0.095 - 0.171) |  |  | 0.021 (0.015 - 0.027) |  |  |
| Alcohol | **< 1 serv./day** | a | 1227 | 0.129 (0.098 - 0.165)^c^ | 0.040 | 0.524 | 0.021 (0.016 - 0.028) | 0.148 | 0.667 |
|  | **= 1 serv./day** | b | 204 | 0.129 (0.092 - 0.171) |  |  | 0.020 (0.015 - 0.029) |  |  |
|  | **> 1 serv./day** | c | 343 | 0.121 (0.085 - 0.165)^a^ |  |  | 0.020 (0.015 - 0.027) |  |  |

^1^ KW test: non parametric comparison by the Kruskal-Wallis test of *DNMT1* and *DNMT3B* mRNAs levels (data for two group comparison are analysed with the Mann-Whitney U test); Data are reported as median and interquartile range (IQ). Pairwise comparisons are referred to the KW test and adjusted for multiple comparisons (comparisons with p < 0.05 are marked by the associated superscripts).

^2^ GLM: comparison by generalized linear models of *DNMT1* (gamma distribution with log-link function model) and *DNMT3B* (linear model with log-transformed values) mRNAs levels. All GLM models included the effects of gender, recruitment centre and age (continuous variable) as covariate.

**Supplementary Table 4. Influence of cardiovascular and diabetes risk biomarkers on *DNMT1* and *DNMT3B* expression in the RASIG population.**

|  | |  |  | *DNMT1* mRNA | | | *DNMT3B* mRNA | | |
| --- | --- | --- | --- | --- | --- | --- | --- | --- | --- |
| Variable | | **Stat** | **N** | **Median (IQ)** | **p (KW)^1^** | **p (GLM)^2^** |  | **p (KW)^1^** | **p (GLM)^2^** |
| Serum Glucose (Quartiles) | ≤ 4.65 mmol/L | a | 444 | 0.121 (0.088 - 0.159)^c^ | 0.005 | 0.869 | 0.020 (0.015 - 0.028) | 0.673 | 0.478 |
|  | 4.65 – 5.09 mmol/L | b | 437 | 0.125 (0.095 - 0.159) |  |  | 0.021 (0.015 - 0.029) |  |  |
|  | 5.09 – 5.58 mmol/L | c | 418 | 0.134 (0.102 - 0.171)^a^ |  |  | 0.021 (0.015 - 0.028) |  |  |
|  | ≥ 5.58 mmol/L | d | 359 | 0.129 (0.102 - 0.165) |  |  | 0.021 (0.016 - 0.026) |  |  |
| Glycosylated Haemoglobin  nA1C (Quartiles) | ≤ 5.64 % | a | 479 | 0.125 (0.085 - 0.159) | 0.051 | 0.429 | 0.021 (0.016 - 0.028) | 0.111 | 0.067 |
|  | 5.65 – 5.95 % | b | 452 | 0.129 (0.095 - 0.165) |  |  | 0.021 (0.016 - 0.029) |  |  |
|  | 5.96 – 6.29 % | c | 427 | 0.129 (0.102 - 0.171) |  |  | 0.021 (0.015 - 0.028) |  |  |
|  | ≥ 6.30 % | d | 401 | 0.129 (0.098 - 0.171) |  |  | 0.020 (0.015 - 0.026) |  |  |
| Cholesterol (Tertiles) | ≤ 220 mg/dl | a | 515 | 0.129 (0.098 - 0.165) | 0.485 | 0.595 | 0.021 (0.015 - 0.028) | 0.667 | 0.698 |
|  | 220 – 257 mg/dl | b | 551 | 0.125 (0.088 - 0.165) |  |  | 0.021 (0.015 - 0.027) |  |  |
|  | > 257 mg/dl | c | 562 | 0.129 (0.098 - 0.171) |  |  | 0.021 (0.016 - 0.028) |  |  |
| Triglycerides (Quartiles) | < 0.78 mmol/L | a | 429 | 0.125 (0.092 - 0.159) | 0.406 | 0.494 | 0.021 (0.016 - 0.028) | 0.991 | 0.588 |
|  | 0.78 – 1.05 mmol/L | b | 400 | 0.129 (0.095 - 0.165) |  |  | 0.021 (0.015 - 0.028) |  |  |
|  | 1.05 – 1.51 mmol/L | c | 402 | 0.129 (0.098 - 0.165) |  |  | 0.021 (0.015 - 0.027) |  |  |
|  | ≥ 1.51 mmol/L | d | 427 | 0.129 (0.095 - 0.165) |  |  | 0.021 (0.016 - 0.028) |  |  |
| HDL (Quartiles) | ≤ 1.20 mmol/L | a | 428 | 0.127 (0.095 - 0.165) | 0.202 | 0.473 | 0.020 (0.015 - 0.027) | 0.050 | 0.421 |
|  | 1.21 – 1.47 mmol/L | b | 402 | 0.125 (0.095 - 0.171) |  |  | 0.020 (0.015 - 0.027) |  |  |
|  | 1.48 – 1.80 mmol/L | c | 413 | 0.129 (0.098 - 0.171) |  |  | 0.021 (0.016 - 0.028) |  |  |
|  | > 1.80 mmol/L | d | 414 | 0.125 (0.092 - 0.159) |  |  | 0.021 (0.016 - 0.030) |  |  |
| LDL (Quartiles) | ≤ 2.70 mmol/L | a | 419 | 0.125 (0.095 - 0.159) | 0.454 | 0.320 | 0.021 (0.015 - 0.028) | 0.832 | 0.387 |
|  | 2.71 – 3.29 mmol/L | b | 423 | 0.125 (0.095 - 0.159) |  |  | 0.021 (0.015 - 0.027) |  |  |
|  | 3.30 – 3.85 mmol/L | c | 410 | 0.129 (0.098 - 0.177) |  |  | 0.020 (0.016 - 0.027) |  |  |
|  | ≥ 3.86 mmol/L | d | 406 | 0.129 (0.095 - 0.165) |  |  | 0.021 (0.015 - 0.029) |  |  |
| Free fatty Acids (Quartiles) | < 0.45 mg/dl | a | 421 | 0.125 (0.095 - 0.159) | 0.449 | 0.506 | 0.020 (0.015 - 0.027) | 0.495 | 0.611 |
|  | 0.45 – 0.60 mg/dl | b | 411 | 0.134 (0.095 - 0.165) |  |  | 0.021 (0.016 - 0.028) |  |  |
|  | 0.60 – 0.80 mg/dl | c | 422 | 0.129 (0.095 - 0.171) |  |  | 0.021 (0.016 - 0.028) |  |  |
|  | ≥ 0.80 mg/dl | d | 402 | 0.125 (0.095 - 0.171) |  |  | 0.021 (0.015 - 0.028) |  |  |
| Homocysteine (Quartiles) | ≤ 11.05 µmol/L | a | 464 | 0.134 (0.105 - 0.177) | < 0.001 | 0.119 | 0.021 (0.016 - 0.028) | 0.350 | 0.926 |
|  | 11.06 – 14.22 µmol/L | b | 431 | 0.129 (0.092 - 0.159) |  |  | 0.021 (0.016 - 0.027) |  |  |
|  | 14.23 – 18.27 µmol/L | c | 438 | 0.125 (0.095 - 0.165) |  |  | 0.021 (0.015 - 0.029) |  |  |
|  | > 18.27 µmol/L | d | 440 | 0.121 (0.088 - 0.165) |  |  | 0.020 (0.015 - 0.027) |  |  |

^1^ KW test: non parametric comparison by the Kruskal-Wallis test of *DNMT1* and *DNMT3B* mRNAs levels (data for two group comparison are analysed with the Mann-Whitney U test); Data are reported as median and interquartile range (IQ). Pairwise comparisons are referred to the KW test and adjusted for multiple comparisons (comparisons with p < 0.05 are marked by the associated superscripts).

^2^ GLM: comparison by generalized linear models of *DNMT1* (gamma distribution with log-link function model) and *DNMT3B* (linear model with log-transformed values) mRNAs levels. All GLM models included the effects of gender, recruitment centre and age (continuous variable) as covariate. Definition of abbreviations is provided in the supplementary list.

**Supplementary Table 5. Influence of haematological parameters on *DNMT1* and *DNMT3B* expression in the RASIG population.**

|  |  |  |  | *DNMT1* mRNA |  |  | *DNMT3B* mRNA |  |  |
| --- | --- | --- | --- | --- | --- | --- | --- | --- | --- |
| Variable |  | **stat** | **N** | **Median (IQ)** | **p (KW)^1^** | **p (GLM)^2^** | **Median (IQ)** | **p (KW)^1^** | **p (GLM)^2^** |
| MCH (Quartiles) | ≤ 29.1 pg | a | 484 | 0.129 (0.102 - 0.171)^d^ | 0.070 | 0.286 | 0.021 (0.015 - 0.029) | 0.142 | 0.611 |
|  | 29.2 – 30.1 pg | b | 456 | 0.129 (0.095 - 0.165) |  |  | 0.021 (0.015 - 0.027) |  |  |
|  | 30.1 – 31.0 pg | c | 418 | 0.129 (0.095 - 0.165) |  |  | 0.021 (0.016 - 0.028) |  |  |
|  | > 31.0 pg | d | 391 | 0.121 (0.085 - 0.159)^a^ |  |  | 0.020 (0.015 - 0.027) |  |  |
| MCHC (Quartiles) | ≤ 32.8 g/dl | a | 547 | 0.125 (0.092 - 0.165) | 0.274 | 0.929 | 0.021 (0.016 - 0.030) | 0.037 | 0.376 |
|  | 32.8 – 33.5 g/dl | b | 392 | 0.125 (0.095 - 0.165) |  |  | 0.021 (0.016 - 0.029) |  |  |
|  | 33.5 – 34.2 g/dl | c | 429 | 0.129 (0.098 - 0.165) |  |  | 0.021 (0.015 - 0.026) |  |  |
|  | > 34.2 g/dl | d | 332 | 0.134 (0.098 - 0.171) |  |  | 0.019 (0.015 - 0.027) |  |  |
| MCV (Quartiles) | ≤ 86.7 fl | a | 477 | 0.139 (0.105 - 0.171)^b.c.d^ | < 0.001 | 0.239 | 0.021 (0.016 - 0.028) | 0.218 | 0.059 |
|  | 86.8 – 89.7 fl | b | 400 | 0.125 (0.095 - 0.165)^a^ |  |  | 0.021 (0.016 - 0.028) |  |  |
|  | 89.8 – 92.5 fl | c | 415 | 0.121 (0.088 - 0.154)^a^ |  |  | 0.020 (0.015 - 0.027) |  |  |
|  | > 92.5 fl | d | 457 | 0.125 (0.088 - 0.165)^a^ |  |  | 0.020 (0.015 - 0.028) |  |  |
| HCT (Quartiles) | ≤ 40.0 % | a | 465 | 0.129 (0.102 - 0.171)^d^ | 0.035 | 0.844 | 0.021 (0.016 - 0.028) | 0.051 | 0.585 |
|  | 40.1 – 42.1 % | b | 409 | 0.129 (0.095 - 0.165) |  |  | 0.021 (0.016 - 0.028) |  |  |
|  | 42.2 – 44.5 % | c | 424 | 0.129 (0.095 - 0.165) |  |  | 0.021 (0.016 - 0.028) |  |  |
|  | ≥ 44.6 % | d | 449 | 0.121 (0.088 - 0.159)^a^ |  |  | 0.020 (0.015 - 0.026) |  |  |
| RDW (Quartiles) | ≤ 12.7 % | a | 358 | 0.129 (0.095 - 0.171) | 0.676 | 0.320 | 0.021 (0.015 - 0.030) | 0.305 | 0.422 |
|  | 12.8 – 13.2 % | b | 376 | 0.125 (0.095 - 0.165) |  |  | 0.021 (0.016 - 0.028) |  |  |
|  | 13.3 – 13.6 % | c | 271 | 0.129 (0.095 - 0.171) |  |  | 0.020 (0.015 - 0.027) |  |  |
|  | ≥ 13.7 % | d | 336 | 0.129 (0.092 - 0.165) |  |  | 0.021 (0.015 - 0.026) |  |  |
| HGB (Quartiles) | < 13.4 g/dl | a | 466 | 0.129 (0.098 - 0.171) | 0.127 | 0.554 | 0.021 (0.016 - 0.029)^d^ | 0.018 | 0.622 |
|  | 13.4 – 14.2 g/dl | b | 440 | 0.129 (0.098 - 0.165) |  |  | 0.021 (0.016 - 0.029) |  |  |
|  | 14.2 – 15.0 g/dl | c | 439 | 0.125 (0.095 - 0.165) |  |  | 0.020 (0.015 - 0.027) |  |  |
|  | > 15.0 g/dl | d | 422 | 0.125 (0.092 - 0.165) |  |  | 0.020 (0.015 - 0.026)^a^ |  |  |
| RBC (Quartiles) | ≤ 4.45 Mio/µl | a | 441 | 0.125 (0.095 - 0.171) | 0.917 | 0.972 | 0.021 (0.016 - 0.029) | 0.452 | 0.955 |
|  | 4.46 – 4.70 Mio/µl | b | 435 | 0.129 (0.095 - 0.159) |  |  | 0.021 (0.016 - 0.028) |  |  |
|  | 4.71 – 5.0 Mio/µl | c | 419 | 0.129 (0.092 - 0.165) |  |  | 0.020 (0.015 - 0.028) |  |  |
|  | > 5.0 Mio/µl | d | 453 | 0.125 (0.098 - 0.165) |  |  | 0.020 (0.015 - 0.027) |  |  |
| White Blood cells (Quartiles) | < 5.00 Tsd/µl | a | 457 | 0.129 (0.095 - 0.171) | 0.189 | 0.014 | 0.021 (0.016 - 0.029) | 0.211 | 0.561 |
|  | 5.00 – 5.89 Tsd/µl | b | 411 | 0.125 (0.098 - 0.165) |  |  | 0.020 (0.015 - 0.027) |  |  |
|  | 5.90 – 6.91 Tsd/µl | c | 435 | 0.129 (0.098 - 0.171) |  |  | 0.021 (0.015 - 0.028) |  |  |
|  | ≥ 6.91 Tsd/µl | d | 460 | 0.125 (0.092 - 0.159) |  |  | 0.020 (0.016 - 0.027) |  |  |
| Monocytes (Quartiles) | < 364 n/µl | a | 426 | 0.134 (0.102 - 0.177)^c.d^ | < 0.001 | < 0.001 | 0.021 (0.016 - 0.029)^c.d^ | 0.004 | 0.031 |
|  | 364 – 452 n/µl | b | 440 | 0.129 (0.102 - 0.171)^d^ |  |  | 0.021 (0.016 - 0.029) |  |  |
|  | 452 – 566 n/µl | c | 437 | 0.125 (0.095 - 0.159)^a^ |  |  | 0.020 (0.015 - 0.027)^a^ |  |  |
|  | > 566 n/µl | d | 440 | 0.117 (0.088 - 0.154)^a.b^ |  |  | 0.020 (0.015 - 0.027)^a^ |  |  |
| Lymphocytes (Quartiles) | < 1540 n/µl | a | 449 | 0.121 (0.088 - 0.154)^d^ | < 0.001 | 0.050 | 0.020 (0.015 - 0.027) | 0.822 | 0.886 |
|  | 1540 – 1890 n/µl | b | 454 | 0.129 (0.095 - 0.165) |  |  | 0.021 (0.016 - 0.028) |  |  |
|  | 1891 – 2281 n/µl | c | 424 | 0.125 (0.095 - 0.159)^d^ |  |  | 0.021 (0.016 - 0.027) |  |  |
|  | > 2281 n/µl | d | 411 | 0.139 (0.102 - 0.177)^a.c^ |  |  | 0.021 (0.015 - 0.028) |  |  |
| Lymphocytes/Monocytes (Quartiles) | < 3.35 | a | 440 | 0.113 (0.082 - 0.144)^b.c.d^ | < 0.001 | < 0.001 | 0.020 (0.015 - 0.026)^c.d^ | 0.002 | 0.052 |
|  | 3.35-4.20 | b | 465 | 0.125 (0.092 - 0.165)^d^ |  |  | 0.020 (0.015 - 0.028) |  |  |
|  | 4.20-5.24 | c | 442 | 0.129 (0.102 - 0.165)^d^ |  |  | 0.021 (0.016 - 0.028)^a^ |  |  |
|  | > 5.24 | d | 391 | 0.144 (0.109 - 0.183)^a.b.c^ |  |  | 0.021 (0.016 - 0.030)^a^ |  |  |
| CD3+CD45+ Cells % (Quartiles) | < 70 % | a | 340 | 0.125 (0.095 - 0.159)^d^ | < 0.001 | 0.002 | 0.019 (0.015 - 0.026)^d^ | < 0.001 | 0.001 |
|  | 70-75 % | b | 283 | 0.134 (0.105 - 0.171) |  |  | 0.021 (0.015 - 0.026)^d^ |  |  |
|  | 75-80 % | c | 303 | 0.134 (0.105 - 0.177) |  |  | 0.021 (0.015 - 0.027) |  |  |
|  | > 80 % | d | 331 | 0.144 (0.109 - 0.189)^a^ |  |  | 0.022 (0.017 - 0.031)^a.b^ |  |  |
| Neutrophils (Quartiles) | < 2.56 Tsd/µl | a | 423 | 0.139 (0.102 - 0.177)^d^ | < 0.001 | < 0.001 | 0.021 (0.016 - 0.029) | 0.158 | 0.442 |
|  | 2.56 – 3.20 Tsd/µl | b | 408 | 0.125 (0.098 - 0.165) |  |  | 0.021 (0.015 - 0.028) |  |  |
|  | 3.20 – 3.99 Tsd/µl | c | 453 | 0.129 (0.095 - 0.171)^d^ |  |  | 0.021 (0.015 - 0.027) |  |  |
|  | ≥ 3.99 Tsd/µl | d | 460 | 0.121 (0.088 - 0.154)^a.c^ |  |  | 0.020 (0.015 - 0.027) |  |  |
| Eosinophils (Quartiles) | ≤ 100 n/µl | a | 444 | 0.129 (0.096 - 0.171)^d^ | < 0.001 | 0.438 | 0.021 (0.015 - 0.028) | 0.158 | 0.157 |
|  | 100 – 151 n/µl | b | 439 | 0.129 (0.098 - 0.165) |  |  | 0.020 (0.015 - 0.026) |  |  |
|  | 151 – 231 n/µl | c | 443 | 0.125 (0.095 - 0.165)^d^ |  |  | 0.021 (0.015 - 0.028) |  |  |
|  | ≥ 231 n/µl | d | 416 | 0.125 (0.092 - 0.165)^a.c^ |  |  | 0.021 (0.016 - 0.029) |  |  |
| Basophils (Quartiles) | ≤ 20.4 n/µl | a | 391 | 0.129 (0.098 - 0.171) | 0.197 | 0.351 | 0.021 (0.016 - 0.028) | 0.218 | 0.543 |
|  | 20.5 – 32.4 n/µl | b | 448 | 0.125 (0.102 - 0.159) |  |  | 0.020 (0.015 - 0.027) |  |  |
|  | 32.4 – 48.8 n/µl | c | 445 | 0.129 (0.095 - 0.171) |  |  | 0.020 (0.015 - 0.027) |  |  |
|  | > 48.8 n/µl | d | 457 | 0.125 (0.088 - 0.165) |  |  | 0.021 (0.016 - 0.028) |  |  |
| Platelets (Quartiles) | ≤ 197 Tsd/µl | a | 392 | 0.129 (0.092 - 0.171) | 0.951 | 0.748 | 0.021 (0.015 - 0.028) | 0.008 | 0.032 |
|  | 197 – 229 Tsd/µl | b | 436 | 0.125 (0.095 - 0.165) |  |  | 0.019 (0.015 - 0.026)^d^ |  |  |
|  | 230 – 268 Tsd/µl | c | 429 | 0.129 (0.102 - 0.165) |  |  | 0.021 (0.015 - 0.028) |  |  |
|  | ≥ 269 Tsd/µl | d | 501 | 0.129 (0.095 - 0.165) |  |  | 0.021 (0.016 - 0.029)^b^ |  |  |
| MPV (Quartiles) | ≤ 8.7 fl | a | 408 | 0.125 (0.095 - 0.171) | 0.250 | 0.986 | 0.021 (0.015 - 0.030) | 0.290 | 0.833 |
|  | 8.7 – 9.6 fl | b | 318 | 0.134 (0.095 - 0.171) |  |  | 0.021 (0.015 - 0.028) |  |  |
|  | 9.7 – 10.7 fl | c | 319 | 0.125 (0.092 - 0.165) |  |  | 0.020 (0.015 - 0.027) |  |  |
|  | ≥ 10.8 fl | d | 279 | 0.125 (0.088 - 0.159) |  |  | 0.020 (0.015 - 0.027) |  |  |

^1^ KW test: non parametric comparison by the Kruskal-Wallis test of *DNMT1* and *DNMT3B* mRNAs levels (data for two group comparison are analysed with the Mann-Whitney U test); Data are reported as median and interquartile range (IQ). Pairwise comparisons are referred to the KW test and adjusted for multiple comparisons (comparisons with p < 0.05 are marked by the associated superscripts).

^2^ GLM: comparison by generalized linear models of *DNMT1* (gamma distribution with log-link function model) and *DNMT3B* (linear model with log-transformed values) mRNAs levels. All GLM models included the effects of gender, recruitment centre and age (continuous variable) as covariate.

Definition of abbreviations is provided in the supplementary list.

**Supplementary Table 6. Effect of Age, group, demographic characteristics, dietary habits, cardiovascular risk factors and haematological parameters on *DNMT1* and *DNMT3B* expression in the whole population.**

|  |  |  |  | *DNMT1* mRNA |  | *DNMT3B* mRNA |  |
| --- | --- | --- | --- | --- | --- | --- | --- |
| Variable |  | **Stat** | **N** | **Median (IQ)** | **p (KW)^1^** | **Median (IQ)** | **p (KW)^1^** |
| Age Group (y) | 35-44 | a | 383 | 0.134 (0.102 - 0.171)^c^ | < 0.001 | 0.022 (0.017 - 0.029)^c.d^ | 0.002 |
|  | 45-54 | b | 484 | 0.129 (0.095 - 0.159) |  | 0.021 (0.016 - 0.029) |  |
|  | 55-64 | c | 807 | 0.117 (0.085 - 0.159)^a^ |  | 0.020 (0.015 - 0.027)^a^ |  |
|  | 65-75 | d | 779 | 0.125 (0.092 - 0.165) |  | 0.020 (0.015 - 0.026)^a^ |  |
| Group | GO | a | 437 | 0.117 (0.082 - 0.154)^b^ | < 0.001 | 0.020 (0.015 - 0.028) | 0.152 |
|  | RASIG | b | 1774 | 0.129 (0.095 - 0.165)^a.c^ |  | 0.021 (0.015 - 0.028) |  |
|  | SGO | c | 242 | 0.115 (0.088 - 0.154)^b^ |  | 0.019 (0.014 - 0.027) |  |
| Centre | Finland | a | 249 | 0.105 (0.074 - 0.144)^b.d.e.f.h^ | < 0.001 | 0.023 (0.017 - 0.030)^b.e.f.g^ | < 0.001 |
|  | Italy | b | 485 | 0.144 (0.109 - 0.183)^a.c.d.e.g.h^ |  | 0.021 (0.016 - 0.027)^a.f.g^ |  |
|  | Austria | c | 267 | 0.113 (0.080 - 0.165)^b.f^ |  | 0.023 (0.015 - 0.035)^e.f.g^ |  |
|  | Greece | d | 317 | 0.125 (0.088 - 0.171)^a.b.g^ |  | 0.021 (0.016 - 0.031)^f.g^ |  |
|  | Poland | e | 270 | 0.129 (0.092 - 0.165)^a.b.g^ |  | 0.021 (0.014 - 0.027)^a.c^ |  |
|  | The Netherlands | f | 189 | 0.139 (0.105 - 0.165)^a.c.g^ |  | 0.018 (0.014 - 0.023)^a.b.c.d.h^ |  |
|  | Belgium | g | 350 | 0.105 (0.077 - 0.134)^b.d.e.f.h^ |  | 0.018 (0.014 - 0.025)^a.b.c.d.h^ |  |
|  | Germany | h | 326 | 0.129 (0.102 - 0.159)^a.b.g^ |  | 0.020 (0.016 - 0.027)^f.g^ |  |
| Gender | F | a | 1308 | 0.125 (0.095 - 0.165) | 0.110 | 0.021 (0.016 - 0.029)^b^ | < 0.001 |
|  | M | b | 1145 | 0.121 (0.092 - 0.165) |  | 0.020 (0.015 - 0.026)^a^ |  |
| BMI classes | < 25 | a | 1059 | 0.121 (0.092 - 0.159) | 0.107 | 0.020 (0.015 - 0.028) | 0.126 |
|  | 25 to < 30 | b | 957 | 0.125 (0.092 - 0.165) |  | 0.020 (0.015 - 0.027) |  |
|  | >= 30 | c | 436 | 0.129 (0.098 - 0.165) |  | 0.021 (0.016 - 0.028) |  |
| Vegetables | < 1 serv./day | a | 807 | 0.125 (0.092 - 0.165) | 0.007 | 0.021 (0.016 - 0.029) | 0.038 |
|  | = 1 serv./day | b | 1221 | 0.121 (0.092 - 0.159)^c^ |  | 0.020 (0.015 - 0.027) |  |
|  | ≥ 2 serv./day | c | 425 | 0.134 (0.098 - 0.171)^b^ |  | 0.021 (0.016 - 0.028) |  |
| Fruit | < 1 serv./day | a | 769 | 0.125 (0.092 - 0.159)^c^ | 0.011 | 0.021 (0.015 - 0.028) | 0.376 |
|  | = 1 serv./day | b | 1000 | 0.121 (0.088 - 0.159)^c^ |  | 0.020 (0.015 - 0.028) |  |
|  | ≥ 2 serv./day | c | 684 | 0.129 (0.098 - 0.165)^a.b^ |  | 0.021 (0.016 - 0.028) |  |
| Meat | ≤ 1 serv./wk | a | 224 | 0.129 (0.102 - 0.168) | 0.246 | 0.021 (0.015 - 0.028) | 0.454 |
|  | 2-6 serv./wk | b | 1847 | 0.125 (0.092 - 0.165) |  | 0.021 (0.015 - 0.028) |  |
|  | ≥ 7 serv./wk | c | 382 | 0.125 (0.095 - 0.159) |  | 0.020 (0.015 - 0.028) |  |
| Vitamin | No serv./day | a | 1534 | 0.125 (0.092 - 0.165) | 0.896 | 0.020 (0.015 - 0.028) | 0.417 |
|  | < 1 serv./day | b | 539 | 0.125 (0.092 - 0.165) |  | 0.021 (0.015 - 0.028) |  |
|  | ≥ 1 serv./day | c | 380 | 0.125 (0.095 - 0.157) |  | 0.021 (0.016 - 0.028) |  |
| Dairy Products | < 1 serv./wk or never | a | 308 | 0.129 (0.102 - 0.171)^c^ | 0.032 | 0.021 (0.016 - 0.028) | 0.424 |
|  | = 1-6 serv./wk | b | 725 | 0.125 (0.092 - 0.165) |  | 0.020 (0.015 - 0.027) |  |
|  | ≥ 1 serv./day | c | 1420 | 0.121 (0.092 - 0.159)^a^ |  | 0.021 (0.015 - 0.028) |  |
| Eggs | < 1 serv./wk or never | a | 1044 | 0.125 (0.095 - 0.165) | 0.103 | 0.021 (0.016 - 0.029)^b^ | 0.001 |
|  | ≥ 1 serv./wk | b | 1409 | 0.125 (0.092 - 0.159) |  | 0.020 (0.015 - 0.027)^a^ |  |
| Fish | < 1 serv./wk or never | a | 1016 | 0.125 (0.095 - 0.165) | 0.119 | 0.020 (0.015 - 0.028) | 0.675 |
|  | ≥ 1 serv./wk | b | 1437 | 0.125 (0.092 - 0.165) |  | 0.021 (0.015 - 0.028) |  |
| French fries | never | a | 644 | 0.129 (0.102 - 0.171) | 0.002 | 0.021 (0.016 - 0.028)^c^ | 0.009 |
|  | < 1 serv./wk | b | 1372 | 0.121 (0.092 - 0.159)^c^ |  | 0.020 (0.015 - 0.028) |  |
|  | ≥1 serv./wk | c | 437 | 0.121 (0.088 - 0.159)^b^ |  | 0.019 (0.015 - 0.026)^a^ |  |
| Brown bread | < 1 serv./wk or never | a | 1207 | 0.129 (0.098 - 0.165)^b.c^ | < 0.001 | 0.021 (0.015 - 0.027) | 0.237 |
|  | = 1-6 serv./wk | b | 592 | 0.121 (0.092 - 0.159)^a^ |  | 0.020 (0.015 - 0.028) |  |
|  | ≥ 7 serv./wk | c | 654 | 0.119 (0.082 - 0.154)^a^ |  | 0.021 (0.016 - 0.028) |  |
| White bread | < 1 serv./wk or never | a | 1029 | 0.125 (0.092 - 0.165)^b.c^ | < 0.001 | 0.020 (0.015 - 0.028) | 0.292 |
|  | = 1-6 serv./wk | b | 743 | 0.121 (0.088 - 0.154)^a^ |  | 0.020 (0.015 - 0.028) |  |
|  | ≥ 7 serv./wk | c | 681 | 0.129 (0.095 - 0.171)^a^ |  | 0.021 (0.016 - 0.027) |  |
| Whole bread | < 1 serv./wk or never | a | 976 | 0.127 (0.095 - 0.165) | 0.087 | 0.020 (0.015 - 0.027) | 0.527 |
|  | = 1-6 serv./wk | b | 748 | 0.121 (0.088 - 0.159) |  | 0.021 (0.015 - 0.028) |  |
|  | ≥ 7 serv./wk | c | 729 | 0.125 (0.092 - 0.159) |  | 0.020 (0.015 - 0.028) |  |
| Smoking habits | never | a | 1264 | 0.125 (0.092 - 0.165) | 0.833 | 0.021 (0.015 - 0.028) | 0.315 |
|  | former | b | 826 | 0.125 (0.095 - 0.165) |  | 0.020 (0.015 - 0.028) |  |
|  | current | c | 363 | 0.125 (0.088 - 0.165) |  | 0.020 (0.015 - 0.027) |  |
| Alcohol | < 1 serv./day | a | 1633 | 0.125 (0.095 - 0.165)^c^ | 0.020 | 0.021 (0.016 - 0.028) | 0.093 |
|  | = 1 serv./day | b | 287 | 0.125 (0.092 - 0.165) |  | 0.019 (0.015 - 0.027) |  |
|  | > 1 serv./day | c | 533 | 0.117 (0.085 - 0.159)^a^ |  | 0.020 (0.015 - 0.027) |  |
| Serum Glucose (Quartiles) | ≤ 4.65 mmol/L | a | 584 | 0.117 (0.084 - 0.154)^c.d^ | < 0.001 | 0.020 (0.015 - 0.027) | 0.101 |
|  | 4.65 – 5.09 mmol/L | b | 575 | 0.125 (0.092 - 0.159) |  | 0.021 (0.015 - 0.028) |  |
|  | 5.09 – 5.58 mmol/L | c | 578 | 0.129 (0.098 - 0.171)^a^ |  | 0.021 (0.016 - 0.028) |  |
|  | ≥ 5.58 mmol/L | d | 579 | 0.125 (0.095 - 0.165)^a^ |  | 0.021 (0.016 - 0.027) |  |
| Glycosylated Haemoglobin  nA1C (Quartiles) | ≤ 5.64 % | a | 606 | 0.117 (0.082 - 0.154)^c.d^ | 0.005 | 0.021 (0.016 - 0.028) | 0.050 |
|  | 5.65 – 5.95 % | b | 607 | 0.125 (0.092 - 0.165) |  | 0.021 (0.016 - 0.029) |  |
|  | 5.96 – 6.29 % | c | 616 | 0.125 (0.098 - 0.165)^a^ |  | 0.021 (0.015 - 0.028) |  |
|  | ≥ 6.30 % | d | 603 | 0.125 (0.098 - 0.165)^a^ |  | 0.020 (0.015 - 0.026) |  |
| Cholesterol (Tertiles) | ≤ 220 mg/dl | a | 756 | 0.125 (0.095 - 0.165) | 0.240 | 0.021 (0.015 - 0.028) | 0.792 |
|  | 220 – 257 mg/dl | b | 756 | 0.125 (0.088 - 0.159) |  | 0.021 (0.015 - 0.028) |  |
|  | > 257 mg/dl | c | 756 | 0.129 (0.095 - 0.168) |  | 0.020 (0.016 - 0.028) |  |
| Triglycerides (Quartiles) | < 0.78 mmol/L | a | 576 | 0.123 (0.092 - 0.159) | 0.698 | 0.020 (0.015 - 0.028) | 0.961 |
|  | 0.78 – 1.05 mmol/L | b | 581 | 0.121 (0.092 - 0.159) |  | 0.021 (0.015 - 0.028) |  |
|  | 1.05 – 1.51 mmol/L | c | 580 | 0.125 (0.095 - 0.165) |  | 0.021 (0.015 - 0.028) |  |
|  | ≥ 1.51 mmol/L | d | 579 | 0.125 (0.092 - 0.165) |  | 0.021 (0.015 - 0.028) |  |
| HDL (Quartiles) | ≤ 1.20 mmol/L | a | 580 | 0.121 (0.092 - 0.159) | 0.100 | 0.020 (0.014 - 0.027) | 0.022 |
|  | 1.21 – 1.47 mmol/L | b | 575 | 0.125 (0.095 - 0.171) |  | 0.020 (0.015 - 0.027) |  |
|  | 1.48 – 1.80 mmol/L | c | 578 | 0.125 (0.095 - 0.165) |  | 0.021 (0.016 - 0.028) |  |
|  | > 1.80 mmol/L | d | 582 | 0.121 (0.088 - 0.159) |  | 0.021 (0.016 - 0.029) |  |
| LDL (Quartiles) | ≤ 2.70 mmol/L | a | 580 | 0.121 (0.092 - 0.159) | 0.239 | 0.020 (0.015 - 0.027) | 0.913 |
|  | 2.71 – 3.29 mmol/L | b | 572 | 0.121 (0.092 - 0.154) |  | 0.021 (0.015 - 0.028) |  |
|  | 3.30 – 3.85 mmol/L | c | 583 | 0.125 (0.095 - 0.171) |  | 0.020 (0.015 - 0.027) |  |
|  | ≥ 3.86 mmol/L | d | 581 | 0.129 (0.092 - 0.165) |  | 0.021 (0.015 - 0.029) |  |
| Free fatty Acids (Quartiles) | < 0.45 mg/dl | a | 571 | 0.125 (0.095 - 0.159) | 0.565 | 0.020 (0.015 - 0.027) | 0.369 |
|  | 0.45 – 0.60 mg/dl | b | 585 | 0.125 (0.095 - 0.165) |  | 0.021 (0.016 - 0.028) |  |
|  | 0.60 – 0.80 mg/dl | c | 577 | 0.125 (0.092 - 0.165) |  | 0.021 (0.016 - 0.028) |  |
|  | ≥ 0.80 mg/dl | d | 579 | 0.125 (0.088 - 0.165) |  | 0.021 (0.015 - 0.028) |  |
| Homocysteine (Quartiles) | ≤ 11.05 µmol/L | a | 613 | 0.129 (0.098 - 0.171)^d^ | < 0.001 | 0.021 (0.016 - 0.028)^d^ | 0.006 |
|  | 11.06 – 14.22 µmol/L | b | 611 | 0.125 (0.092 - 0.159)^d^ |  | 0.021 (0.016 - 0.028)^d^ |  |
|  | 14.23 – 18.27 µmol/L | c | 613 | 0.125 (0.095 - 0.165)^d^ |  | 0.021 (0.015 - 0.028) |  |
|  | > 18.27 µmol/L | d | 613 | 0.113 (0.085 - 0.154)^a.b.c^ |  | 0.019 (0.014 - 0.027)^a.b^ |  |
| MCH (Quartiles) | ≤ 29.1 pg | a | 610 | 0.129 (0.098 - 0.165)^d^ | < 0.001 | 0.021 (0.015 - 0.029)^d^ | 0.025 |
|  | 29.2 – 30.1 pg | b | 606 | 0.125 (0.095 - 0.159)^d^ |  | 0.020 (0.015 - 0.027) |  |
|  | 30.1 – 31.0 pg | c | 600 | 0.125 (0.095 - 0.165)^d^ |  | 0.021 (0.016 - 0.028) |  |
|  | > 31.0 pg | d | 601 | 0.113 (0.082 - 0.154)^a.b.c^ |  | 0.020 (0.015 - 0.026)^a^ |  |
| MCHC (Quartiles) | ≤ 32.8 g/dl | a | 608 | 0.125 (0.092 - 0.159) | 0.659 | 0.021 (0.015 - 0.029) | 0.109 |
|  | 32.8 – 33.5 g/dl | b | 504 | 0.121 (0.092 - 0.159) |  | 0.021 (0.015 - 0.028) |  |
|  | 33.5 – 34.2 g/dl | c | 596 | 0.125 (0.095 - 0.162) |  | 0.020 (0.015 - 0.027) |  |
|  | > 34.2 g/dl | d | 574 | 0.125 (0.092 - 0.165) |  | 0.019 (0.015 - 0.027) |  |
| MCV (Quartiles) | ≤ 86.7 fl | a | 635 | 0.134 (0.102 - 0.171)^d^ | < 0.001 | 0.021 (0.016 - 0.028) | 0.055 |
|  | 86.8 – 89.7 fl | b | 604 | 0.125 (0.092 - 0.165)^d^ |  | 0.021 (0.016 - 0.028) |  |
|  | 89.8 – 92.5 fl | c | 579 | 0.121 (0.088 - 0.154)^d^ |  | 0.020 (0.015 - 0.027) |  |
|  | > 92.5 fl | d | 598 | 0.121 (0.082 - 0.154)^a.b.c^ |  | 0.020 (0.015 - 0.027) |  |
| HCT (Quartiles) | ≤ 40.0 % | a | 630 | 0.125 (0.095 - 0.165) | 0.105 | 0.021 (0.015 - 0.028) | 0.148 |
|  | 40.1 – 42.1 % | b | 567 | 0.125 (0.088 - 0.165) |  | 0.021 (0.015 - 0.028) |  |
|  | 42.2 – 44.5 % | c | 586 | 0.129 (0.095 - 0.165) |  | 0.020 (0.015 - 0.028) |  |
|  | ≥ 44.6 % | d | 579 | 0.121 (0.088 - 0.159) |  | 0.020 (0.015 - 0.027) |  |
| RDW (Quartiles) | ≤ 12.7 % | a | 406 | 0.125 (0.095 - 0.171) | 0.467 | 0.021 (0.015 - 0.031) | 0.071 |
|  | 12.8 – 13.2 % | b | 445 | 0.125 (0.088 - 0.159) |  | 0.021 (0.016 - 0.028) |  |
|  | 13.3 – 13.6 % | c | 366 | 0.129 (0.088 - 0.171) |  | 0.020 (0.015 - 0.027) |  |
|  | ≥ 13.7 % | d | 440 | 0.125 (0.092 - 0.159) |  | 0.020 (0.015 - 0.026) |  |
| HGB (Quartiles) | < 13.4 g/dl | a | 579 | 0.125 (0.095 - 0.171) | 0.291 | 0.021 (0.015 - 0.028) | 0.105 |
|  | 13.4 – 14.2 g/dl | b | 605 | 0.125 (0.095 - 0.165) |  | 0.021 (0.016 - 0.029) |  |
|  | 14.2 – 15.0 g/dl | c | 614 | 0.125 (0.092 - 0.159) |  | 0.020 (0.015 - 0.027) |  |
|  | > 15.0 g/dl | d | 588 | 0.123 (0.088 - 0.159) |  | 0.020 (0.015 - 0.026) |  |
| RBC (Quartiles) | ≤ 4.45 Mio/µl | a | 595 | 0.121 (0.092 - 0.165) | 0.575 | 0.020 (0.015 - 0.028) | 0.908 |
|  | 4.46 – 4.70 Mio/µl | b | 629 | 0.125 (0.088 - 0.159) |  | 0.021 (0.015 - 0.028) |  |
|  | 4.71 – 5.0 Mio/µl | c | 600 | 0.125 (0.092 - 0.165) |  | 0.020 (0.015 - 0.028) |  |
|  | > 5.0 Mio/µl | d | 591 | 0.125 (0.098 - 0.165) |  | 0.020 (0.015 - 0.027) |  |
| White Blood cells (Quartiles) | < 5.00 Tsd/µl | a | 620 | 0.129 (0.092 - 0.171)^d^ | 0.007 | 0.021 (0.016 - 0.029) | 0.062 |
|  | 5.00 – 5.89 Tsd/µl | b | 588 | 0.125 (0.095 - 0.165) |  | 0.020 (0.015 - 0.027) |  |
|  | 5.90 – 6.91 Tsd/µl | c | 613 | 0.125 (0.095 - 0.165) |  | 0.021 (0.015 - 0.028) |  |
|  | ≥ 6.91 Tsd/µl | d | 607 | 0.117 (0.085 - 0.154)^a^ |  | 0.020 (0.015 - 0.027) |  |
| Monocytes (Quartiles) | < 364 n/µl | a | 599 | 0.134 (0.098 - 0.171)^c.d^ | < 0.001 | 0.022 (0.016 - 0.030)^c.d^ | < 0.001 |
|  | 364 – 452 n/µl | b | 600 | 0.129 (0.098 - 0.165)^d^ |  | 0.021 (0.016 - 0.029)^d^ |  |
|  | 452 – 566 n/µl | c | 600 | 0.121 (0.088 - 0.159)^a^ |  | 0.019 (0.015 - 0.026)^b^ |  |
|  | > 566 n/µl | d | 599 | 0.113 (0.085 - 0.149)^a.b^ |  | 0.019 (0.014 - 0.026)^a.b^ |  |
| Lymphocytes (Quartiles) | < 1540 n/µl | a | 598 | 0.117 (0.088 - 0.154)^d^ | 0.016 | 0.020 (0.015 - 0.028) | 0.521 |
|  | 1540 – 1890 n/µl | b | 600 | 0.129 (0.092 - 0.165) |  | 0.021 (0.016 - 0.029) |  |
|  | 1891 – 2281 n/µl | c | 599 | 0.121 (0.095 - 0.159) |  | 0.021 (0.015 - 0.027) |  |
|  | > 2281 n/µl | d | 599 | 0.129 (0.095 - 0.171)^a^ |  | 0.020 (0.015 - 0.028) |  |
| Lymphocytes/Monocytes (Quartiles) | < 3.35 | a | 598 | 0.109 (0.082 - 0.144)^b.c.d^ | < 0.001 | 0.019 (0.014 - 0.025)^c.d^ | < 0.001 |
|  | 3.35-4.20 | b | 598 | 0.125 (0.088 - 0.159)^a.d^ |  | 0.020 (0.015 - 0.027)^d^ |  |
|  | 4.20-5.24 | c | 599 | 0.129 (0.098 - 0.165)^a^ |  | 0.021 (0.016 - 0.028)^a^ |  |
|  | > 5.24 | d | 598 | 0.134 (0.102 - 0.177)^a.b^ |  | 0.021 (0.016 - 0.030)^a.b^ |  |
| CD3+CD45+ cells % (Quartiles) | < 70 % | a | 442 | 0.121 (0.092 - 0.159)^b.c.d^ | < 0.001 | 0.019 (0.015 - 0.026)^d^ | 0.002 |
|  | 70-75 % | b | 378 | 0.134 (0.105 - 0.171)^a^ |  | 0.021 (0.015 - 0.026) |  |
|  | 75-80 % | c | 417 | 0.134 (0.102 - 0.171)^a^ |  | 0.021 (0.015 - 0.027) |  |
|  | > 80 % | d | 448 | 0.139 (0.105 - 0.183)^a^ |  | 0.022 (0.016 - 0.029)^a^ |  |
| Neutrophils (Quartiles) | < 2.56 Tsd/µl | a | 599 | 0.134 (0.095 - 0.171)^b.c.d^ | < 0.001 | 0.021 (0.016 - 0.029)^d^ | 0.042 |
|  | 2.56 – 3.20 Tsd/µl | b | 590 | 0.125 (0.095 - 0.165)^a^ |  | 0.020 (0.015 - 0.027) |  |
|  | 3.20 – 3.99 Tsd/µl | c | 612 | 0.125 (0.092 - 0.165)^a^ |  | 0.021 (0.015 - 0.028) |  |
|  | ≥ 3.99 Tsd/µl | d | 601 | 0.117 (0.085 - 0.149)^a^ |  | 0.020 (0.015 - 0.027)^a^ |  |
| Eosinophils (Quartiles) | ≤ 100 n/µl | a | 602 | 0.125 (0.095 - 0.171) | 0.051 | 0.021 (0.015 - 0.029) | 0.591 |
|  | 100 – 151 n/µl | b | 592 | 0.125 (0.095 - 0.165) |  | 0.020 (0.015 - 0.026) |  |
|  | 151 – 231 n/µl | c | 585 | 0.125 (0.092 - 0.159) |  | 0.020 (0.015 - 0.028) |  |
|  | ≥ 231 n/µl | d | 617 | 0.121 (0.088 - 0.154) |  | 0.020 (0.015 - 0.029) |  |
| Basophils (Quartiles) | ≤ 20.4 n/µl | a | 583 | 0.125 (0.095 - 0.165) | 0.025 | 0.021 (0.015 - 0.028) | 0.218 |
|  | 20.5 – 32.4 n/µl | b | 588 | 0.125 (0.096 - 0.154) |  | 0.020 (0.015 - 0.027) |  |
|  | 32.4 – 48.8 n/µl | c | 600 | 0.129 (0.095 - 0.165)^d^ |  | 0.021 (0.015 - 0.028) |  |
|  | > 48.8 n/µl | d | 625 | 0.117 (0.085 - 0.159)^c^ |  | 0.021 (0.016 - 0.028) |  |
| Platelets (Quartiles) | ≤ 197 Tsd/µl | a | 547 | 0.125 (0.092 - 0.171) | 0.426 | 0.020 (0.015 - 0.028) | 0.004 |
|  | 197 – 229 Tsd/µl | b | 615 | 0.125 (0.092 - 0.159) |  | 0.019 (0.015 - 0.026)^d^ |  |
|  | 230 – 268 Tsd/µl | c | 616 | 0.125 (0.098 - 0.159) |  | 0.021 (0.015 - 0.028) |  |
|  | ≥ 269 Tsd/µl | d | 652 | 0.125 (0.088 - 0.159) |  | 0.021 (0.016 - 0.029)^b^ |  |
| MPV (Quartiles) | ≤ 8.7 fl | a | 443 | 0.129 (0.095 - 0.171)^d^ | 0.003 | 0.021 (0.015 - 0.031)^d^ | 0.006 |
|  | 8.7 – 9.6 fl | b | 395 | 0.129 (0.095 - 0.165)^d^ |  | 0.021 (0.015 - 0.027) |  |
|  | 9.7 – 10.7 fl | c | 415 | 0.121 (0.085 - 0.159) |  | 0.020 (0.015 - 0.028) |  |
|  | ≥ 10.8 fl | d | 385 | 0.117 (0.085 - 0.154)^a.b^ |  | 0.019 (0.014 - 0.026)^a^ |  |

^1^ Non-parametric comparison by the Kruskal-Wallis test (KW test) of *DNMT1* and *DNMT3B* mRNAs (data for two group comparison are analysed with the Mann-Whitney U test); data are reported as median and interquartile range (IQ). Pairwise comparisons (adjusted for multiple comparisons) with p < 0.05 are marked by the associated superscripts. Outliers are excluded in the analysis. Lymphocytes/Monocytes = ratio between lymphocytes and monocytes from haemochrome analysis. All variables related to food are referred to the consumption as indicated. Definition of abbreviations is provided in the supplementary list.

**Supplementary Table 7 A. Contribution of selected variables on age-related changes of *DNMT1* expression in the RASIG population^1^ (the table continues in Table 7 B).**

| **Variable** | **Model**  **1** | **Model**  **2** | **Model**  **3** | **Model**  **4** | **Model**  **5** | **Model**  **6** | **Model**  **7** | **Model**  **8** | **Model**  **9** |
| --- | --- | --- | --- | --- | --- | --- | --- | --- | --- |
| **N of subjects** | **1772** | **1772** | **1772** | **1736** | **1735** | **1736** | **1736** | **1736** | **1736** |
|  | **Wald**  **Sig.** | **Wald**  **Sig.** | **Wald**  **Sig.** | **Wald**  **Sig.** | **Wald**  **Sig.** | **Wald**  **Sig.** | **Wald**  **Sig.** | **Wald**  **Sig.** | **Wald**  **Sig.** |
| **Age group** | 10.513  0.015 | 8.474  0.037 | 8.407  0.038 | 10.059  0.018 | 9.924  0.019 | 8.780  0.017 | 8.673  0.018 | 9.697  0.021 | 9.657  0.022 |
| **Country** |  | 69.122  < 0.001 | 69.751  < 0.001 | 57.670  <0.001 | 55.210  < 0.001 | 57.291  < 0.001 | 52.941  < 0.001 | 52.220  < 0.001 | 54.142  < 0.001 |
| **Age group *Country** |  | 49.540  < 0.001 | 49.718  < 0.001 | 56.284  < 0.001 | 54.833  < 0.001 | 55.629  < 0.001 | 55.694  < 0.001 | 56.022  < 0.001 | 54.960  < 0.001 |
| **Gender** |  |  | 4.476  0.042 | 0.004  0.951 | 0.001  0.989 | 0.010  0.920 | 0.011  0.916 | 0.003  0.959 | 0.002  0.967 |
| **Lymphocyte to monocyte ratio** |  |  |  | 58.525  < 0.001 | 58.006  < 0.001 | 58.908  < 0.001 | 58.344  < 0.001 | 58.461  < 0.001 | 56.455  < 0.001 |
| **BMI classes** |  |  |  |  | 0.689  0.709 |  |  |  |  |
| **Dairy Products** |  |  |  |  |  | 5.890  0.053 |  |  |  |
| **Fries fried** |  |  |  |  |  |  | 2.070  0.355 |  |  |
| **Brown bread consumption** |  |  |  |  |  |  |  | 1.532  0.465 |  |
| **White bread consumption** |  |  |  |  |  |  |  |  | 4.232  0.121 |

**Supplementary Table 7 B. Contribution of selected variables on age-related changes of *DNMT1* expression in the RASIG population^1^.**

| **Variable** | **Model**  **10** | **Model**  **11** | **Model**  **12** | **Model**  **13** | **Model**  **16** | **Model**  **17** | **Model**  **18** | **Model**  **19** | **Model**  **20** | **Model**  **21** |
| --- | --- | --- | --- | --- | --- | --- | --- | --- | --- | --- |
| **N of subjects** | **1736** | **1624** | **1735** | **1735** | **1735** | **1736** | **1736** | **1736** | **1736** | **1621** |
|  | **Wald**  **Sig.** | **Wald**  **Sig.** | **Wald**  **Sig.** | **Wald**  **Sig.** | **Wald**  **Sig.** | **Wald**  **Sig.** | **Wald**  **Sig.** | **Wald**  **Sig.** | **Wald**  **Sig.** | **Wald**  **Sig.** |
| **Age group** | 10.077  0.018 | 9.069  0.028 | 9.960  0.019 | 9.617  0.022 | 9.469  0.024 | 9.840  0.024 | 10.311  0.016 | 10.508  0.015 | 10.143  0.017 | 7.996  0.046 |
| **Country** | 56.323  < 0.001 | 49.013  < 0.001 | 53.443  < 0.001 | 50.299  < 0.001 | 54.244  < 0.001 | 59.206  < 0.001 | 56.335  < 0.001 | 62.430  < 0.001 | 57.731  < 0.001 | 30.075  < 0.001 |
| **Age group *Country** | 56.187  < 0.001 | 56.398  < 0.001 | 56.663  < 0.001 | 57.123  < 0.001 | 57.045  < 0.001 | 56.145  < 0.001 | 56.956  < 0.001 | 58.044  < 0.001 | 56.019  < 0.001 | 52.059  < 0.001 |
| **Gender** | 0.017  0.896 | 0.057  0.812 | 0.365  0.546 | 0.012  0.913 | 0.355  0.551 | 0.031  0.859 | 0.019  0.892 | 0.015  0.903 | 0.022  0.883 | 0.034  0.853 |
| **Lymphocyte to monocyte ratio** | 58.359  < 0.001 | 51.555  < 0.001 | 58.480  < 0.001 | 59.712  < 0.001 | 58.837  < 0.001 | 41.259  < 0.001 | 53.994  < 0.001 | 45.123  < 0.001 | 58.208  < 0.001 | 16.516  0.001 |
| **BMI classes** |  |  |  |  |  |  |  |  |  | 2.461  0.292 |
| **Dairy Products** |  |  |  |  |  |  |  |  |  | 6.099  0.047 |
| **French fries** |  |  |  |  |  |  |  |  |  | 2.346  0.309 |
| **Brown bread consumption** |  |  |  |  |  |  |  |  |  | 1.146  0.564 |
| **White bread consumption** |  |  |  |  |  |  |  |  |  | 3.720  0.156 |
| **Alcohol consumption** | 0.204  0.903 |  |  |  |  |  |  |  |  | 0.686  0.710 |
| **Serum Glucose** |  | 1.227  0.746 |  |  |  |  |  |  |  | 1.195  0.754 |
| **Homocysteine** |  |  | 5.638  0.131 |  |  |  |  |  |  | 6.825  0.078 |
| **MCV** |  |  |  | 6.365  0.095 |  |  |  |  |  | 4.589  0.204 |
| **HCT** |  |  |  |  | 1.426  0.699 |  |  |  |  | 0.165  0.983 |
| **Monocytes** |  |  |  |  |  | 2.681  0.443 |  |  |  | 1.036  0.793 |
| **Lymphocytes** |  |  |  |  |  |  | 3.909  0.271 |  |  | 3.340  0.342 |
| **Neutrophils** |  |  |  |  |  |  |  | 18.353  < 0.001 |  | 13.542  0.004 |
| **Eosinophils** |  |  |  |  |  |  |  |  | 2.456  0.483 | 1.680  0.641 |

^1^ Variables were selected on the basis of significant effect on *DNMT1* based on the Kruskal-Wallis test; Analysis was performed by generalized linear model with gamma distribution and log-link function; all 2-way interactions with age group were tested and those not significant were removed from the models. All variables were categorized as previously indicated. Definition of abbreviations is provided in the supplementary list.

**Supplementary Table 8 A. Contribution of selected variables on age-related changes of *DNMT1* expression in the RASIG population^1^ (the table continues in Table 8 B).**

| **Variable** | **Model**  **1** | **Model**  **2** | **Model**  **3** | **Model**  **4** | **Model**  **5** | **Model**  **6** | **Model**  **7** | **Model**  **8** | **Model**  **9** |
| --- | --- | --- | --- | --- | --- | --- | --- | --- | --- |
| **N of subjects** | **1772** | **1772** | **1772** | **1736** | **1735** | **1736** | **1736** | **1736** | **1736** |
|  | **Wald**  **Sig.** | **Wald**  **Sig.** | **Wald**  **Sig.** | **Wald**  **Sig.** | **Wald**  **Sig.** | **Wald**  **Sig.** | **Wald**  **Sig.** | **Wald**  **Sig.** | **Wald**  **Sig.** |
| **Age group** | 13.503  0.004 | 12.000  0.007 | 11.939  0.008 | 13.782  0.003 | 13.710  0.003 | 13.978  0.003 | 13.617  0.004 | 13.488  0.004 | 13.381  0.004 |
| **Country** |  | 61.840  < 0.001 | 62.055  < 0.001 | 52.060  <0.001 | 49.433  < 0.001 | 51.609  < 0.001 | 47.627  < 0.001 | 47.655  < 0.001 | 46.302  < 0.001 |
| **Age group *Country** |  | 46.295  < 0.001 | 46.710  < 0.001 | 50.975  < 0.001 | 49.863  < 0.001 | 50.276  < 0.001 | 50.435  < 0.001 | 50.095  < 0.001 | 49.520  < 0.001 |
| **Gender** |  |  | 3.314  0.069 | 0.010  0.921 | 0.016  0.899 | 0.013  0.909 | 0.013  0.910 | 0.003  0.959 | 0.000  0.991 |
| **Lymphocyte to monocyte ratio** |  |  |  | 50.215  < 0.001 | 49.635  < 0.001 | 50.489  < 0.001 | 50.589  < 0.001 | 50.926  < 0.001 | 49.345  < 0.001 |
| **BMI classes** |  |  |  |  | 1.433  0.488 |  |  |  |  |
| **Dairy Products** |  |  |  |  |  | 5.866  0.053 |  |  |  |
| **Fries fried** |  |  |  |  |  |  | 3.414  0.181 |  |  |
| **Brown bread consumption** |  |  |  |  |  |  |  | 3.028  0.220 |  |
| **White bread consumption** |  |  |  |  |  |  |  |  | 2.779  0.249 |

**Supplementary Table 8 B. Contribution of selected variables on age-related changes of *DNMT1* expression in the RASIG population^1^.**

| **Variable** | **Model**  **10** | **Model**  **11** | **Model**  **12** | **Model**  **13** | **Model**  **16** | **Model**  **17** | **Model**  **18** | **Model**  **19** | **Model**  **20** | **Model**  **21** |
| --- | --- | --- | --- | --- | --- | --- | --- | --- | --- | --- |
| **N of subjects** | **1736** | **1624** | **1735** | **1735** | **1735** | **1736** | **1736** | **1736** | **1736** | **1621** |
|  | **Wald**  **Sig.** | **Wald**  **Sig.** | **Wald**  **Sig.** | **Wald**  **Sig.** | **Wald**  **Sig.** | **Wald**  **Sig.** | **Wald**  **Sig.** | **Wald**  **Sig.** | **Wald**  **Sig.** | **Wald**  **Sig.** |
| **Age group** | 13.788  0.003 | 13.454  0.004 | 13.411  0.004 | 13.792  0.003 | 12.826  0.005 | 13.525  0.004 | 13.847  0.003 | 13.710  0.003 | 13.752  0.003 | 11.644  0.009 |
| **Country** | 50.889  < 0.001 | 44.071  < 0.001 | 45.586  < 0.001 | 45.727  < 0.001 | 48.302  < 0.001 | 52.889  < 0.001 | 50.037  < 0.001 | 57.096  < 0.001 | 51.801  < 0.001 | 23.225  0.001 |
| **Age group *Country** | 50.574  < 0.001 | 52.372  < 0.001 | 51.690  < 0.001 | 51.416  < 0.001 | 51.268  < 0.001 | 50.668  < 0.001 | 51.296  < 0.001 | 51.779  < 0.001 | 51.048  < 0.001 | 47.059  < 0.001 |
| **Gender** | 0.059  0.807 | 0.015  0.902 | 0.299  0.585 | 0.034  0.853 | 0.538  0.463 | 0.011  0.917 | 0.010  0.921 | 0.024  0.876 | 0.007  0.883 | 0.137  0.711 |
| **Lymphocyte to monocyte ratio** | 49.950  < 0.001 | 42.609  < 0.001 | 50.167  < 0.001 | 51.822  < 0.001 | 50.691  < 0.001 | 37.603  < 0.001 | 42.465  < 0.001 | 38.214  < 0.001 | 49.970  < 0.001 | 12.681  0.005 |
| **BMI classes** |  |  |  |  |  |  |  |  |  | 3.123  0.210 |
| **Dairy Products** |  |  |  |  |  |  |  |  |  | 6.013  0.049 |
| **French fries** |  |  |  |  |  |  |  |  |  | 3.535  0.171 |
| **Brown bread consumption** |  |  |  |  |  |  |  |  |  | 2.240  0.326 |
| **White bread consumption** |  |  |  |  |  |  |  |  |  | 3.974  0.137 |
| **Alcohol consumption** | 1.376  0.503 |  |  |  |  |  |  |  |  | 2.558  0.278 |
| **Serum Glucose** |  | 1.834  0.608 |  |  |  |  |  |  |  | 1.326  0.723 |
| **Homocysteine** |  |  | 5.234  0.155 |  |  |  |  |  |  | 6.708  0.082 |
| **MCV** |  |  |  | 8.255  0.004 |  |  |  |  |  | 6.702  0.082 |
| **HCT** |  |  |  |  | 1.561  0.668 |  |  |  |  | 0.359  0.949 |
| **Monocytes** |  |  |  |  |  | 2.339  0.505 |  |  |  | 1.370  0.713 |
| **Lymphocytes** |  |  |  |  |  |  | 1.502  0.682 |  |  | 1.346  0.718 |
| **Neutrophils** |  |  |  |  |  |  |  | 15.841  < 0.001 |  | 13.454  0.004 |
| **Eosinophils** |  |  |  |  |  |  |  |  | 1.000  0.801 | 1.395  0.707 |

^1^ Variables were selected on the basis of significant effect on *DNMT1* based on the Kruskal-Wallis test; Analysis was performed by generalized linear model with log-transformed *DNMT1* and identity-link function; All variables were categorized as previously indicated. Definition of abbreviations is provided in the supplementary list.

**Supplementary Table 9. Stratified (gender and country) comparison of *DNMT1* expression among GO, SGO and RASIG**

|  |  |  |  |  | **Females** |  |  |  |  |  |  | **Males** |  |  |  |  |  | **Total** |  |  |
| --- | --- | --- | --- | --- | --- | --- | --- | --- | --- | --- | --- | --- | --- | --- | --- | --- | --- | --- | --- | --- |
|  |  |  | **GO** |  | **RASIG** |  | **SGO** |  |  | **GO** |  | **RASIG** |  | **SGO** |  | **GO** |  | **RASIG** |  | **SGO** |
|  |  | **N** | **Median**  **(IQ)** | **N** | **Median**  **(IQ)** | **N** | **Median**  **(IQ)** |  | **N** | **Median**  **(IQ)** | **N** | **Median**  **(IQ)** | **N** | **Median**  **(IQ)** | **N** | **Median**  **(IQ)** | **N** | **Median**  **(IQ)** | **N** | **Median**  **(IQ)** |
| **Finland** | **35-44** | 0 | - | 7 | 0.129  (0.125 -0.183) | 0 | - | | 0 | - | 2 | 0.142  (0.125 -0.159) | 0 | - | 0 | - | 9 | 0.129  (0.125 -0.159) | 0 | - |
|  | **45-54** | 4 | 0.079  (0.067 -0.105) | 4 | 0.125  (0.111 -0.144) | 4 | 0.097  (0.056 -0.136) | | 2 | 0.062  (0.056 -0.067) | 3 | 0.159  (0.117 -0.171) | 1 | 0.330  (0.330 -0.330) | 6 | 0.069 *  (0.063 -0.085) | 7 | 0.134  (0.117 -0.159) | 5 | 0.102  (0.092 -0.171) |
|  | **55-64** | 36 | 0.100  (0.061 -0.136) | 25 | 0.109  (0.092 -0.125) | 15 | 0.082  (0.065 -0.177) | | 20 | 0.111  (0.096 -0.151) | 6 | 0.134  (0.082 -0.144) | 7 | 0.098  (0.051 -0.149) | 56 | 0.103  (0.073 -0.141) | 31 | 0.109  (0.082 -0.129) | 22 | 0.093  (0.065 -0.149) |
|  | **65-75** | 33 | 0.113  (0.074 -0.159) | 21 | 0.117  (0.085 -0.177) | 10 | 0.072  (0.046 -0.129) | | 26 | 0.103  (0.069 -0.139) | 12 | 0.134  (0.093 -0.160) | 11 | 0.080  (0.074 -0.102) | 59 | 0.105  (0.069 -0.154) | 33 | 0.125  (0.092 -0.171) | 21 | 0.077 *  (0.069 -0.102) |
| **Italy** | **35-44** | 0 | - | 47 | 0.149  (0.105 -0.177) | 1 | 0.121  (0.121 -0.121) | | 1 | 0.177  (0.177 -0.177) | 49 | 0.144  (0.121 -0.165) | 0 | - | 1 | 0.177  (0.177 -0.177) | 96 | 0.144  (0.115 -0.174) | 1 | 0.121  (0.121 -0.121) |
|  | **45-54** | 2 | 0.149  (0.102 -0.196) | 50 | 0.129  (0.102 -0.154) | 3 | 0.113  (0.109 -0.113) | | 4 | 0.151  (0.107 -0.291) | 45 | 0.129  (0.113 -0.177) | 1 | 0.144  (0.144 -0.144) | 6 | 0.151  (0.102 -0.196) | 95 | 0.129  (0.102 -0.171) | 4 | 0.113  (0.111 -0.128) |
|  | **55-64** | 13 | 0.139  (0.125 -0.203) | 44 | 0.129  (0.092 -0.165) | 15 | 0.129  (0.105 -0.189) | | 18 | 0.123  (0.098 -0.171) | 42 | 0.151  (0.109 -0.189) | 12 | 0.144  (0.111 -0.180) | 31 | 0.139  (0.102 -0.189) | 86 | 0.139  (0.095 -0.183) | 27 | 0.134  (0.109 -0.183) |
|  | **65-75** | 23 | 0.125 *  (0.105 -0.165) | 41 | 0.183  (0.149 -0.210) | 4 | 0.141  (0.113 -0.160) | | 17 | 0.149  (0.109 -0.171) | 44 | 0.162  (0.123 -0.183) | 9 | 0.144  (0.113 -0.189) | 40 | 0.134 *  (0.105 -0.168) | 85 | 0.171  (0.134 -0.203) | 13 | 0.144  (0.113 -0.171) |
| **Austria** | **35-44** | 0 | - | 33 | 0.134  (0.109 -0.196) | 0 | - | | 0 | - | 36 | 0.117  (0.093 -0.165) | 0 | - | 0 | - | 69 | 0.125  (0.102 -0.183) | 0 | - |
|  | **45-54** | 0 | - | 32 | 0.125  (0.083 -0.171) | 0 | - | | 0 | - | 37 | 0.125  (0.082 -0.177) | 0 | - | 0 | - | 69 | 0.125  (0.082 -0.177) | 0 | - |
|  | **55-64** | 0 | - | 35 | 0.092  (0.065 -0.117) | 0 | - | | 0 | - | 34 | 0.102  (0.077 -0.121) | 0 | - | 0 | - | 69 | 0.098  (0.067 -0.117) | 0 | - |
|  | **65-75** | 0 | - | 32 | 0.111  (0.073 -0.149) | 0 | - | | 0 | - | 28 | 0.119  (0.090 -0.151) | 0 | - | 0 | - | 60 | 0.113  (0.077 -0.149) | 0 | - |
| **Greece** | **35-44** | 0 | - | 34 | 0.151  (0.102 -0.196) | 0 | - | | 0 | - | 37 | 0.125  (0.082 -0.154) | 0 | - | 0 | - | 71 | 0.134  (0.095 -0.177) | 0 | - |
|  | **45-54** | 1 | 0.113  (0.113 -0.113) | 39 | 0.144  (0.113 -0.171) | 1 | 0.105  (0.105 -0.105) | | 0 | - | 37 | 0.105  (0.067 -0.154) | 0 | - | 1 | 0.113  (0.113 -0.113) | 76 | 0.127  (0.088 -0.165) | 1 | 0.105  (0.105 -0.105) |
|  | **55-64** | 8 | 0.095 *  (0.065 -0.119) | 34 | 0.141  (0.109 -0.203) | 0 | - | | 3 | 0.085  (0.067 -0.139) | 40 | 0.087  (0.071 -0.123) | 1 | 0.035  (0.035 -0.035) | 11 | 0.095  (0.067 -0.125) | 74 | 0.117  (0.085 -0.159) | 1 | 0.035  (0.035 -0.035) |
|  | **65-75** | 3 | 0.088  (0.088 -0.125) | 40 | 0.162  (0.125 -0.210) | 1 | 0.047  (0.047 -0.047) | | 2 | 0.045  (0.044 -0.046) | 35 | 0.125  (0.092 -0.159) | 1 | 0.139  (0.139 -0.139) | 5 | 0.088 *  (0.046 -0.088) | 75 | 0.149  (0.105 -0.183) | 2 | 0.093  (0.047 -0.139) |
| **Poland** | **35-44** | 0 | - | 28 | 0.139  (0.121 -0.157) | 0 | - | | 0 | - | 18 | 0.162  (0.105 -0.210) | 0 | - | 0 | - | 46 | 0.149  (0.117 -0.177) | 0 | 0.000  (0.000 -0.000) |
|  | **45-54** | 3 | 0.092  (0.067 -0.259) | 22 | 0.141  (0.109 -0.189) | 1 | 0.065  (0.065 -0.065) | | 0 | - | 25 | 0.139  (0.113 -0.177) | 1 | 0.171  (0.171 -0.171) | 3 | 0.092  (0.067 -0.259) | 47 | 0.139  (0.109 -0.189) | 2 | 0.118  (0.065 -0.171) |
|  | **55-64** | 15 | 0.109  (0.060 -0.159) | 29 | 0.139  (0.085 -0.165) | 6 | 0.073  (0.069 -0.117) | | 7 | 0.074 *  (0.054 -0.082) | 32 | 0.129  (0.105 -0.174) | 10 | 0.085 *  (0.060 -0.109) | 22 | 0.078 *  (0.060 -0.139) | 61 | 0.139  (0.105 -0.171) | 16 | 0.081 *  (0.065 -0.113) |
|  | **65-75** | 12 | 0.102  (0.090 -0.132) | 31 | 0.125  (0.092 -0.154) | 1 | 0.189  (0.189 -0.189) | | 6 | 0.126  (0.077 -0.154) | 17 | 0.144  (0.117 -0.196) | 6 | 0.121  (0.088 -0.177) | 18 | 0.109  (0.088 -0.139) | 48 | 0.127  (0.098 -0.165) | 7 | 0.125  (0.088 -0.183) |
| **The Netherlands** | **35-44** | 0 | - | 0 | - | 0 | - | | 0 | - | 0 | - | 0 | - | 0 | - | 0 | - | 0 | - |
|  | **45-54** | 1 | 0.092  (0.092 -0.092) | 0 | - | 2 | 0.180  (0.177 -0.183) | | 0 | - | 0 | - | 0 | - | 1 | 0.092  (0.092 -0.092) | 0 | - | 2 | 0.180  (0.177 -0.183) |
|  | **55-64** | 24 | 0.149  (0.103 -0.193) | 0 | - | 21 | 0.149  (0.117 -0.171) | | 19 | 0.154  (0.098 -0.177) | 0 | - | 15 | 0.139  (0.098 -0.165) | 43 | 0.149  (0.098 -0.177) | 0 | - | 36 | 0.146  (0.102 -0.168) |
|  | **65-75** | 29 | 0.139  (0.098 -0.171) | 0 | - | 15 | 0.139  (0.102 -0.154) | | 31 | 0.125  (0.109 -0.154) | 0 | - | 32 | 0.125  (0.111 -0.146) | 60 | 0.125  (0.103 -0.162) | 0 | - | 47 | 0.129  (0.109 -0.149) |
| **Belgium** | **35-44** | 0 | - | 18 | 0.098  (0.069 -0.113) | 0 | - | | 0 | - | 12 | 0.125  (0.085 -0.162) | 0 | - | 0 | - | 30 | 0.098  (0.074 -0.129) | 0 | 0.000  (0.000 -0.000) |
|  | **45-54** | 0 | - | 37 | 0.117  (0.085 -0.144) | 3 | 0.129  (0.095 -0.134) | | 0 | - | 28 | 0.115  (0.071 -0.139) | 1 | 0.095  (0.095 -0.095) | 0 | - | 65 | 0.117  (0.082 -0.139) | 4 | 0.112  (0.095 -0.132) |
|  | **55-64** | 20 | 0.102  (0.084 -0.137) | 38 | 0.134  (0.085 -0.154) | 14 | 0.102  (0.069 -0.129) | | 22 | 0.115  (0.085 -0.134) | 34 | 0.107  (0.072 -0.149) | 3 | 0.088  (0.065 -0.113) | 42 | 0.105  (0.085 -0.134) | 72 | 0.121  (0.083 -0.151) | 17 | 0.098  (0.069 -0.113) |
|  | **65-75** | 22 | 0.105  (0.072 -0.129) | 37 | 0.098  (0.067 -0.129) | 2 | 0.097  (0.065 -0.129) | | 10 | 0.083  (0.063 -0.109) | 37 | 0.102  (0.077 -0.125) | 12 | 0.098  (0.085 -0.121) | 32 | 0.100  (0.065 -0.127) | 74 | 0.102  (0.072 -0.129) | 14 | 0.098  (0.085 -0.125) |
| **Germany** | **35-44** | 0 | - | 38 | 0.123  (0.095 -0.154) | 0 | - | | 0 | - | 22 | 0.139  (0.095 -0.183) | 0 | - | 0 | - | 60 | 0.129  (0.095 -0.159) | 0 | - |
|  | **45-54** | 0 | - | 49 | 0.129  (0.105 -0.149) | 0 | - | | 0 | - | 41 | 0.134  (0.102 -0.165) | 0 | - | 0 | - | 90 | 0.132  (0.105 -0.154) | 0 | - |
|  | **55-64** | 0 | - | 49 | 0.117  (0.098 -0.165) | 0 | - | | 0 | - | 41 | 0.125  (0.092 -0.171) | 0 | - | 0 | - | 90 | 0.119  (0.098 -0.171) | 0 | - |
|  | **65-75** | 0 | - | 46 | 0.136  (0.113 -0.159) | 0 | - | | 0 | - | 40 | 0.113  (0.088 -0.146) | 0 | - | 0 | - | 86 | 0.127  (0.109 -0.154) | 0 | - |
| **Total** | **35-44** | 0 | - | 205 | 0.134  (0.102 -0.171) | 1 | 0.121  (0.121 -0.121) | | 1 | 0.177  (0.177 -0.177) | 176 | 0.134  (0.098 -0.168) | 0 | 0.000  (0.000 -0.000) | 1 | 0.177  (0.177 -0.177) | 381 | 0.134  (0.102 -0.171) | 1 | 0.121  (0.121 -0.121) |
|  | **45-54** | 11 | 0.092  (0.072 -0.125) | 233 | 0.129  (0.102 -0.159) | 14 | 0.111  (0.095 -0.134) | | 6 | 0.107  (0.067 -0.189) | 216 | 0.129  (0.093 -0.171) | 4 | 0.157  (0.119 -0.250) | 17 | 0.102  (0.072 -0.125) | 449 | 0.129  (0.095 -0.159) | 18 | 0.113  (0.095 -0.171) |
|  | **55-64** | 116 | 0.115  (0.080 -0.159) | 254 | 0.121  (0.092 -0.165) | 71 | 0.117  (0.080 -0.165) | | 89 | 0.113  (0.088 -0.154) | 229 | 0.113  (0.085 -0.159) | 48 | 0.111  (0.088 -0.165) | 205 | 0.113  (0.082 -0.154) | 483 | 0.117  (0.088 -0.165) | 119 | 0.113  (0.082 -0.165) |
|  | **65-75** | 122 | 0.117 *  (0.088 -0.154) | 248 | 0.136  (0.102 -0.177) | 33 | 0.129  (0.082 -0.149) | | 92 | 0.115  (0.081 -0.154) | 213 | 0.121  (0.092 -0.165) | 71 | 0.117  (0.092 -0.144) | 214 | 0.117 *  (0.088 -0.154) | 461 | 0.129  (0.095 -0.171) | 104 | 0.117 *  (0.092 -0.144) |

*** P < 0.05 compared to RASIG by the Kruskal-Wallis test (the reported pairwise comparison are adjusted for multiple comparison)**

**Supplementary Table 10. Stratified (gender and country) comparison of *DNMT3B* expression among GO, SGO and RASIG**

|  |  |  |  |  | **Females** |  |  |  |  |  |  | **Males** |  |  |  |  |  | **Total** |  |  |
| --- | --- | --- | --- | --- | --- | --- | --- | --- | --- | --- | --- | --- | --- | --- | --- | --- | --- | --- | --- | --- |
|  |  |  | **GO** |  | **RASIG** |  | **SGO** |  |  | **GO** |  | **RASIG** |  | **SGO** |  | **GO** |  | **RASIG** |  | **SGO** |
|  |  | **N** | **Median**  **((IQ)** | **N** | **Median**  **((IQ)** | **N** | **Median**  **((IQ)** |  | **N** | **Median**  **((IQ)** | **N** | **Median**  **((IQ)** | **N** | **Median**  **((IQ)** | **N** | **Median**  **((IQ)** | **N** | **Median**  **((IQ)** | **N** | **Median**  **(IQ)** |
| **Finland** | **35-44** | 0 | - | 7 | 0.031  (0.029 -0.035) | 0 | - | | 0 | - | 2 | 0.021  (0.019 -0.023) | 0 | - | 0 | - | 9 | 0.031  (0.023 -0.033) | 0 | - |
|  | **45-54** | 4 | 0.044  (0.027 -0.050) | 4 | 0.019  (0.015 -0.021) | 4 | 0.022  (0.016 -0.025) | | 2 | 0.015  (0.014 -0.016) | 3 | 0.027  (0.016 -0.049) | 1 | 0.016  (0.016 -0.016) | 6 | 0.028  (0.015 -0.049) | 7 | 0.019  (0.016 -0.027) | 5 | 0.019  (0.016 -0.025) |
|  | **55-64** | 36 | 0.022  (0.016 -0.036) | 25 | 0.025  (0.019 -0.035) | 15 | 0.029  (0.023 -0.037) | | 20 | 0.024  (0.019 -0.028) | 6 | 0.025  (0.024 -0.025) | 7 | 0.024  (0.018 -0.035) | 56 | 0.023  (0.018 -0.030) | 31 | 0.025  (0.019 -0.031) | 22 | 0.029  (0.021 -0.035) |
|  | **65-75** | 33 | 0.021  (0.017 -0.028) | 21 | 0.022  (0.016 -0.028) | 10 | 0.026  (0.021 -0.032) | | 26 | 0.022  (0.014 -0.028) | 12 | 0.023  (0.018 -0.032) | 11 | 0.021  (0.016 -0.026) | 59 | 0.022  (0.017 -0.028) | 33 | 0.022  (0.016 -0.028) | 21 | 0.022  (0.019 -0.030) |
| **Italy** | **35-44** | 0 | - | 47 | 0.021  (0.016 -0.025) | 1 | 0.027  (0.027 -0.027) | | 1 | 0.015  (0.015 -0.015) | 49 | 0.021  (0.015 -0.027) | 0 | - | 1 | 0.015  (0.015 -0.015) | 96 | 0.021  (0.015 -0.027) | 1 | 0.027  (0.027 -0.027) |
|  | **45-54** | 2 | 0.016  (0.016 -0.016) | 50 | 0.021  (0.016 -0.027) | 3 | 0.022  (0.013 -0.028) | | 4 | 0.015  (0.012 -0.021) | 45 | 0.021  (0.016 -0.027) | 1 | 0.024  (0.024 -0.024) | 6 | 0.016  (0.014 -0.016) | 95 | 0.021  (0.016 -0.027) | 4 | 0.023  (0.018 -0.026) |
|  | **55-64** | 13 | 0.027  (0.019 -0.033) | 44 | 0.020  (0.015 -0.024) | 15 | 0.026  (0.018 -0.035) | | 18 | 0.029*  (0.019 -0.037) | 42 | 0.018  (0.015 -0.025) | 12 | 0.026*  (0.020 -0.029) | 31 | 0.028 *  (0.019 -0.037) | 86 | 0.019  (0.015 -0.025) | 27 | 0.026 *  (0.018 -0.031) |
|  | **65-75** | 23 | 0.024  (0.019 -0.029) | 41 | 0.021  (0.016 -0.027) | 4 | 0.024  (0.015 -0.032) | | 17 | 0.021  (0.019 -0.030) | 44 | 0.018  (0.015 -0.025) | 9 | 0.026  (0.017 -0.027) | 40 | 0.024  (0.019 -0.029) | 85 | 0.020  (0.015 -0.025) | 13 | 0.026  (0.017 -0.029) |
| **Austria** | **35-44** | 0 | - | 33 | 0.030  (0.021 -0.041) | 0 | - | | 0 | - | 36 | 0.021  (0.016 -0.031) | 0 | - | 0 | - | 69 | 0.023  (0.019 -0.035) | 0 | - |
|  | **45-54** | 0 | - | 32 | 0.027  (0.014 -0.036) | 0 | - | | 0 | - | 37 | 0.022  (0.013 -0.031) | 0 | - | 0 | - | 69 | 0.025  (0.013 -0.033) | 0 | - |
|  | **55-64** | 0 | - | 35 | 0.021  (0.011 -0.033) | 0 | - | | 0 | - | 34 | 0.025  (0.014 -0.037) | 0 | - | 0 | - | 69 | 0.021  (0.012 -0.036) | 0 | - |
|  | **65-75** | 0 | - | 32 | 0.022  (0.017 -0.030) | 0 | - | | 0 | - | 28 | 0.023  (0.017 -0.033) | 0 | - | 0 | - | 60 | 0.022  (0.017 -0.032) | 0 | - |
| **Greece** | **35-44** | 0 | - | 34 | 0.025  (0.017 -0.043) | 0 | - | | 0 | 0.000  (0.000 -0.000) | 37 | 0.023  (0.017 -0.028) | 0 | - | 0 | - | 71 | 0.024  (0.017 -0.032) | 0 | - |
|  | **45-54** | 1 | 0.019  (0.019 -0.019) | 39 | 0.025  (0.018 -0.035) | 1 | 0.012  (0.012 -0.012) | | 0 | 0.000  (0.000 -0.000) | 37 | 0.019  (0.017 -0.035) | 0 | - | 1 | 0.019  (0.019 -0.019) | 76 | 0.021  (0.017 -0.035) | 1 | 0.012  (0.012 -0.012) |
|  | **55-64** | 8 | 0.022  (0.019 -0.032) | 34 | 0.021  (0.017 -0.031) | 0 | - | | 3 | 0.021  (0.012 -0.022) | 40 | 0.017  (0.012 -0.025) | 1 | 0.019  (0.019 -0.019) | 11 | 0.022  (0.015 -0.032) | 74 | 0.019  (0.015 -0.027) | 1 | 0.019  (0.019 -0.019) |
|  | **65-75** | 3 | 0.022  (0.021 -0.037) | 40 | 0.025  (0.017 -0.035) | 1 | 0.009  (0.009 -0.009) | | 2 | 0.016  (0.014 -0.019) | 35 | 0.020  (0.016 -0.027) | 1 | 0.035  (0.035 -0.035) | 5 | 0.021  (0.019 -0.022) | 75 | 0.022  (0.016 -0.031) | 2 | 0.022  (0.009 -0.035) |
| **Poland** | **35-44** | 0 | - | 28 | 0.022  (0.019 -0.028) | 0 | - | | 0 | - | 18 | 0.024  (0.017 -0.031) | 0 | - | 0 | - | 46 | 0.022  (0.019 -0.028) | 0 | - |
|  | **45-54** | 3 | 0.010  (0.010 -0.026) | 22 | 0.026  (0.021 -0.028) | 1 | 0.010  (0.010 -0.010) | | 0 | - | 25 | 0.022  (0.017 -0.029) | 1 | 0.017  (0.017 -0.017) | 3 | 0.010  (0.010 -0.026) | 47 | 0.024  (0.018 -0.028) | 2 | 0.014  (0.010 -0.017) |
|  | **55-64** | 15 | 0.010*  (0.006 -0.021) | 29 | 0.022  (0.017 -0.026) | 6 | 0.013  (0.008 -0.019) | | 7 | 0.010*  (0.004 -0.017) | 32 | 0.019  (0.012 -0.026) | 10 | 0.010*  (0.007 -0.015) | 22 | 0.010 *  (0.006 -0.019) | 61 | 0.021  (0.015 -0.026) | 16 | 0.011 *  (0.008 -0.016) |
|  | **65-75** | 12 | 0.017  (0.011 -0.023) | 31 | 0.020  (0.014 -0.027) | 1 | 0.043  (0.043 -0.043) | | 6 | 0.023  (0.017 -0.035) | 17 | 0.021  (0.014 -0.025) | 6 | 0.014  (0.011 -0.025) | 18 | 0.019  (0.011 -0.025) | 48 | 0.020  (0.014 -0.025) | 7 | 0.014  (0.011 -0.027) |
| **The Netherlands** | **35-44** | 0 | - | 0 | - | 0 | - | | 0 | - | 0 | - | 0 | - | 0 | - | 0 | - | 0 | - |
|  | **45-54** | 1 | 0.041  (0.041 -0.041) | 0 | - | 2 | 0.021  (0.019 -0.023) | | 0 | - | 0 | - | 0 | - | 1 | 0.041  (0.041 -0.041) | 0 | - | 2 | 0.021  (0.019 -0.023) |
|  | **55-64** | 24 | 0.020  (0.017 -0.026) | 0 | - | 21 | 0.018  (0.015 -0.021) | | 19 | 0.019  (0.014 -0.021) | 0 | - | 15 | 0.019  (0.014 -0.029) | 43 | 0.019  (0.016 -0.025) | 0 | - | 36 | 0.018  (0.014 -0.023) |
|  | **65-75** | 29 | 0.016  (0.014 -0.023) | 0 | - | 15 | 0.021  (0.013 -0.027) | | 31 | 0.016  (0.013 -0.020) | 0 | - | 32 | 0.017  (0.014 -0.022) | 60 | 0.016  (0.014 -0.021) | 0 | - | 47 | 0.018  (0.014 -0.023) |
| **Belgium** | **35-44** | 0 | - | 18 | 0.019  (0.017 -0.022) | 0 | - | | 0 | - | 12 | 0.018  (0.013 -0.024) | 0 | - | 0 | - | 30 | 0.019  (0.017 -0.022) | 0 | - |
|  | **45-54** | 0 | - | 37 | 0.018  (0.015 -0.022) | 3 | 0.031  (0.013 -0.038) | | 0 | - | 28 | 0.016  (0.013 -0.023) | 1 | 0.009  (0.009 -0.009) | 0 | - | 65 | 0.017  (0.015 -0.022) | 4 | 0.022  (0.011 -0.035) |
|  | **55-64** | 20 | 0.019  (0.014 -0.032) | 38 | 0.019  (0.017 -0.030) | 14 | 0.018  (0.013 -0.025) | | 22 | 0.020  (0.014 -0.026) | 34 | 0.017  (0.014 -0.026) | 3 | 0.016  (0.010 -0.035) | 42 | 0.020  (0.014 -0.029) | 72 | 0.019  (0.015 -0.028) | 17 | 0.017  (0.013 -0.025) |
|  | **65-75** | 22 | 0.025*  (0.016 -0.032) | 37 | 0.016  (0.013 -0.021) | 2 | 0.017  (0.010 -0.024) | | 10 | 0.015  (0.013 -0.017) | 37 | 0.017  (0.014 -0.024) | 12 | 0.019  (0.013 -0.024) | 32 | 0.020  (0.014 -0.030) | 74 | 0.016  (0.013 -0.022) | 14 | 0.019  (0.013 -0.024) |
| **Germany** | **35-44** | 0 | - | 38 | 0.023  (0.017 -0.027) | 0 | - | | 0 | - | 22 | 0.020  (0.014 -0.028) | 0 | - | 0 | - | 60 | 0.021  (0.015 -0.027) | 0 | - |
|  | **45-54** | 0 | - | 49 | 0.021  (0.017 -0.031) | 0 | - | | 0 | - | 41 | 0.021  (0.018 -0.028) | 0 | - | 0 | - | 90 | 0.021  (0.017 -0.029) | 0 | - |
|  | **55-64** | 0 | - | 49 | 0.021  (0.017 -0.026) | 0 | - | | 0 | - | 41 | 0.019  (0.015 -0.025) | 0 | - | 0 | - | 90 | 0.020  (0.016 -0.026) | 0 | - |
|  | **65-75** | 0 | - | 46 | 0.019  (0.016 -0.025) | 0 | - | | 0 | - | 40 | 0.020  (0.013 -0.025) | 0 | - | 0 | - | 86 | 0.019  (0.015 -0.025) | 0 | - |
| **Total** | **35-44** | 0 | - | 205 | 0.023  (0.018 -0.031) | 1 | 0.027  (0.027 -0.027) | | 1 | 0.015  (0.015 -0.015) | 176 | 0.021  (0.016 -0.028) | 0 | 0.000  (0.000 -0.000) | 1 | 0.015  (0.015 -0.015) | 381 | 0.022  (0.017 -0.029) | 1 | 0.027  (0.027 -0.027) |
|  | **45-54** | 11 | 0.019  (0.015 -0.041) | 233 | 0.021  (0.016 -0.030) | 14 | 0.021  (0.013 -0.026) | | 6 | 0.015  (0.014 -0.016) | 216 | 0.021  (0.015 -0.028) | 4 | 0.017  (0.013 -0.021) | 17 | 0.016  (0.014 -0.026) | 449 | 0.021  (0.016 -0.029) | 18 | 0.019  (0.013 -0.025) |
|  | **55-64** | 116 | 0.021  (0.015 -0.029) | 254 | 0.021  (0.016 -0.028) | 71 | 0.020  (0.015 -0.030) | | 89 | 0.020  (0.016 -0.027) | 229 | 0.019  (0.015 -0.026) | 48 | 0.019  (0.013 -0.028) | 205 | 0.021  (0.015 -0.029) | 483 | 0.020  (0.015 -0.027) | 119 | 0.019  (0.015 -0.029) |
|  | **65-75** | 122 | 0.021  (0.016 -0.028) | 248 | 0.020  (0.015 -0.027) | 33 | 0.021  (0.016 -0.029) | | 92 | 0.019  (0.014 -0.025) | 213 | 0.020  (0.014 -0.025) | 71 | 0.018  (0.014 -0.025) | 214 | 0.020  (0.015 -0.027) | 461 | 0.020  (0.015 -0.026) | 104 | 0.020  (0.014 -0.026) |

*** P < 0.05 compared to RASIG by the Kruskal-Wallis test (the reported pairwise comparison are adjusted for multiple comparison)**

**Supplementary Table 11 A. Contribution of selected variables on group (GO, RASIG and SGO) related changes of *DNMT1* expression in population aged > 54 years^1^ (the table continues in Table 11 B).**

| **Variable** | **Model**  **1** | **Model**  **2** | **Model**  **3** | **Model**  **4** | **Model**  **5** | **Model**  **6** | **Model**  **7** | **Model**  **8** | **Model**  **9** | **Model 10** | **Model**  **11** |
| --- | --- | --- | --- | --- | --- | --- | --- | --- | --- | --- | --- |
| **N of subjects** | **1583** | **1583** | **1583** | **1583** | **1544** | **1541** | **1541** | **1541** | **1541** | **1541** | **1541** |
|  | **Wald**  **Sig.** | **Wald**  **Sig.** | **Wald**  **Sig.** | **Wald**  **Sig.** | **Wald**  **Sig.** | **Wald**  **Sig.** | **Wald**  **Sig.** | **Wald**  **Sig.** | **Wald**  **Sig.** | **Wald**  **Sig.** | **Wald**  **Sig.** |
| **Group (GO, SGO, RASIG)** | 7.714  0.021 | 7.564  0.023 | 32.475  <0.001 | 32.435  <0.001 | 37.604  <0.001 | 38.028  <0.001 | 37.380  <0.001 | 37.089  <0.001 | 36.341  <0.001 | 37.780  <0.001 | 37.293  <0.001 |
| **Age group (55-64 and 65-75)** |  | 3.184  0.074 | 1.601  0.206 | 1.766  0.184 | 3.352  0.067 | 3.520  0.061 | 3.723  0.054 | 2.918  0.088 | 2.862  0.091 | 3.128  0.077 | 2.720  0.099 |
| **Country** |  |  | 113.242  <0.001 | 115.521  <0.001 | 101.561  <0.001 | 101.228  <0.001 | 96.967  <0.001 | 97.663  <0.001 | 99.812  <0.001 | 87.734  <0.001 | 91.631  <0.001 |
| **Group (GO, SGO, RASIG)*Country** |  |  | 29.031  0.001 | 29.797  <0.001 | 28.973  0.001 | 29.637  0.001 | 29.235  <0.001 | 27.846  0.001 | 26.502  0.002 | 29.207  0.001 | 28.296  0.001 |
| **Gender** |  |  |  | 6.112  0.013 | 0.002  0.966 | 0.047  0.828 | 0.007  0.934 | 0.023  0.880 | 0.003  0.953 | 0.001  0.985 | 0.001  0.990 |
| **Lymphocyte to monocyte** |  |  |  |  | 42.350  <0.001 | 42.232  <0.001 | 42.661  <0.001 | 42.516  <0.001 | 42.990  <0.001 | 42.158  <0.001 | 41.662  <0.001 |
| **Vegetables consumption** |  |  |  |  |  | 2.101  0.350 |  |  |  |  |  |
| **Fruit consumption** |  |  |  |  |  |  | 2.863  0.239 |  |  |  |  |
| **Dairy Products** |  |  |  |  |  |  |  | 5.925  0.052 |  |  |  |
| **French fries** |  |  |  |  |  |  |  |  | 4.999  0.082 |  |  |
| **Brown Bread Consumption** |  |  |  |  |  |  |  |  |  | 0.604  0.739 |  |
| **White bread consumption** |  |  |  |  |  |  |  |  |  |  | 2.638  0.267 |

**Supplementary Table 11 B. Contribution of selected variables on group (GO, RASIG and SGO) related changes of *DNMT1* expression in population aged > 54 years^1^.**

| **Variable** | **Model**  **12** | **Model**  **13** | **Model**  **14** | **Model**  **15** | **Model**  **16** | **Model**  **18** | **Model**  **19** | **Model**  **20** | **Model**  **21** | **Model**  **22** | **Model**  **23** | **Model**  **24** |
| --- | --- | --- | --- | --- | --- | --- | --- | --- | --- | --- | --- | --- |
| **N of subjects** | **1541** | **1459** | **1529** | **1537** | **1539** | **1439** | **1540** | **1541** | **1541** | **1541** | **1539** | **1442** |
|  | **Wald**  **Sig.** | **Wald**  **Sig.** | **Wald**  **Sig.** | **Wald**  **Sig.** | **Wald**  **Sig.** | **Wald**  **Sig.** | **Wald**  **Sig.** | **Wald**  **Sig.** | **Wald**  **Sig.** | **Wald**  **Sig.** | **Wald**  **Sig.** | **Wald**  **Sig.** |
| **Group (GO, SGO, RASIG)** | 37.581  <0.001 | 36.159  <0.001 | 40.040  <0.001 | 36.158  <0.001 | 36.698  <0.001 | 36.129  <0.001 | 37.242  < 0.001 | 37.032  < 0.001 | 36.977  < 0.001 | 36.330  < 0.001 | 37.564  < 0.001 | 33.397  < 0.001 |
| **Age group (55-64 and 65-75)** | 3.366  0.067 | 2.314  0.128 | 2.779  0.096 | 3.427  0.064 | 3.500  0.061 | 3.717  0.054 | 3.758  0.053 | 3.516  0.061 | 3.472  0.062 | 3.835  0.050 | 3.503  0.061 | 1.529  0.216 |
| **Country** | 103.100  <0.001 | 84.992  <0.001 | 98.196  <0.001 | 96.558  <0.001 | 93.946  <0.001 | 87.864  < 0.001 | 103.050  < 0.001 | 99.503  < 0.001 | 100.620  < 0.001 | 106.103  < 0.001 | 97.983  < 0.001 | 61.392  < 0.001 |
| **Group (GO, SGO, RASIG)*Country** | 29.443  0.001 | 28.148  0.001 | 28.584  0.001 | 27.347  0.001 | 27.708  0.001 | 27.602  0.001 | 28.494  0.001 | 28.278  0.001 | 28.857  0.001 | 28.014  0.001 | 29.241  0.001 | 21.320  0.011 |
| **Gender** | 0.126  0.722 | 0.005  0.943 | 0.189  0.664 | 0.062  0.803 | 0.061  0.806 | 0.001  0.982 | 0.022  0.882 | 0.027  0.869 | 0.013  0.910 | 0.038  0.846 | 0.004  0.947 | 2.040  0.153 |
| **Lymphocyte to monocyte** | 41.695  <0.001 | 39.344  <0.001 | 43.418  <0.001 | 41.915  <0.001 | 43.528  <0.001 | 42.158  < 0.001 | 40.354  < 0.001 | 25.879  < 0.001 | 39.984  < 0.001 | 31.717  < 0.001 | 42.557  < 0.001 | 13.149  0.004 |
| **Vegetables consumption** |  |  |  |  |  |  |  |  |  |  |  | 0.587  0.746 |
| **Fruit consumption** |  |  |  |  |  |  |  |  |  |  |  | 1.175  0.556 |
| **Dairy Products** |  |  |  |  |  |  |  |  |  |  |  | 6.871  0.032 |
| **French fries** |  |  |  |  |  |  |  |  |  |  |  | 4.252  0.119 |
| **Brown Bread Consumption** |  |  |  |  |  |  |  |  |  |  |  | 0.637  0.727 |
| **White bread consumption** |  |  |  |  |  |  |  |  |  |  |  | 3.152  0.207 |
| **Alcohol consumption** | 2.584  0.275 |  |  |  |  |  |  |  |  |  |  | 2.071  0.355 |
| **Glucose** |  | 1.035  0.793 |  |  |  |  |  |  |  |  |  | 0.068  0.995 |
| **Glycosylated haemoglobin** |  |  | 6.059  0.109 |  |  |  |  |  |  |  |  | 7.651  0.054 |
| **Homocysteine** |  |  |  | 6.619  0.085 |  |  |  |  |  |  |  | 7.718  0.052 |
| **MCH** |  |  |  |  | 6.633  0.085 |  |  |  |  |  |  | 3.925  0.270 |
| **MCV** |  |  |  |  |  | 3.075  0.380 |  |  |  |  |  | 0.393  0.942 |
| **White blood cells** |  |  |  |  |  |  | 5.474  0.140 |  |  |  |  | 3.461  0.326 |
| **Monocytes** |  |  |  |  |  |  |  | 2.163  0.539 |  |  |  | 0.594  0.898 |
| **Lymphocytes** |  |  |  |  |  |  |  |  | 2.036  0.565 |  |  | 3.315  0.346 |
| **Neutrophils** |  |  |  |  |  |  |  |  |  | 10.251  0.017 |  | 7.200  0.066 |
| **Basophils** |  |  |  |  |  |  |  |  |  |  | 4.383  0.223 | 4.659  0.199 |

^1^ Analysis was performed by GLM; all 2-way interactions with group (GO, SGO and RASIG) were tested and those not significant were removed from the models. All variables were included as categorical variables based on results of the Kruskal-Wallis test. Definition of abbreviations is provided in the supplementary list.

**Supplementary list of abbreviations and their definition**

**Abbreviation Definition**

BMI Body Mass Index

CD3+CD45+ % CD3+ of viable CD45+ cells (Total T cells)

HCT Haematocrit

HDL High Density Lipoprotein

HGB Haemoglobin

LDL Low Density Lipoprotein

MCH Mean Corpuscular Haemoglobin

MCHC Mean Cell Haemoglobin Concentration

MCV Mean Corpuscular Volume

MPV Mean Platelet Volume

RBC Red Blood Cells

RDW Red Blood Cell Distribution Width

WBC White Blood Cells
